# Myosin inhibition partially rescues the myofiber proteome in X-linked myotubular myopathy

**DOI:** 10.1172/jci.insight.194868

**Published:** 2025-11-04

**Authors:** Elise Gerlach Melhedegaard, Fanny Rostedt, Charlotte Gineste, Robert A.E. Seaborne, Hannah F. Dugdale, Vladimir Belhac, Edmar Zanoteli, Michael W. Lawlor, David L. Mack, Carina Wallgren-Pettersson, Anthony L. Hessel, Heinz Jungbluth, Jocelyn Laporte, Yoshihiko Saito, Ichizo Nishino, Julien Ochala, Jenni Laitila

**Affiliations:** 1 Department of Biomedical Sciences, University of Copenhagen, Copenhagen, Denmark.; 2 Folkhälsan Research Center, Helsinki, Finland.; 3 Department of Medical Genetics, University of Helsinki, Helsinki, Finland.; 4 IGBMC, CNRS UMR 7104, Inserm U 1258, Strasbourg University, Illkirch, France.; 5 Centre of Human and Applied Physiological Sciences, School of Basic and Medical Biosciences, Faculty of Life Sciences & Medicine, King’s College London, London, United Kingdom.; 6 School of Sport, Exercise and Health Sciences, Loughborough University, Loughborough, United Kingdom.; 7 Department of Neurology, Faculdade de Medicina, Universidade de São Paulo, São Paulo, Brazil.; 8 Diverge Translational Science Laboratory and Medical College of Wisconsin, Department of Pathology and Laboratory Medicine, Milwaukee, Wisconsin, USA.; 9 Institute for Stem Cell and Regenerative Medicine,; 10 Department of Bioengineering, and; 11 Department of Rehabilitation Medicine, University of Washington, Seattle, Washington, USA.; 12 Institute of Physiology II, University of Muenster, Muenster, Germany.; 13 Accelerated Muscle Biotechnologies Consultants, Boston, Massachusetts, USA.; 14 Department of Paediatric Neurology, Neuromuscular Service, Evelina London Children’s Hospital, Guy’s & St Thomas’ NHS Foundation Trust, London, United Kingdom.; 15 Randall Centre for Cell and Molecular Biophysics, Muscle Signalling Section, Faculty of Life Sciences and Medicine, King’s College London, London, United Kingdom.; 16 Department of Neuromuscular Research, National Institute of Neuroscience, National Center of Neurology and Psychiatry, Tokyo, Japan.

**Keywords:** Metabolism, Muscle biology, Molecular pathology, Muscle, Neuromuscular disease

## Abstract

X-linked myotubular myopathy (XLMTM) due to *MTM1* mutations is a rare and often lethal congenital myopathy. Its downstream molecular and cellular mechanisms are currently incompletely understood. The most abundant protein in muscle, myosin, has been implicated in the pathophysiology of other congenital myopathies. Hence, in the present study, we aimed to define whether myosin is also dysfunctional in XLMTM and whether it, thus, may constitute a potential drug target. To this end, we used skeletal muscle tissue from patients and canine/mouse models; we performed Mant-ATP chase experiments coupled with x-ray diffraction analyses and LC/MS-based proteomics studies. In patients with XLMTM, we found that myosin molecules are structurally disordered and preferably adopt their ATP-consuming biochemical state. This phosphorylation-related (mal)adaptation was mirrored by a striking remodeling of the myofiber energetic proteome in XLMTM dogs. In line with these, we confirmed an accrued myosin ATP consumption in mice lacking MTM1. Hence, we treated these with a myosin ATPase inhibitor, mavacamten. After a 4-week treatment period, we observed a partial restoration of the myofiber proteome, especially proteins involved in cytoskeletal, sarcomeric, and energetic pathways. Altogether, our study highlights myosin inhibition as a potentially new drug mechanism for the complex XLMTM muscle phenotype.

## Abstract 2

The most abundant muscle protein, myosin, maladapts and becomes very energy-consuming in X-linked myotubular myopathy. Inhibiting its ATPase activity partly rescues the molecular defects.

## Graphical abstract

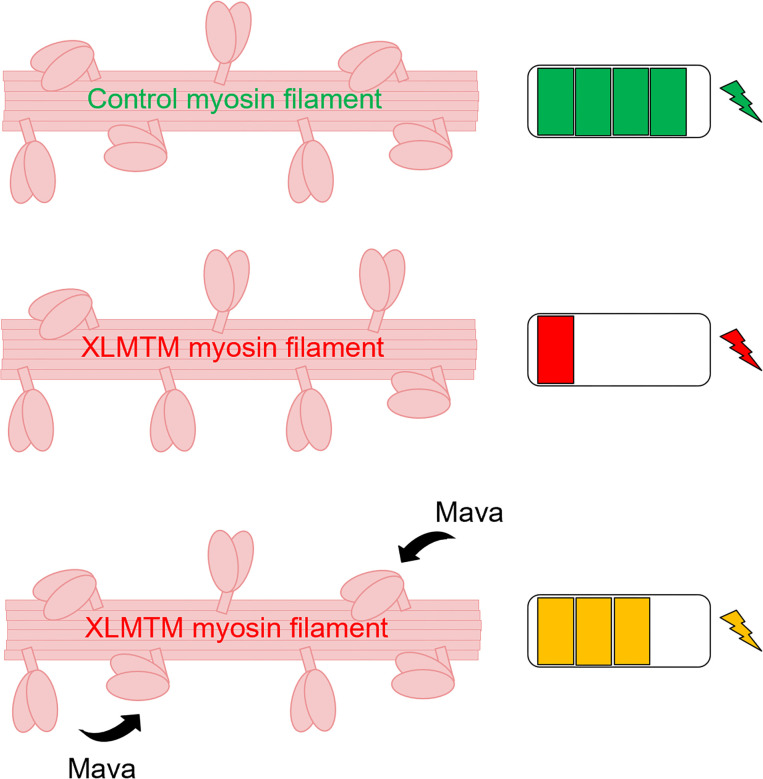

## Introduction

X-linked myotubular myopathy is a congenital myopathy caused by hemizygous mutations in the X-chromosomal *MTM1* gene and affects approximately 1:50,000 males (XLMTM, OMIM 310400) (1, 2). While it is well established that XLMTM-linked mutations lead to changes in the myotubularin protein causing severe early-onset skeletal muscle phenotypes, the exact downstream molecular and cellular pathogenic mechanisms are complex and remain incompletely understood (3, 4). Pathophysiological categories identified to date include, at least, neuromuscular junction alterations, excitation-contraction coupling changes, reduced muscle fiber size, secondary mitochondrial abnormalities, and aberrant autophagy (3, 5, 6). Unexpectedly, our laboratory has recently identified myosin, the most abundant striated muscle protein, as a potentially underappreciated contributor (7).

Besides binding to actin monomers and initiating force production and motion (8), myosin molecules have at least 2 relaxed biochemical states known as super-relaxed (SRX) and disordered-relaxed (DRX) (9, 10). Structurally, SRX is often associated with myosin heads being in an “OFF” state and being anchored to the filament backbone, while DRX is related to myosin molecules not being immobilized in an “ON” state with a potential to weakly bind to actin monomers (9, 10). Energetically, SRX and DRX states differ in their ATP demand, with DRX having an ATPase activity up to 10 times greater than SRX (9, 10). Various other congenital myopathies have already been associated with altered myosin biochemical states, such as nemaline myopathy, a disease often due to mutations in the *NEB* and *ACTA1* genes. There, the amount of myosin molecules in the ATP-demanding DRX has been found higher than normal, affecting the basal skeletal muscle ATP usage and, in turn likely contributing to the muscle fiber energy proteome remodeling and potentially to muscle fatigue (11, 12). In *RYR1*-related congenital myopathies, one of the most common forms of congenital myopathy, a faster ATP turnover time of myosin heads in DRX has also been identified (13). Consequently, in the present study, we first sought to identify whether similar myosin-centered pathogenic (or compensatory) alterations are also prevalent in XLMTM. For that, we utilized samples from both humans with XLMTM and a canine model. This XLMTM animal model expresses a *MTM1* gene mutation (p.N155K in myotubularin) that induces a rapidly progressing inability to stand, walk, and feed after an initial period of normal development (14, 15). Here, we also sought to determine whether targeting myosin as a therapeutic intervention for XLMTM is viable energetically. For that, we treated mice lacking MTM1 (and experimentally mimicking XLMTM) with a compound known to inhibit myosin SRX/OFF state and related ATP consumption, mavacamten (also termed MYK-461 or Camzyos) (16, 17).

## Results

### Disproportionate number of muscle myosin molecules in the ATP-consuming DRX state in patients with XLMTM.

**Figure 1:**
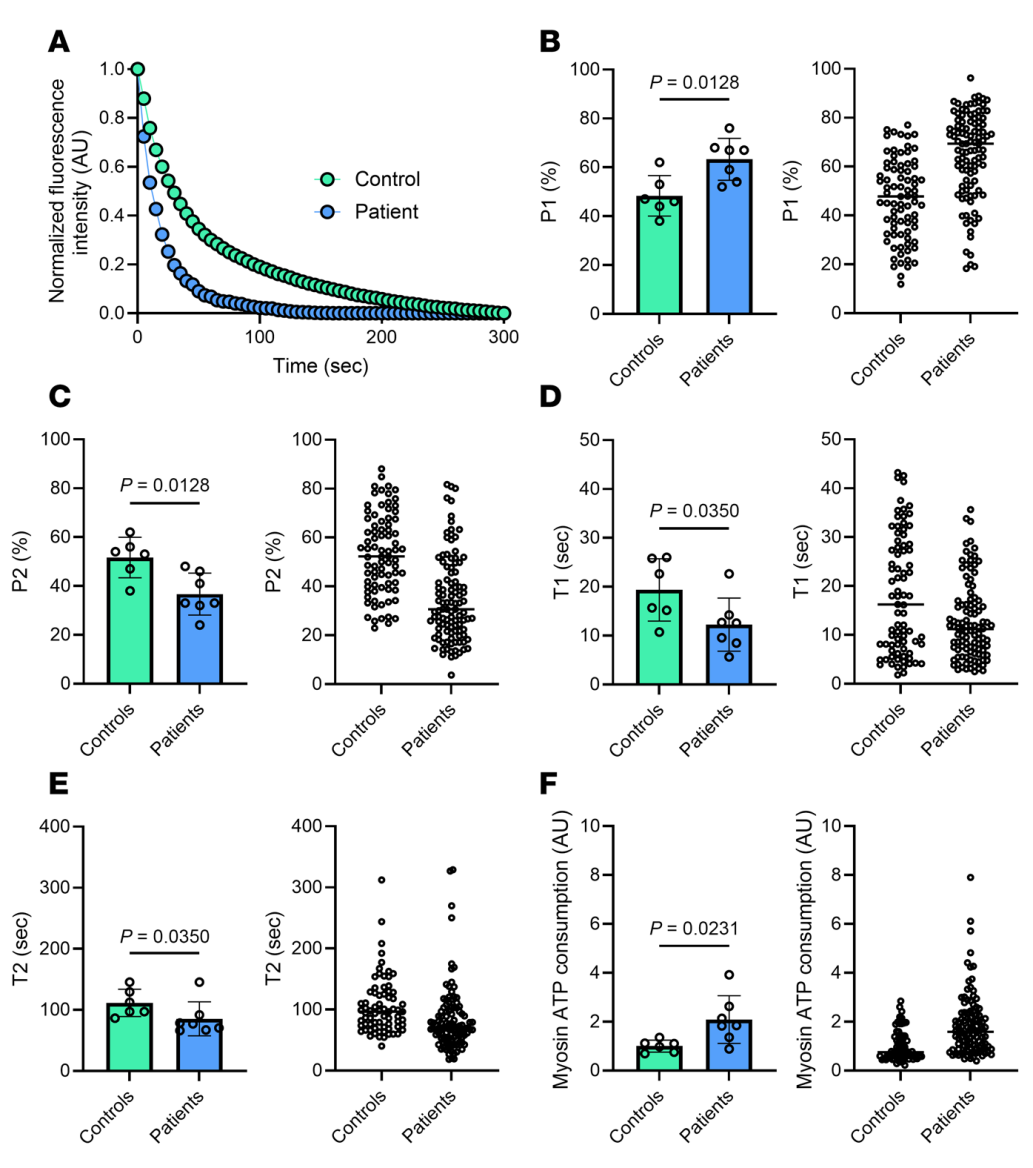
Myosin biochemical and energetic states in patients with XLMTM. (**A**) Typical fluorescent decays for one XLMTM patient and one control. (**B**) The proportion of myosin molecules in the DRX state (P1). (**C**) The number of myosin heads in the SRX state (P2) for all patients and fibers analyzed. (**D** and **E**) Their respective ATP turnover lifetimes (T1 in **D** and T2 in **E**). (**F**) The theoretical myosin ATP consumption based on P1, P2, T1 and T2 values. For **B**–**F**, in the left panels, circles are individual patient’s average data; while in the right panels, circles are individual muscle fibers. Data are shown as mean ± SD. Unpaired *t* tests with Welch correction were used to compare groups (level of significance *P* < 0.05).

To determine muscle myosin DRX and SRX states, we performed Mant-ATP chase experiments (13). Mant-ATP [2’-(or-3’)-*O*-(*N*-Methylanthraniloyl) Adenosine 5’-Triphosphate] was used to determine the fluorescence intensity decay over time and was best fitted by a double exponential curve allowing the separation of fast and slow phases (indicative of DRX and SRX states, respectively) as the rate of ATP consumption is approximately 10-fold greater for DRX than SRX (Figure 1A). In the present study, a total of 188 myofibers were assessed (minimum of 7 muscle fibers for each of the patients with XLMTM and controls). P1 (indicative of DRX) was greater in muscle fibers from patients with XLMTM than controls (Figure 1B), whereas P2 (indicative of SRX) was smaller in myofibers from patients with XLMTM than controls (Figure 1C). In line with these (mal)adaptations, T1 and T2 (indicative of the ATP turnover of myosin molecules in DRX or SRX states, respectively) were faster in myofibers from patients with XLMTM than controls (Figure 1, D and E). Taking into consideration P1, P2, T1, and T2, we estimated a theoretical myosin ATP consumption. This theoretical myosin energy expenditure was 2-fold greater in muscle fibers from patients with XLMTM than controls (Figure 1F).

**Figure 2:**
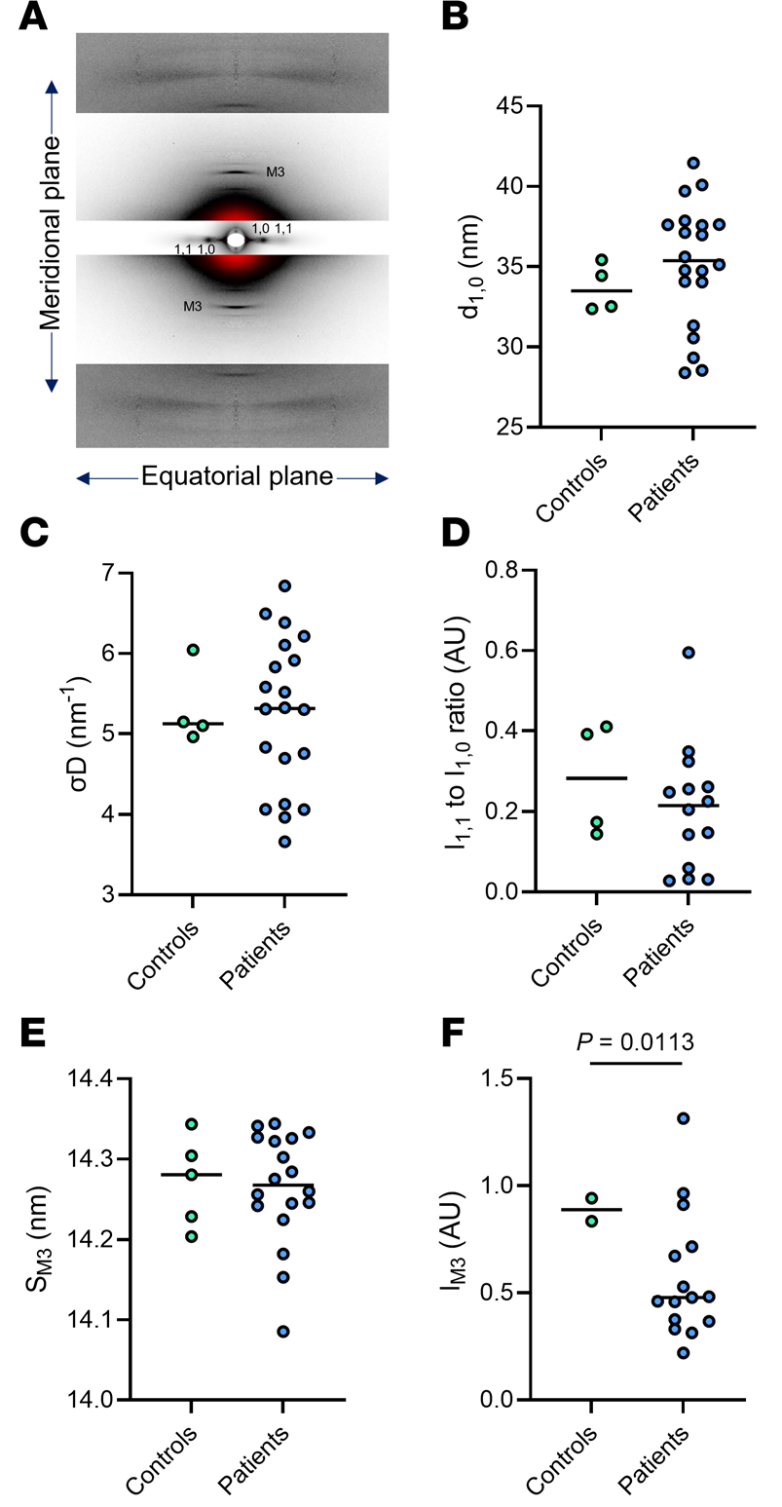
Myofilament and myosin order in patients with XLMTM. (**A**) A typical x-ray diffraction pattern from one control. Equatorial (1,0 and 1,1) as well as meridional (M3) reflections are presented. (**B**) Interfilament lattice spacing derived from the 1.0 equatorial reflections (d1,0). (**C**) The heterogeneity of inter-filament lattice spacing (σD). (**D**) The equatorial intensity ratio (I_1,1_ to I_1,0_ ratio); (**E**) is M3 spacing (S_M3_). (**F**) M3 intensity (I_M3_). Circles are individual muscle bundles. Means represented by a line also appear on all the panels. Unpaired *t* tests with Welch correction were used to compare groups (level of significance *P* < 0.05).

To identify whether these myosin biochemical alterations are accompanied by a change in the structural ON and OFF states, we assessed x-ray diffraction patterns of thin bundles from patients with XLMTM and controls (Figure 2A). We analyzed equatorial reflections related to the lattice planes creates by myosin (1,0 plane) and both myosin and actin filaments (1,1 plane); and meridional reflections related to the ~14.3 myosin crown repeats (M3). The interfilament lattice spacing (d_1,0_; Figure 2B), its heterogeneity (σD; Figure 2C), and equatorial intensity ratio (I_1,1_ to I_1,0_ ratio; Figure 2D) were not different between patients with XLMTM and controls, suggesting an unaffected myofilament lattice organization. Nevertheless, the meridional M3 reflection (representative of the distance between myosin crowns) showed some (mal)adaptations in the diseased group. Indeed, despite a preserved spacing (S_M3_; Figure 2E), the M3 intensity was lower in myofibers from patients with XLMTM than controls (I_M3_; Figure 2F). This indicates that myosin heads may prefer a structural disordered (and potentially ON) state. Altogether, our loaded Mant-ATP chase experiments and x-ray diffraction analyses have revealed that there is an accrued proportion of muscle myosin molecules in the DRX state, significantly increasing the ATP demand in patients with XLMTM.

### Phosphorylation-linked disruption of the number of myosin heads in the ATP-demanding DRX state in XLMTM dogs.

**Figure 3:**
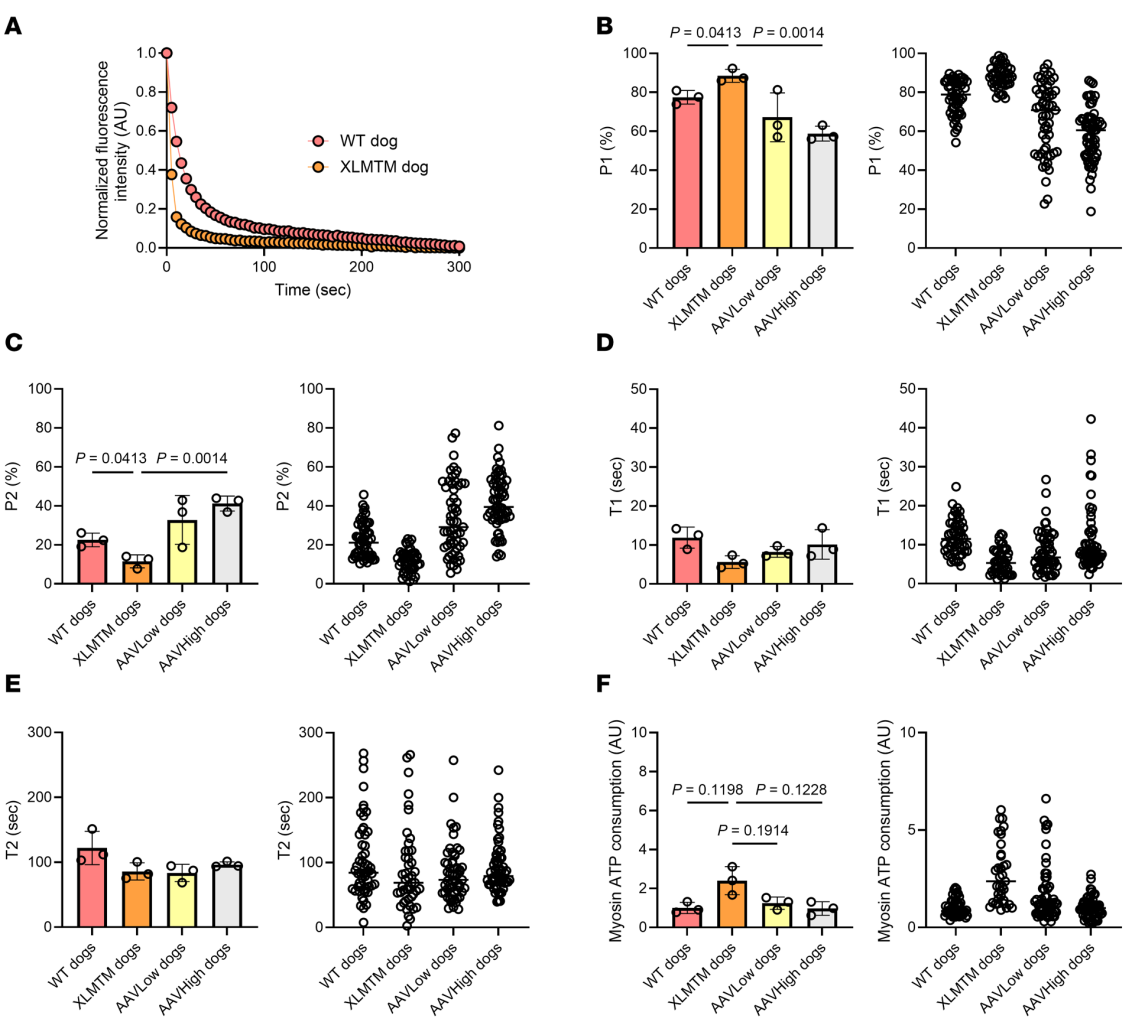
Myosin biochemical and energetic states in the canine model of XLMTM. Our study included WT control dogs as well as XLMTM-affected dogs expressing a *MTM1* gene mutation. These XLMTM dogs were injected systemically with a saline solution (XLMTM) or with 2 different doses of gene therapy (AAV^lo^ or AAV^hi^). (**A**) Typical fluorescent decays for one XLMTM dog and one WT. (**B**) The amount of myosin molecules in the DRX state (P1). (**C**) The proportion of myosin heads in the SRX state (P2). (**D** and **E**) Their respective ATP turnover lifetimes are also displayed (T1 in **D** and T2 in **E**). (**F**) The theoretical myosin ATP consumption. For **B**–**F**, in the left panels, circles are individual animals’ average data; in the right panels, circles are individual muscle fibers. Data are shown as mean ± SD. One-way ANOVAs with Tukey post hoc were used to compare groups (level of significance *P* < 0.05).

To get insights into potential underlying mechanisms (and as we did not have enough human material), we took advantage of a canine model carrying a *MTM1* gene mutation (14, 15). We separated and tested a total of 190 muscle fibers (minimum of 15 myofibers per animal). We used WT controls and XLMTM dogs. To evaluate whether myosin-related changes are profound or can ultimately be reversed, we also performed our Mant-ATP assays on dogs injected with 2 different doses of a gene therapy consisting of rAAV8 vector expressing the WT canine *MTM1* cDNA under the control of the desmin promoter (AAV^lo^ and AAV^hi^). AAV^hi^ dogs are known for being functionally cured, while AAV^lo^ animals have heterogenous and variable responses to the treatment. This may be related to the fact that some of the dogs display little improvements in the amount of myotubularin while others experienced a significant increase in lifespan (18, 19). When analyzing the Mant-ATP chase experiments, the change of fluorescence intensity over time was best fitted by a double exponential decay (Figure 3A). As with the patients with XLMTM, P1 was higher in myofibers from XLMTM dogs than control (WT) animals (Figure 3B). In parallel, P2 was smaller in muscle fibers from XLMTM dogs than WT animals (Figure 3C). Interestingly, P1 (and P2) were fully rescued in AAV^hi^ dogs (Figure 3, B and C). Unlike humans, we only detected tendencies toward a faster T1 in XLMTM dogs compared with WT animals, and we detected a partial restoration in AAV^hi^ animals when compared with XLMTM dogs (Figure 3D). T2 was not affected by XLMTM or the gene therapy (Figure 3E). When taking P1, P2, T1, and T2 together, the theoretical myosin ATP expenditure tended to be greater in muscle fibers from XLMTM dogs than WT animals (Figure 3F). This tendency disappeared in AAV^hi^ dogs (Figure 3F).

**Figure 4:**
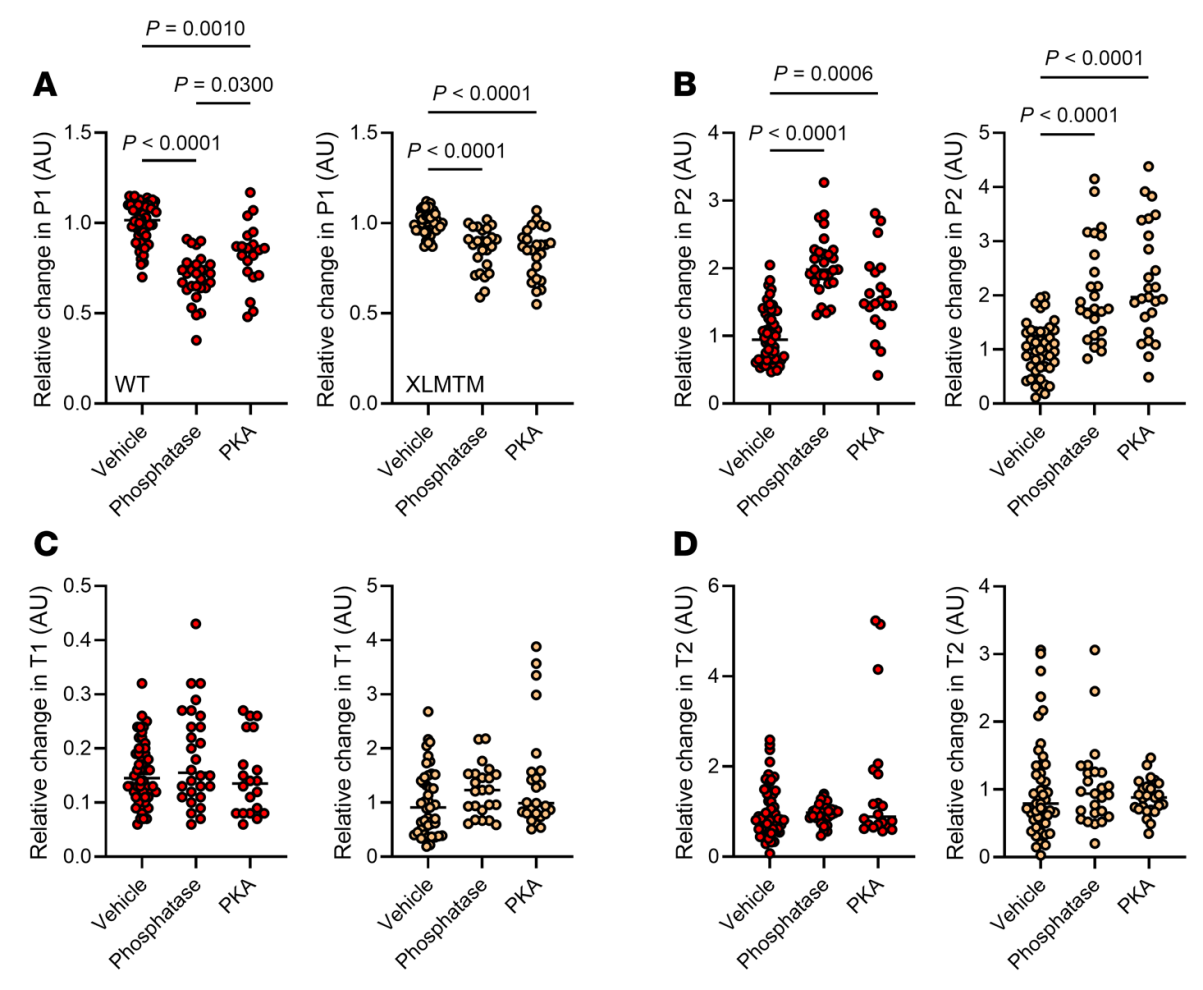
Effect of phosphorylation level on myosin biochemical states in XLMTM dogs. (**A** and **B**) The relative change in the number of myosin molecules in the DRX (P1) and SRX state (P2), respectively, in response to vehicle, phosphatase treatment or protein kinase A (PKA) incubation. (**C** and **D**) The relative change of their respective ATP turnover time (T1 and T2, respectively). For **A**–**D**, The left panels show WT; the right panels show XLMTM dogs. Circles are individual muscle fibers. Means represented by a line also appear on all the panels. One-way ANOVAs with Tukey post hoc were used to compare groups (level of significance *P* < 0.05).

As the level of phosphorylation of muscle myosin molecules is essential for superrelaxation (10), we sought to characterize whether this specific posttranslational modification plays a role in what we describe above — i.e., myosin (mal)adaptation in XLMTM. We isolated muscle fibers from the canine model (total of 198 muscle fibers). In vitro incubation of muscle fibers with a lambda phosphatase solution decreased P1 (and increased P2) in both XLMTM dogs and WT animals (Figure 4, A and B). However, the effect of the phosphatase treatment was more pronounced in the WT compared with the XLMTM dogs (Figure 4, A and B). No effects on T1 or T2 was observed (Figure 4, C and D). Treating muscle fibers with a protein kinase A (PKA) solution partially restored P1 (and P2) in WT dogs but not in XLMTM dogs (Figure 4, A and B). As for the phosphatase incubation, PKA had no effect on T1 or T2 (Figure 4, C and D). Thus, modulating the level of myofilament phosphorylation affects the equilibrium between DRX and SRX states in a differential manner in XLMTM dogs and WT animals. Overall, our Mant-ATP chase experiments in myofibers from a canine model of XLMTM indicate that, in the context of the disease, myosin biochemical (mal)adaptation is related to phosphorylation alterations and is reversible.

### Disrupted myosin DRX state and ATP consumption are paralleled with a muscle fiber proteome remodeling in XLMTM dogs.

**Figure 5:**
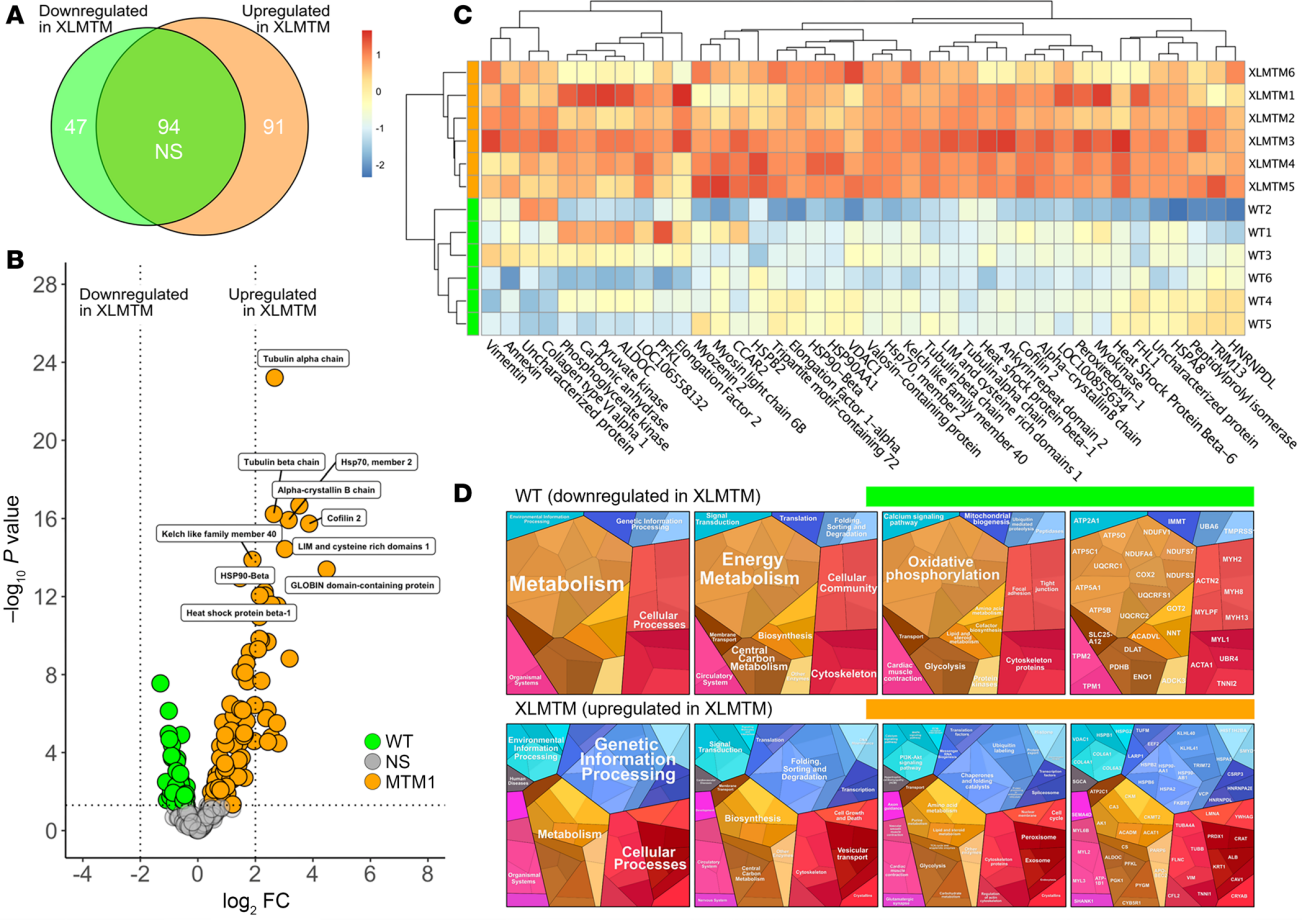
Comparative proteomic analysis in XLMTM dogs. (**A**) Venn diagram depicting detected proteins that were significantly upregulated and downregulated in XLMTM as well as those that remained statistically unchanged between experimental groups. (**B**) Volcano plot displaying Log_2_ fold change (Log_2_FC) against Log_10_ *P* value. The top 10 most significant proteins have been annotated. (**C**) A heatmap was created to illustrate the proteins with the greatest fold change, all proteins included possessed a Log_2_FC > 1.5. For readability some gene names are used rather than protein names; the protein and gene names can be found in Supplemental Table 2. (**D**) Proteomaps were generated using Log_2_ transformed protein abundance values of proteins, which were significantly different between groups. Unpaired 2-tailed *t* test was used.

Since muscle fibers from XLMTM dogs have an accrued number of myosin molecules in the ATP-demanding DRX state, we sought to gain insights into a potential remodeling of cellular energy proteome. We separated individual myofibers from the canine model and ran a proteomics analysis using quantitative LC-MS/MS. Fiber type for each sample was established, and all samples were considered to be mixed type I/2A (Supplemental Table 1; supplemental material available online with this article; https://doi.org/10.1172/jci.insight.194868DS1). Following data filtration, 232 proteins were considered for comparison between WT and XLMTM dogs (Supplemental Table 2). Of the 232 proteins, 138 were significantly different between groups, with approximately two-thirds being upregulated in the XLMTM group (47 down- and 91 upregulated; Figure 5A). A volcano plot was generated to visualize the 232 proteins. The top 10 most significant proteins were annotated, revealing that not only does XLMTM have a greater number of upregulated proteins, but the top 10 most significant proteins were all upregulated in the XLMTM group (Figure 5B). Tubulin alpha chain possessed the highest significance value, whereas GLOBIN domain–containing protein had the highest fold change (both upregulated in XLMTM). All significant proteins observed with a log_2_FC > 1.5 were visualized in a heatmap (Figure 5C). This further illustrated the large shift in proteins upregulated in the XLMTM group. While these figures provide visualization of differential change in the proteome, they do not provide insight as to the potential pathways modified as a result of or causing the aforementioned ATP demand. To examine this, we carried out gene ontology (GO) analysis using Proteomaps (Figure 5D). Proteomaps highlighted a modification in metabolic proteins (both up- and downregulated). While proteins associated with oxidative phosphorylation appeared to be largely downregulated in XLMTM, the presence of proteins required for similar energetic pathways (i.e., glycolysis, lipid, steroid, and amino acid metabolisms) may be indicative of a general metabolic disruption, rather than a compensation toward a different energetic pathway. This disruption may be seen as an exhaustion of the oxidative machinery (where the increased myosin ATP consumption may have played a role). Furthermore, and perhaps unsurprisingly, cytoskeletal proteins were dysregulated. Similarly to the energetic pathways, these proteins were dysregulated in both directions (up- and downregulated in XLMTM).

### Myosin inhibition partially restores the dysregulated myofiber proteome in mice lacking MTM1.

**Figure 6:**
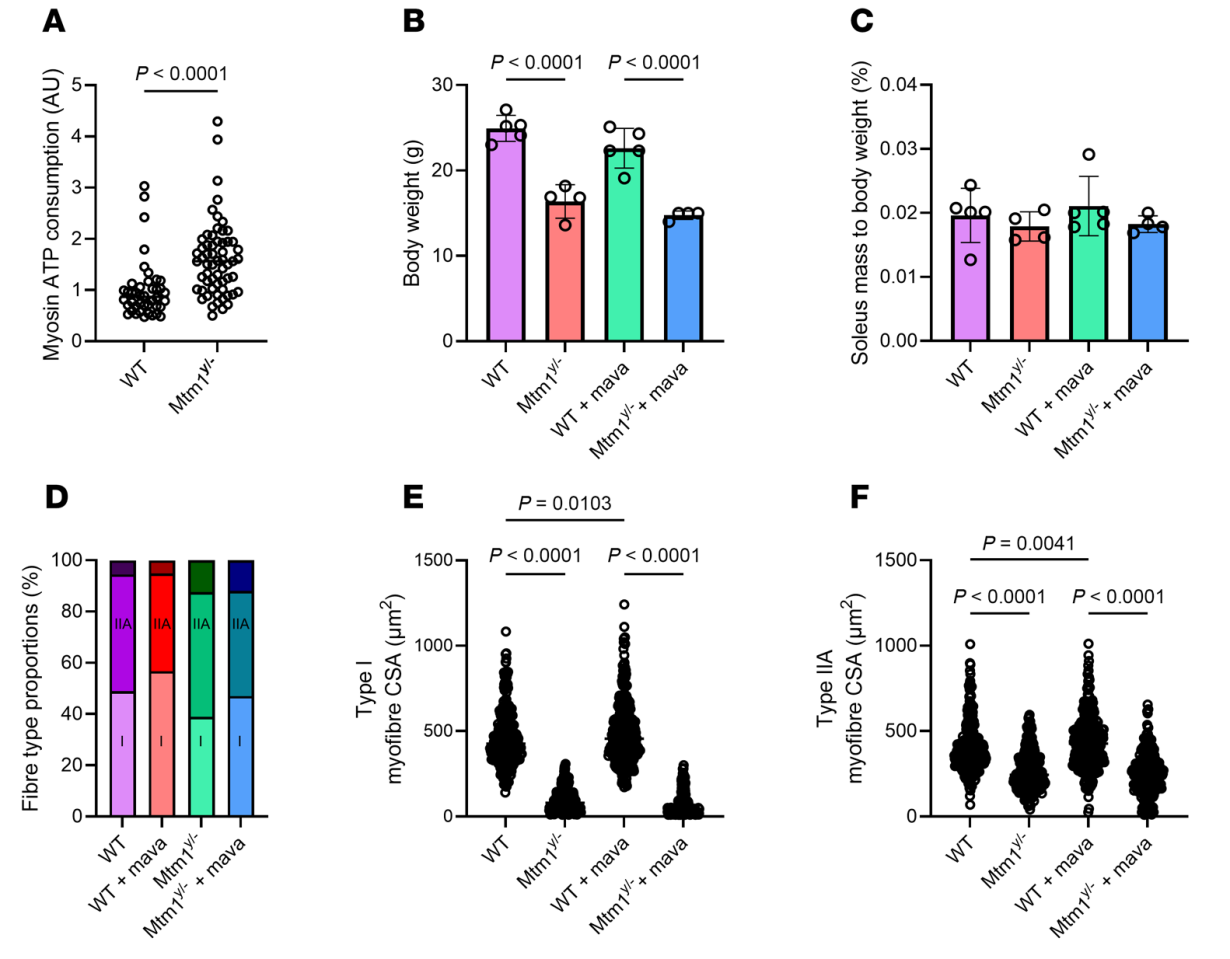
Muscle histological analysis in mice lacking MTM1. Our study involved WT mice as well as mice lacking MTM1 (*Mtm1^y/–^*). These animals were either treated with vehicle or mavacamten for 4 weeks (+ mava). (**A**) The theoretical myosin ATP consumption. (**B**) Body weight. (**C**) Soleus muscle mass normalized to body weight. (**D**) Fiber type proportions. (**E** and **F**) Myofiber cross-sectional area separated according to their types (I vs IIA). For **A**, **E**, and **F**, circles are individual myofibers’ data; an unpaired *t* test with Welch correction was used to compare groups (level of significance *P* < 0.05). For **B** and **C**, circles are individual animals’ data; data are shown as mean ± SD; 2-way ANOVA with Tukey post hoc were used to compare groups (level of significance *P* < 0.05).

**Figure 7:**
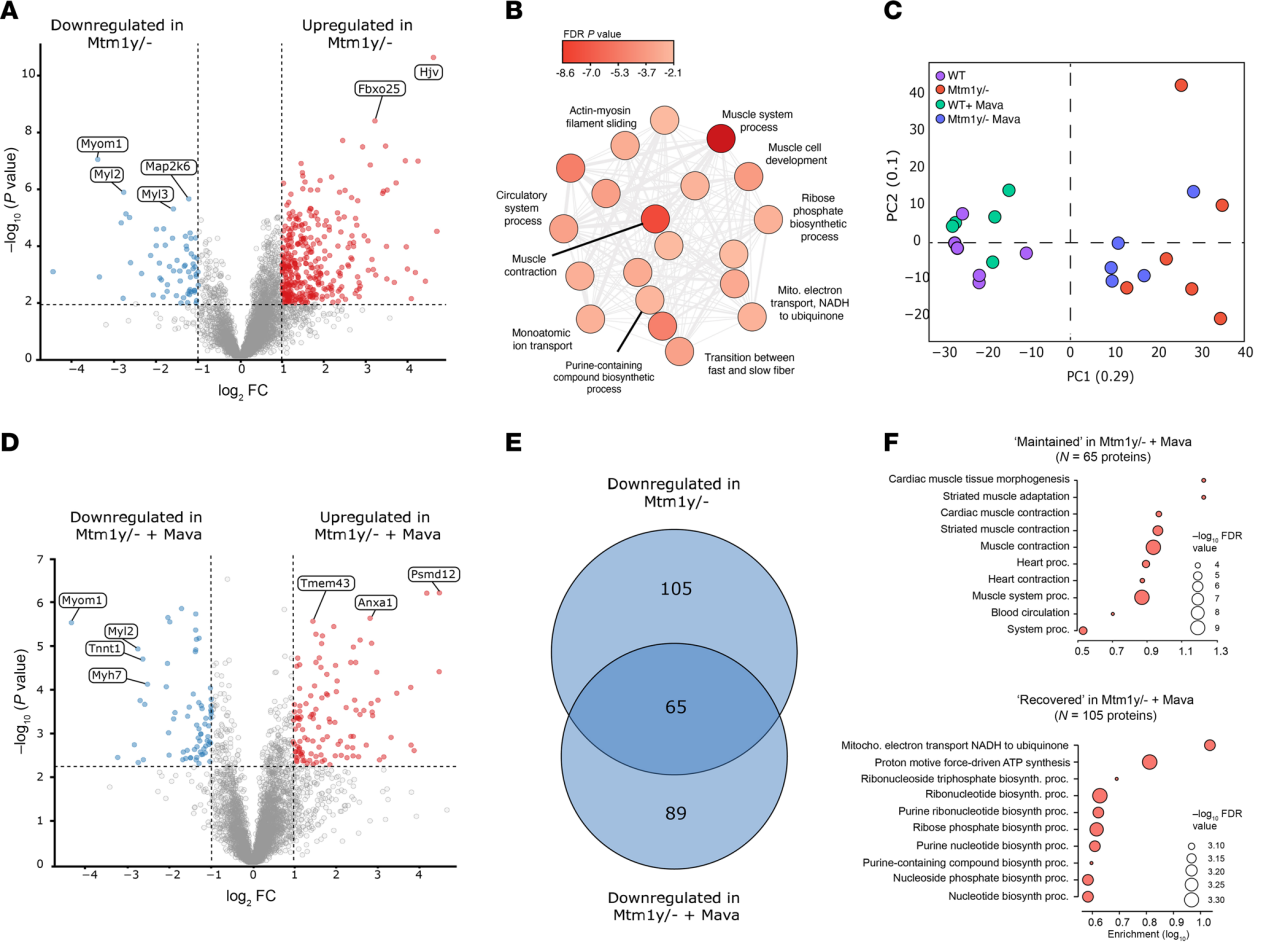
Comparative proteomic analysis in mice lacking MTM1. We used WT mice and mice lacking MTM1 (*Mtm1^y/–^*). These animals were either treated with vehicle or mavacamten for 4 weeks (+ mava). (**A**–**F**) Part of the proteomics analyses with the principal component analyses and volcano plots displaying Log_2_ fold change against Log_10_ *P* value.

To unveil whether these basic energetic alterations of myosin molecules can be alleviated, we used a mouse model lacking MTM1 (20), where the calculated myosin ATP expenditure is significantly greater than normal (Figure 6A), mimicking human XLMTM (Figure 1F). Body weight was significantly lower in untreated *Mtm1^y/–^* mice than controls (WT) (Figure 6B). After a 4-week exposure to a myosin ATPase inhibitor named mavacamten (12, 21), body weight was not affected by the treatment in either *Mtm1^y/–^* or WT mice (Figure 6B). Soleus muscle mass (normalized to body weight) and fiber type proportions did not appear different between the 4 groups (Figure 6, C and D). As expected, myofiber cross-sectional area was smaller in both type I and IIA of *Mtm1^y/–^* mice than WT (Figure 6, E and F). This latter was significantly greater after the mavacamten treatment in both type I and IIA of WT mice only (Figure 7, A and B).

To explore potential differences at the protein level, we enriched soleus muscle fibers and performed global proteomic profiling. In the first instance, we found a significant dysregulation in the soleus muscle proteome of *Mtm1^y/–^* mice, with 907 protein hits displaying differential abundance (Figure 7A and Supplemental Table 3). Interestingly, while the majority of these proteins were found to be more abundant in *Mtm1^y/–^* mice (737 proteins, ~81%), the only evidential coordination in dysregulation was found in downregulated proteins (170 hits). Indeed, when performing enrichment GO analysis for biological processing terms, these proteins all cluster to terms relating to the sarcomere, muscle contraction, myofiber biology/physiology, and metabolic/energy processing (Figure 7B). Such a response demonstrates a disruption in the fundamental properties of the soleus muscle in *Mtm1^y/–^* mice. It also strengthens our canine proteomics analysis where similar clusters of dysregulated protein were shown (Figure 5).

When examining the effect of 4-week mavacamten treatment on mouse soleus muscle, we first noticed that *Mtm1^y/–^* mice tended to transition toward a WT proteome with a noticeable reduction in heterogeneity, as demonstrated via PCA (Figure 7C). We explored the proteome of these mice and found a reduced number of differential proteins following mavacamten treatment (410 proteins dysregulated in total; 153 downregulated, 257 upregulated in *Mtm1^y/–^* mavacamten; Figure 7D and Supplemental Table 4), in comparison with our untreated mice comparison. Comparing the downregulated proteins in the 2 analyses (with and without mavacamten) the downregulated proteins shared 65 proteins maintaining consistent dysregulation, while 105 proteins were uniquely dysregulated in the untreated *Mtm1^y/–^* mice (Figure 7D). This suggests that the abundance of these proteins is restored to nonsignificant levels relative to WT mice, following 4-weeks mavacamten treatment. Enrichment GO analysis of both the restored 105 proteins, as well as the maintained 65 proteins identified terms relating to sarcomere, myofiber biology/physiology, and metabolism (Figure 7, E and F, and Supplemental Table 5). Collectively, these analyses suggest that 4-week treatment with mavacamten is able to partially restore the dysregulated sarcomeric and energetic proteome in *Mtm1^y/–^* mouse soleus muscle.

Of note, 89 protein hits were found to be significantly downregulated following mavacamten treatment in *Mtm1^y/–^* mice; these hits were not found in the original *Mtm1^y/–^* mice comparison. GO analysis revealed little coordination in these proteins.

## Discussion

Our findings highlight that myosin molecules, within isolated muscle fibers from patients, from a canine model with *MTM1* gene mutations, and from a mouse model lacking MTM1, have increased ATP consumptions. In line with these observations, our results demonstrate that the myofiber energetic (and cytoskeletal) proteome undergoes a maladaptive remodeling in dogs carrying a p.N155K mutation in myotubularin. Nevertheless, and of great interest, in MTM1-deficient mice, a short-term treatment with mavacamten, pharmacological compound known to rescue myosin biochemical state, partially counterbalanced the altered expression of myofiber proteins belonging to cytoskeletal, sarcomeric, and energetic pathways.

### Preferred myosin DRX state in XLMTM.

Our result of a greater proportion of myosin molecules in the ATP-demanding DRX state is perfectly in line with our recent works focusing on another form of congenital myopathy, nemaline myopathy (11, 12). Even though nemaline myopathy belongs to the same class of skeletal muscle diseases, causative mutations mainly alter genes encoding proteins of the sarcomere, including *NEB* (nebulin) and *ACTA1* (actin), as opposed to XLMTM, where mutations affect genes with no known direct links to myosin (*MTM1*; myotubularin) (2, 3, 5). Hence, the present study strongly indicates that myosin dynamics (mal)adaptation is common to multiple forms of congenital myopathies and occurs irrespective of the genes mutated. Future studies will have to decipher whether this change is primarily pathogenic or secondary. We can speculate that it may act as a compensatory/remodeling process by which myosin heads enter their DRX/ON state to be more available for actin binding and then counteract the cellular force depression seen in XLMTM (3). In the long-term, we can further speculate that this predominant myosin DRX/ON state may become energetically deleterious with an unwanted increase in basal sarcomeric ATP usage (contributing to muscle fatiguability).

The exact triggers by which myosin function is disrupted in XLMTM remain in question. However, our canine proteomics analyses did not reveal any major quantitative adaptations of the main proteins involved in the stabilization of myosin SRX/OFF state (e.g., myosin heavy chain, and myosin binding protein-C) (10). On the other hand, more subtle changes are likely to happen and may involve posttranslational modifications such as phosphorylation, as proven by our series of in vitro experiments. Indeed, when we modulated the phosphorylation levels within isolated muscle fibers using phosphatase or PKA incubation, we were able to worsen or rescue the myosin dynamics response. Note that these modulations (incubations) were unspecific and mainly targeted sarcomeric phosphorylatable proteins, such as myosin regulatory light chains, myosin binding protein C, and the troponin complex (22). These 3 proteins possess large amounts of the amino acids serine, threonine, and tyrosine; they also have a turnover rate of 1%–2% per day, making them preferential targets for phosphorylation (23–25). If, in XLMTM, the phosphorylation levels of regulatory light chains and protein C are impaired (e.g., Ser15 and Ser282, respectively), one may suggest that the myosin head-head and head-tail interactions, characteristic of the interacting-heads motif, are weaker (10). This may, in turn, cause myosin heads to move away from the thick filament backbone, reducing head ordering, and inhibiting the energy-conserving configuration.

Is myotubularin deficiency involved in fine-tuning myosin and myosin-binding proteins phosphorylation? If the increased proportion of myosin heads in their DRX/ON state is a compensatory/remodeling mechanism (as mentioned above), this is unlikely. However, if this phenomenon is primarily pathogenic of XLMTM, we can suggest some potential molecular processes. Myotubularin is known to primarily function as a lipid phosphatase modulating membrane trafficking and signaling lipid pools (20). Then, it may affect protein phosphorylation indirectly through its regulation of lipid signaling molecules. This has been proven for the phosphorylation status of important muscle proteins such as Akt (26). Further experiments specifically and thoroughly assessing the link between myotubularin and the posttranslational status of the proteins mentioned above are warranted.

### Consequences of myosin energy dissipation in XLMTM.

The shift toward more disordered ATP-dissipating myosin molecules undoubtedly induces an increase in basal sarcomeric energy expenditure in XLMTM. Does this myosin (mal)adaptation have whole-body consequences? Skeletal muscle together with liver, brain, heart, adipose tissue, and kidneys contribute substantially to the basal metabolic rate (27, 28). Because skeletal muscle represents 45%–55% of total body mass, it contributes to 20%–30% of whole-body resting oxygen uptake and, thus, basal metabolic rate (29–31). Within skeletal muscle (and myofibers), besides myosin ATPase, the sarcoplasmic reticulum Ca^2+^ ATPase as well as Na^+^/K^+^ ATPase have been shown to extensively account for the organ resting energy expenditure (8). Nevertheless, it is known that shifting myosin molecules from their ATP-conserving to their ATP-consuming conformation by approximately 10% leads to an increase in energy demand by 0.7 megajoule day^−1^ (10). Over a period of a year, this induces a weight loss of 7 kg of fat in humans (10). Consequently, in the present study, we can speculate that the 2-fold increase in myosin ATP consumption may increase the whole-body basal metabolic rate and decrease fat content, contributing to the fact that patients are lean or underweight. Additionally, based on our previous study (11, 12), in the long-term, we believe this has serious consequences by straining energy resources and exhausting cellular respiration. This notion is strengthened by our canine proteomic analyses where we found a clear downregulation in the abundance of proteins involved in energy metabolism and oxidative phosphorylation.

### Myosin inhibition as a drug target in XLMTM.

Based on the above results, designing a therapeutic intervention that would decrease myosin ATPase activity would be obvious and worth exploring in the context of XLMTM. In the present study, we focused our attention on 1 pharmacological compound named mavacamten (or MYK-461/Camzyos). Mavacamten is well established to inhibit myosin ATP turnover rate by pushing heads toward their quiescent SRX/OFF state, consequently reducing the basal sarcomeric ATP expenditure (16, 17). Its efficacy has been proven in cardiac muscle of patients with hypertrophic cardiomyopathy (32). Interestingly and as demonstrated here, when applied short-term in mice lacking myotubularin, myosin inhibition did partially rescue the muscle fiber molecular signature by notably restoring the expression of a number of proteins belonging to the cytoskeletal, sarcomeric, and energetic pathways. Our proof-of-concept study provides a strong basis for future works where myosin inhibition may be sustained for longer periods of time or at higher doses. We can speculate that these may allow a full recovery of the myofiber proteome. Could mavacamten be directly used in clinical trials focusing on XLMTM? As this drug has been developed to specifically target β-cardiac/slow myosin heavy chain, it may not be as potent in fast-twitch muscle fibers expressing type IIa and IIx myosin heavy chains. Mavacamten may also lead to unwanted effects on patients’ hearts. Hence, the present study highlights myosin as a drug target (rather than mavacamten per se).

### Cross-species similarities and differences.

Interestingly, here, we have unveiled a common pathway by which muscle myosin and its ATP consumption (mal)adapt in all the species studied: mice, dogs, and humans. Nevertheless, one should be aware that the underlying molecular pathogenic or compensatory/remodeling mechanisms leading to such myosin disruption may have some species-specific components. Indeed, genetic mutations, protein variants, and myotubularin expressions differ between humans, dogs, and mice. For instance, dogs have a p.N155K variant resulting in a partial protein deficiency (18, 19), while mice have a deletion inducing a frameshift, premature stop codon, and total absence of myotubularin (20). Human mutations have variable molecular consequences (Table 1). Future studies deciphering the exact upstream mechanisms by which differential myotubularin expressions affect myosin molecules (and their phosphorylation status) are needed.

### Conclusion.

Altogether, by isolating membrane-permeabilized myofibers from patients with XLMTM, and from dog/mouse models of the disease, we uncovered a change in myosin dynamics as attested by the presence of an unusually high proportion of proteins in their ATP-dissipating state, DRX. We also highlighted the role of one specific posttranslational modification, phosphorylation, in such a process. Finally, in line with this increased molecular energy expenditure, we delineated an adaptation of the muscle fiber energetic (and cytoskeletal) proteome that can partially be rescued by mavacamten short-term. All these data emphasize that myosin, besides being a molecular contributor to XLMTM, can be considered as a suitable target for future drug design.

## Methods

### Sex as a biological variable

XLMTM is characterized by profound muscle weakness with marked respiratory impairment and profound bulbar involvement in males (1, 2). For this reason, the present study focused its attention on males.

### Patients

Vastus lateralis muscle biopsy specimens had been obtained as part of the normal diagnostic process from patients with *MTM1* mutations (*n* = 7) and controls with no history of neuromuscular disease (*n* = 6). Details of all the individuals are given in Table 1. All samples were flash frozen and stored at –80°C until analyzed.

### Canine model

Dogs (males) used in the current work have been part of a previous study (18, 19). Briefly, XLMTM-affected dogs carried a *MTM1* gene mutation with a p.N155K variant in myotubularin, leading to a partial protein deficiency (18, 19). These animals were bred in a colony located at the University of Washington. XLMTM dogs were injected systemically with either a saline solution (XLMTM, *n* = 3) or with 2 different doses of a rAAV8 vector expressing the wild-type canine *MTM1* cDNA (rAAV8-cMTM1): 5.0 × 10^12^ (AAV^lo^, N = 3) and 8.0 × 10^13^ (AAV^hi^, *n* = 3) vg.kg^–1^ at the age of 10 weeks. All animals, including WT control dogs (*n* = 3) were sacrificed between 39 and 41 weeks of age (end of the study). Biceps femoris specimens were subsequently obtained for further analysis.

### Mouse model

MTM1-deficient mice (*Mtm1^–/y^*) and WT mice in 129Pas background were generated and identified through PCR genotyping from mouse tail DNA as previously described (20). WT littermates served as controls. Mice were housed in an environment-controlled facility (12–12 h light-dark cycle, 22°C) and received water and food ad libitum. *Mtm1^–/y^* and WT mice received either mavacamten at a dose of 2.5 mg/kg of mouse/day in drinking water or placebo (i.e. drinking water) for 4 weeks starting at 4 weeks of age (12). At 8 weeks, mice were anesthetized by i.p. injection of domitor/fentanyl mix (2/0.28 mg/kg) and diazepam (8 mg/kg). Soleus muscles were harvested, weighed, and flash-frozen in liquid nitrogen before being stored at –80°C for later analysis. Mice were euthanized by cervical dislocation.

### Solutions

Relaxing solution contained 4 mM Mg-ATP, 1 mM free Mg^2+^, 1 × 10^–6^ mM free Ca^2+^, 10 mM imidazole, 7 mM EGTA, 14.5 mM creatine phosphate, and KCl to adjust the ionic strength to 180 mM and pH to 7.0. Additionally, the rigor buffer for Mant-ATP chase experiments contained 120 mM K acetate, 5 mM Mg acetate, 2.5 mM K_2_HPO_4_, 50 mM MOPS, and 2 mM DTT with a pH of 6.8. The lambda phosphatase solution (New England Biolabs) was prepared into the relaxing solution to yield 4 U lambda phosphatase/μL while the PKA solution was also prepared into the relaxing buffer to reach 0.5 U PKA/μL (33).

### Muscle preparation and fiber permeabilization

Small pieces of the cryopreserved human, canine, and mouse muscle samples were immersed in a membrane-permeabilizing solution (relaxing solution containing glycerol; 50:50 v/v) for 24 hours at –20°C, after which they were transferred to 4°C for an additional 24 hours (to allow for a proper skinning/membrane permeabilization process). After these steps, the muscle pieces were stored in the same buffer at –20°C for use up to 3 weeks (13).

### Mant-ATP chase experiments

On the day of the experiments, the muscle pieces were transferred to relaxing solution, and single myofibers were manually isolated. Their ends were individually clamped to half-split copper meshes designed for electron microscopy (SPI G100 2010C-XA, width, 3 mm), which had been glued onto glass slides (Academy, 26 × 76 mm, thickness 1.00–1.20 mm). Cover slips were then attached to the top (using double-sided tape) to create flow chambers (Menzel-Glaser, 22 × 22 mm, thickness 0.13–0.16 mm) (13). Muscle fibers were mounted at a slack length of approximately 2.20 μm (the sarcomere length was checked using the bright-field mode of a Zeiss Axio Scope A1 microscope). Similarly to previous studies (11, 12), all experiments were performed at 25°C, and each fiber was first incubated for 5 minutes with a rigor buffer. A solution containing the rigor buffer with 250 μM Mant-ATP was then flushed and kept in the chamber for 5 minutes. At the end of this step, another solution made of the rigor buffer with 4 mM unlabeled ATP was added with simultaneous acquisition of the Mant-ATP chase.

For fluorescence acquisition, a Zeiss Axio Scope A1 microscope was used with a Plan-Apochromat 20×/0.8 objective and a Zeiss AxioCam ICm 1 camera. Frames were acquired every 5 seconds for the first 90 seconds and every 10 seconds for the remaining time with a 20 ms acquisition/exposure time using a DAPI filter set, and images were collected for 5 minutes. For each individual myofiber, areas of at least 1,000 pixels were sampled for fluorescence decay using the ROI manager in ImageJ (NIH) as previously published (13). The mean background fluorescence intensity was subtracted from the average of the fiber fluorescence intensity (for each image taken). Each time point was then normalized by the fluorescence intensity of the final Mant-ATP image before washout (T = 0). These data were then fit to an unconstrained double exponential decay using GraphPad Prism 10.3:

*Normalized Flourescence* = 1 − *P*_1_(1 − *exp*^(−*t*⁄*T*1)^) − *P*_2_(1 − *exp*^(−*t*⁄*T*2)^)

Where P1 is the amplitude of the initial rapid decay approximating DRX with T1 as the time constant for this decay. P2 is the slower second decay approximating SRX with its associated time constant T2. In the present article, P1, P2, T1 and T2 are presented without any correction for the rapid wash-out of nonspecific binding of Mant-ATP (13). Solely based on P1, P2, T1, and T2, we estimated the theoretical ATP consumed by myosin using the following equation and the assumption that the concentration of myosin molecules within myofibers is 220 μM (10).

Theoretical myosin ATP consumption per cell per min = *P*_1_/100 ∙ 220 ∙ 60/*T*_1_ + *P*_2_/100 ∙ 220 ∙ 60/*T*_1_

### Immunofluorescence staining and imaging

For a subset of muscle fibers used for the Mant-ATP chase experiments, we defined the type of myosin heavy chain using immunofluorescence staining as previously detailed (13). Briefly, an anti–β-cardiac/skeletal slow myosin heavy 7 antibody (IgG1, A4.951, sc-53090 from Santa Cruz Biotechnology, dilution: 1:50) together with a goat anti–mouse IgG1 Alexa 555 (from ThermoScientific, dilution 1:1,000) were used. As previously seen (13), as we did not detect any fiber-type specific differences in either control or diseased samples, and as myofiber heterogeneity is multi-dimensional with sources of variation extending far beyond myosin heavy chain isoforms (34), we pooled all the fiber types together (Supplemental Figure 1).

### X-ray diffraction recordings and analysis

On the day of the experiments, sections were transferred to relaxing solution, and muscle bundles were manually isolated in relaxing buffer. Next, the distal and proximal ends were wrapped with 6.0 silk suture and then transferred to the experimental apparatus. Small angle x-ray diffraction was conducted at the MiNaXS (P03) beamline at the Petra III Synchrotron, German Electron Synchrotron Source (DESY). A custom muscle mechanics rig was set up in line of the x-ray beam (0.82 A), similar to those performed elsewhere (35). Each sample was attached to the rig and set at a sarcomere length approximating 2.20 μm (36). During experiments, x-rays passed through the sample perpendicular to the long axis of the sarcomeres and diffracted, with the diffraction pattern collected by a Pilatus 1M detector (Dectris LTD, Switzerland) ~9.8 meters downstream of the sample. Samples were maintained in a bath of relaxing solution at 27°C, but during exposures, the bath was automatically removed by a motor to allow for x-ray exposure “in air,” significantly improving the signal (<30s in air to maintain hydration). Multiple x-ray exposures were collected per sample, with no more than 1-second exposure on any sample location, which maximized signal quality while maintaining sample integrity. All diffraction patterns were aligned and merged together for each sample; this improved the signal-to-noise ratio. All x-ray data reduction and analyses were carried out using custom-build Python code and the freeware MuscleX (BioCAT, Advanced Photon Source, Argonne National Laboratory, USA). To avoid any artifacts originating from the size or the structural organization of the myofibers, diffraction intensities (for each exposure) were normalized to each other via the direct beam intensity (measured at the detector).

### Global proteome profiling of canine skeletal muscle

#### LC-MS/MS identification and quantitative analysis of protein abundance.

Because the skeletal muscle niche containing an array of cell types analyzing bulk tissue may lead to interference from nonmuscle cells, we focused our analyses on myofibers. As previously described (11, 12, 37), approximately 20 muscle fibers were manually isolated from each dog sample and placed into a single centrifuge tube containing 30 μL Tris-Triton lysis buffer (10 mM Tris, pH 7.4, 100 mM NaCl, 1 mM EDTA, 1 mM EGTA, 1% Triton X-100, 10% glycerol, 0.1% SDS, 0.5% deoxycholate, protease inhibitor cocktail III [1:100], phosphatase inhibitor cocktail mix [1:100]). Sample volume was reduced by half in a SpeedVac (Thermo Fisher Scientific) and subsequently mixed in a 1:1 ratio with Laemmli buffer (2× conc.), vortexed and boiled at 96°C for 10 minutes. To stack the protein complement and remove chemical interference from the lysis buffer, samples were centrifuged at 14,000 rpm for 3 minutes prior to loading in 10% BisTris NuPAGE gel. Protein bands were visualized using the Imperial protein stain. In-gel reduction, alkylation, and digestion with trypsin was performed prior to subsequent isobaric mass tag labeling. Each sample was treated individually with labels (tandem mass tag [TMT] 6-plex) added at a 1:1 ratio (38). A mixture of 3 disease and 3 control samples was used within each 6-plex.

TMT-labeled peptide samples were resuspended in 60 μL of resuspension buffer (2 % acetonitrile in 0.05% trifluoroacetic acid) and analyzed by LC-MS/MS. Chromatographic separations were performed using an Ultimate 3000 UHPLC system (Thermo Fisher Scientific). In triplicate samples, a 10 μL injection of peptides was resolved by reversed phase chromatography on a 75 μm C18 column (50 cm) using a 3-step linear gradient of acetonitrile in 0.1% formic acid. The gradient was delivered to elute the peptides at a flow rate of 250 nL/min over 120 minutes. The eluate was ionized by electrospray ionization using an Orbitrap Fusion Lumos (Thermo Fisher Scientific) operating under Xcalibur v4.1. The instrument was programmed to acquire in automated data-dependent switching mode, selecting precursor ions based on their intensity for sequencing by higher-energy C-trap dissociation (HCD) for peptide identification and reporter ion fragmentation. Selection of precursor ions based on their intensity for sequencing was done by HCD in a TopN method. The MS/MS analyses were conducted using higher than normal collision energy profiles that were chosen based on the mass-to-charge ratio (m/z) and the charge state of the peptide. To increase fragmented peptide coverage and reporter ion intensities, a further Synchronous Precursor Scan (SPS) of the Top 5 most intense peaks using MS3 was performed.

#### Database searching.

Raw mass spectrometry data from the triplicate injection were processed into peak list files using the Proteome Discoverer (ThermoScientific; v2.2) (PD 2.2). The raw data file was processed and searched using the Mascot search algorithm (v2.6.0; www.matrixscience.com) and the Sequest search algorithm against the canine database curated within Uniprot taxonomy database (38).

Within the consensus processing module, the reporter ion intensity values (absolute area under the peak) for each peptide spectral match were grouped with peptides and calculated at the protein level identification as a grouped abundance. All grouped abundances at protein level were normalized using total peptide amount, which has previously been corrected based on the highest peptide abundance present in 1 channel; thus, all channels have the same total abundance.

#### Bioinformatics and data visualizations.

Following processing with Proteome Discoverer, the resultant file was exported into Perseus (v1.6.3) for qualitative and quantitative data analysis. Further data visualization was achieved using R, version 4.2.1 (2022-06-23). WT and XLMTM mean protein abundance was used to visualize composition of protein functions using an automatic system based on KEGG Pathways gene classifications, named proteomaps (39). Proteomaps requires conversion to relevant associated gene, the majority of these genes were obtained using Uniprot and Gene ID Conversion Tool available via DAVID Bioinformatics (40).

#### Fiber type estimation.

Previously defined by Murgia and coworkers (41, 42), for each sample the abundance of Myosin heavy chains 1, 2, 4, and 7 (MYH1, -2, -4, and -7) was summed, and the expression of each isoform was then calculated as a percentage. Pure fibers were expressed ≥ 80% of a particular MHY protein; for example, those samples possessing ≥ 80% of MYH7 were classified as a pure slow fiber. Where the 80% expression threshold was not met, the fiber was classified as a mixed fiber. Here the highest and second highest percentage contribution providing the classification — i.e., MYH7 and MHY2 classified as a Mixed type I/2A fiber. Each sample group contained 20 individual fibers; therefore, the fiber type was estimated based on the relative myosin expression for each sample, not each fiber.

### Mouse sectioning and cross-sectional area measurements

Immunolabeling was conducted on 10 μm cryosections, which were initially fixed with 4% paraformaldehyde for 10 minutes, permeabilized using 0.1% Triton X-100 for 20 minutes, and subsequently blocked with 10% Normal Goat Serum (50062Z; Life Technologies, Carlsbad, CA, USA) containing 0.1% bovine serum albumin for 1 hour. The sections were then incubated O/N at 4°C with a primary antibody targeting Laminin (rabbit polyclonal L9393 Sigma, St. Louis, MO, USA; 1:20 dilution). Alexa Fluor Donkey anti-Rabbit 488 (A11034; Life Technologies; RRID: AB_2576217; 1:500 dilution) was utilized as the secondary antibody in 10% Normal Goat Serum (Life Technologies). For fiber typing, Zenon Mouse IgG1 Labeling Kits with Alexa Fluor 488 (Z25002; Thermo Fisher Scientific, Waltham, MA, USA) and Alexa Fluor 647 (Z25008; Thermo Fisher Scientific) were implemented to label MYH I (mouse monoclonal A4.951; Developmental Studies Hybridoma Bank (DSHB), Iowa City, IA, USA; RRID:AB_2147279; 1:25 dilution) and MYH IIA (mouse monoclonal SC71, DSHB; RRID:AB_2147165; 1:25 dilution), respectively. These labeling complexes were applied in a solution of 5% goat serum with 0.1% bovine serum albumin and 0.1% Triton X-100 and incubated for 2 hours at RT. A 10× objective on a Zeiss Axio Observer 3 fluorescence microscope with a Colibri 5 led detector, combined with a Zeiss Axiocam 705 mono camera, using Zen software (Zeiss, Oberkochen, Baden-Württemberg, Germany) was used to take fluorescence images. The images were exported to single channel.tiff files. Fiber-membrane (laminin) stained single images were loaded into ilastik, version 1.4.0.post1-OSX (www.ilastik.org) and the software was trained to differentiate muscle fibers from their membrane. The results were exported from ilastik as binary H5 files with the simple segmentation setting and analyzed in Fiji (ImageJ, version 1.54g, with the ilastik Fiji plugin; https://imagej.net) with a macro using the analysis particle function and ROI manager tool. In short, a ROI-set with individual ROIs outlining the perimeter of the muscle fibers was automatically generated by the macro. A visual inspection of the ROI-set was conducted, and errors were subsequently corrected through manual adjustments, after which the cross-sectional areas were exported to Excel (Microsoft Corp., Redmond, WA, USA). Fiber type was manually assigned to the unique ID of each ROI in the Excel file (43, 44).

### Global proteome profiling of mouse skeletal muscle

As the skeletal muscle niche contains an array of cell types that may lead to nonmyofiber specific data sets when analyzing bulk tissue (45), we enriched for myofibers in our mouse proteomic experiments. Dissected soleus muscle specimens (*n* = 5 or 6 per condition) were retrieved from the –80°C freezer and placed in 0.2% collagenase (C5138; Sigma) at 37°C for 60–90 minutes and routinely disturbed to enable disruption. Samples were transferred to a sterilized 6-well plate, agitated to further dissociate the sample and individual myofibers were manually transferred to ice-cold PBS under a dissection microscope. An estimated 50 myofibers were withdrawn from 6-well plate, washed through with PBS for a second time, snap-frozen on dry ice and stored at –80°C. These were then processed as in (46, 47). Briefly, they were lysed with lysis buffer (1% (w/v) sodium deoxycholate, 100 mM Teab, pH 8.5) and incubated for 10 minutes at 95°C followed by sonication using a Bioruptor pico (30 cycles, 30 sec on/off, ultra-low frequency). Heat incubation and sonication were repeated once, samples cleared by centrifugation, reduced with 5 mM (final concentration) of TCEP for 15 min at 55°C, alkylated with 20 mM (final concentration) CAA for 30 minutes at RT, and digested adding Trypsin/LysC at 1:100 enzyme/protein ratio. Peptides were separated on an Aurora (Gen3) 25 cm, 75 μM ID column packed with C18 beads (1.7 μm) (IonOpticks) using a Vanquish Neo (Thermo Fisher Scientific) UHPLC. Peptide separation was performed using a 90-minute gradient of 2%–17% solvent B (0.1% formic acid in acetonitrile) for 56 minutes, 17%–25% solvent B for 21 min, 25%–35% solvent B for 13 minutes, using a constant flow rate of 400 nL/min. Column temperature was controlled at 50°C. MS data were acquired with a timsTOF HT (Bruker Daltonics) in diaPASEF mode. MS data were collected over a 100–1,700 m/z range. During each MS/MS data collection each PASEF cycle was 1.8 seconds. Ion mobility was calibrated using 3 Agilent ESI-L Tuning Mix ions 622.0289, 922.0097, and 1221.9906. For diaPASEF we used the long-gradient method which included 16 diaPASEF scans with two 25 Da windows per ramp, mass range 400.0–1201.0 Da and mobility range 1.43-0.6 1/K_0_. The collision energy was decreased linearly from 59 eV at 1/K_0_ = 1.6 to 20 eV at 1/K_0_ = 0.6 Vs cm^–2^. Both accumulation time and PASEF ramp time was set to 100 ms.

#### Proteome profiling analysis.

All statistical analysis of protein expression intensity data was performed using in-house Python code, developed from the automated analysis pipeline of the Clinical Knowledge Graph as previously detailed (48). Proteins referring to potential contaminants, identified by matches to the decoy reverse database, and identified only by modified sites, were removed. LFQ intensities were Log_2_ transformed with proteins with more than 0l5 missing values in at least one group were filtered out. Missing values were imputed using a mixed imputation method. That is, on a per protein basis, missing values in samples belonging to the same comparative group were KNN imputed if at least 60% valid values existed in that same group. Thereafter, remaining missing values were imputed using the MinProb approach (width = 0.3, shift = 1.8), as previously described (49). Unpaired 2-tailed *t* test comparisons across relevant conditions were performed in Perseus (50), with differentially expressed proteins considered statistically significant following Benjamini-Hochberg multiple correction at an FDR level of 0.05 (and fold-change of 2). ShinyGO (v0.82) was used for enrichment analyses of all statistically significant protein hits, with the background set as all proteins identified in the original *Mtm1^y/–^* and WT mouse comparison data set (4009 proteins). Significantly enriched terms were identified using hypergeometric tests, containing at least 5 targets within the term and following multiple correction adjustment (Benjamini-Hochberg).

### Statistics

Data are shown as mean ± SD. For all nonprotein data sets, graphs were generated in GraphPad Prism v10.4. Statistical significance was set to *P* < 0.05. Student’s *t* tests with Welsh corrections or 1- or 2-way ANOVA together with Tukey post hoc analyses were run to compare groups.

### Study approval

Investigations included human, dog, and mouse muscle samples. For human tissue: (a) all clinical information and materials were obtained for diagnostic purposes; (b) informed consent had been obtained from all the participants; (c) procedures conformed to the standards set by the latest version of the Declaration of Helsinki; and (d) the study was approved by local ethics committee (National Center of Neurology and Psychiatry no. A2020-068). For canine tissue, all experiments were handled according to principles outlined in the *Guide for the Care and Use of Laboratory Animals* (National Academies Press, 2011). For mouse tissue, experimentation was approved by the institutional ethics committee in accordance with French and European legislation (project no. 30860-2021040115344997).

### Data availability

All the data analyzed are presented here in the figures and/or supplementary files, including the Supporting Data Values file. Note that the mass spectrometry proteomics data have been deposited to the ProteomeXchange Consortium via the PRIDE partner repository with the dataset identifier PXD068387.

## Author contributions

EGM, FR, CG, J Laporte, JO, and J Laitila contributed to the study conception and design. Material preparation, data collection, and analysis were performed by EGM, FR, CG, RAES, HFD, VB, EZ, MWL, DLM, CWP, ALH, HJ, J Laporte, YS, IN, JO, and J Laitila. The first draft of the manuscript was written by EGM, FR, JO, and J Laitila, and all authors commented on all versions of the manuscript and approved of the submitted version.

## Funding support

This work is the result of NIH funding, in whole or in part, and is subject to the NIH Public Access Policy. Through acceptance of this federal funding, the NIH has been given a right to make the work publicly available in PubMed Central.

- Lundbeckfonden (grant agreement no. R434-2023-311 to JO)
- Novo Nordisk Foundation (grant agreement no. NNF21OC0070539 to JO and grant NNF19SA0059305 to core facilities)
- AFM-Téléthon (grant agreement no. 23933 to J Laporte)
- Finska Läkaresällskapet to FR
- The Jane and Aatos Erkko Foundation (grant agreement no. 220029 to FR and J Laitila).

## Supplementary Material

Supplemental data

Supporting data values

## Figures and Tables

**Table 1:**
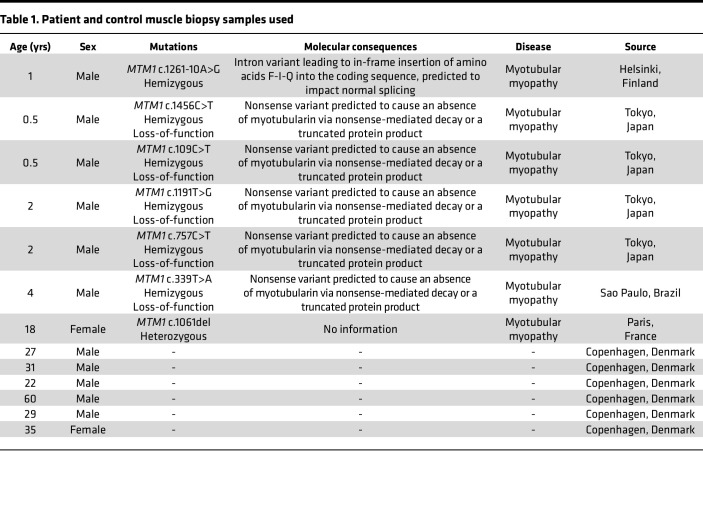
Patient and control muscle biopsy samples used

## Version 1 11/04/2025

In-Press Preview

## Version 2 12/22/2025

Electronic publication
