# Supplementary material for: Myosin inhibition partially rescues the myofiber proteome in X-linked myotubular myopathy
Source: JCI Insight. 2025 Nov 4;10(24):e194868. doi: 10.1172/jci.insight.194868 (PMC12890516; doi:10.1172/jci.insight.194868)

Supplementary file for:

Myosin inhibition partially rescues the muscle fibre molecular phenotype in X-linked myotubular myopathy

Elise Gerlach Melhedegaard, Fanny Rostedt, Charlotte Gineste, Robert A.E. Seaborne, Hannah F. Dugdale, Vladimir Belhac, Edmar Zanoteli, Michael W. Lawlor, David L. Mack, Carina Wallgren-Pettersson, Anthony L. Hessel, Heinz Jungbluth, Jocelyn Laporte, Yoshihiko Saito, Ichizo Nishino, Julien Ochala, Jenni Laitila

This document contains:
Supplemental Tables 1 to 5;
Supplemental Figure 1.

Supplemental Table 1: Fibre type proportions in the samples used for canine proteomics.

	Percentage myosin				
	Myosin 2	Myosin-1	Myosin-7	Myosin-4	Fibre type
Control 1	64.26	5.66	26.77	3.31	Mixed slow/2A
Control 2	58.62	5.14	33.49	2.76	Mixed slow/2A
Control 3	45.63	2.19	51.25	0.93	Mixed slow/2A
Control 4	66.61	2.32	30.63	0.43	Mixed slow/2A
Control 5	44.04	1.87	53.56	0.53	Mixed slow/2A
Control 6	91.07	2.66	5.86	0.41	2A
XLMTM 1	55.78	1.76	41.86	0.60	Mixed slow/2A
XLMTM 2	75.49	4.48	17.64	2.39	Mixed slow/2A
XLMTM 3	45.00	2.60	51.18	1.22	Mixed slow/2A
XLMTM 4	51.62	6.63	36.83	4.91	Mixed slow/2A
XLMTM 5	33.17	2.90	62.07	1.87	Mixed slow/2A
XLMTM 6	26.75	3.85	66.67	2.72	Mixed slow/2A

Supplemental Table 2: Canine global untargeted proteomics analysis. Significant upregulation in each experimental group determined based on *p* < 0.05.

Protein name	*Uniprot Accession*	*Gene name*	*Gene ID*	*Control*	*XLMTM*	*Log₂ FC*	*p-value*	*q value*
**Upregulated in XLMTM**								
GLOBIN domain-containing protein	A0A8C0P013			51.667	894.500	4.479	< 0.001	< 0.001
Cofilin 2	A0A8I3MEH0	CFL2	490649	321.833	4489.444	3.867	< 0.001	< 0.001
Alpha-crystallin B chain	A0A8C0Q8G4	CRYAB	479441	1854.111	21608.778	3.527	< 0.001	< 0.001
Cell cycle and apoptosis regulator 2	A0A8I3P633	CCAR2	486122	345.944	2314.556	3.189	< 0.001	< 0.001
Heat shock protein family A (Hsp70) member 2	A0A8C0P9Q2	HSPA2	480355	848.222	6761.000	3.157	< 0.001	< 0.001
LIM and cysteine rich domains 1	A0A8I3RW10	LMCD1	476545	207.167	1601.167	3.028	< 0.001	< 0.001
Myosin light chain 6B	A0A8I3RUW5	MYL6	606992	262.667	1753.444	2.798	< 0.001	< 0.001
Ankyrin repeat domain 2	A0A8C0PJR4	ANKRD2	486822	300.722	2191.111	2.768	< 0.001	< 0.001
Collagen type VI alpha 3 chain	A0A8C0P5R9	COL6A3	403582	212.333	631.278	2.748	< 0.001	< 0.001
Tubulin alpha chain	A0A8C0MZ87	TUBA4A	478918	1032.611	6596.722	2.670	< 0.001	< 0.001
Tubulin beta chain	A0A8I3MY05	TUBB	474830	581.222	3489.167	2.640	< 0.001	< 0.001
Peroxiredoxin-1	A0A8I3PG86	PRDX1	475375	1018.278	6070.389	2.559	< 0.001	< 0.001
ATP-dependent 6-phosphofructokinase	A0A8I3P7P5	PFKL	487797	39.333	146.833	2.552	< 0.001	< 0.001

SHSP domain-containing protein	A0A8I3MEJ0	HSPB2	611362	114.722	300.111	2.439	< 0.001	< 0.001
Adenylate kinase isoenzyme 1	A0A8C0SVP6	AK1	480712	360.667	1907.833	2.424	< 0.001	< 0.001
Collagen type VI alpha 1 chain	A0A8I3S7Z3	COL6A1	403668	219.056	705.722	2.423	< 0.001	< 0.001
Myozenin 2 OS=Canis lupus familiaris	A0A8I3Q871	MYOZ2	478524	414.389	1934.611	2.370	< 0.001	< 0.001
Heat shock protein beta-1	A0A8C0MMM4	HSPB1	403979	773.222	4103.111	2.333	< 0.001	< 0.001
Heat shock protein 90 alpha family class B member 1	A0A8C0S8X9	HSP90AB1	474919	558.056	2629.500	2.324	< 0.001	< 0.001
Glyceraldehyde-3-phosphate dehydrogenase (phosphorylating)	A0A8C0T2F6	LOC119877479	119877479	3685.944	15063.944	2.209	< 0.001	< 0.001
Heat shock protein 90 alpha family class A member 1	A0A8C0M536	HSP90AA1	480438	175.167	744.889	2.195	< 0.001	< 0.001
Transitional endoplasmic reticulum ATPase	A0A8I3MWF5	VCP	481590	222.833	930.056	2.154	< 0.001	< 0.001
Tripartite motif-containing protein 72	A0A8C0S8N8	TRIM72	489927	1581.556	6161.500	2.141	< 0.001	< 0.001
Elongation factor 1-alpha	A0A8C0TTJ0	EEF1A2	612521	2809.000	10767.722	2.140	< 0.001	< 0.001
Uncharacterized protein	A0A8C0PB66			1897.611	7019.556	2.092	< 0.001	< 0.001
Peptidylprolyl isomerase	A0A8I3MMG4	FKBP3	480306	107.444	379.333	1.977	< 0.001	< 0.001
Eukaryotic translation elongation factor 2	A0A8I3RV50	EEF2	476744	59.944	260.833	1.956	< 0.001	< 0.001
Kelch like family member 40	A0A8C0NDE6	KLHL40	485616	590.778	2136.333	1.896	< 0.001	< 0.001
Annexin	A0A8C0Z1Y0	ANXA1		630.500	1967.222	1.806	< 0.001	< 0.001

Four and a half LIM domains 1	A0A8C0NME4	FHL1	492162	3848.889	11786.722	1.721	< 0.001	< 0.001
Heterogeneous nuclear ribonucleoprotein D like	A0A8C0NIB3	HNRNPDL	476976	59.722	162.111	1.694	< 0.001	< 0.001
Heat shock protein family A (Hsp70) member 8	A0A8C0SFV4	HSPA8	479406	5859.167	16522.111	1.690	< 0.001	< 0.001
Voltage-dependent anion-selective channel protein 1	A0A8C0N523	VDAC1	474681	303.222	890.722	1.640	< 0.001	< 0.001
Fructose-bisphosphate aldolase	A0A8C0Z329	ALDOC	480622	208.944	527.556	1.634	< 0.001	< 0.001
Heat shock protein family B (small) member 6	A0A8C0THZ2	HSPB6	484574	495.278	1453.722	1.622	0.002	0.004
Tripartite motif containing 13	A0A8C0RNH1	TRIM13	112678926	124.389	326.611	1.607	< 0.001	< 0.001
Pyruvate kinase	A0A8C0NP15	PARP6	478358	1311.944	3389.556	1.594	< 0.001	< 0.001
Vimentin	A0A8C0N8E3	VIM	477991	643.722	1905.444	1.557	< 0.001	< 0.001
Carbonic anhydrase	A0A8I3P452	CA3	487032	3574.944	8805.333	1.550	< 0.001	< 0.001
Phosphoglycerate kinase	A0A8C0Z0K4	PGK1	480964	1190.611	2856.111	1.527	< 0.001	< 0.001
Bridging integrator 1	A0A8I3S7B1	BIN1	483870	3439.000	8735.722	1.486	< 0.001	< 0.001
Tyrosine 3-monooxygenase/tryptophan 5-monooxygenase activation protein gamma	A0A8I3MT06	YWHAG	489818	655.000	1526.778	1.475	< 0.001	< 0.001
Lamin A/C	A0A8I3NEK0	LMNA	480124	1481.333	4030.389	1.467	< 0.001	< 0.001
Fructose-bisphosphate aldolase	A0A8C0R9Z8	ALDOA	112640048	10192.611	22737.778	1.442	< 0.001	< 0.001
Protein unc-45 homolog B	A0A8C0NYA3	UNC45B	480606	29.278	64.556	1.416	< 0.001	< 0.001
Bridging integrator 1	A0A8C0Q7P9			46.500	109.000	1.410	< 0.001	0.002

Protein-arginine deiminase	A0A8C0Q899			49.889	146.722	1.409	0.002	0.003
Apolipoprotein B mRNA editing enzyme catalytic subunit 2	A0A8C0MII3	APOBEC2	481788	712.722	1682.167	1.322	< 0.001	< 0.001
Creatine kinase	A0A8C0S5U1	CKM	476435	11214.111	23111.611	1.311	< 0.001	< 0.001
Heterogeneous nuclear ribonucleoproteins A2/B1	A0A8C0M9Q4	HNRNPA2B1	475260	338.833	708.500	1.256	< 0.001	< 0.001
Mono-ADP ribosylhydrolase 1	A0A8C0RGX8	MACROD1	612142	89.000	207.611	1.250	< 0.001	< 0.001
La ribonucleoprotein 1, translational regulator	A0A8I3MY08	LARP1	612929	682.333	1367.222	1.244	< 0.001	0.003
Uncharacterized protein	A0A8C0MRK1			583.000	815.167	1.209	0.046	0.049
Cysteine and glycine rich protein 3	A0A8I3NXE1	CSRP3	610946	740.556	1374.611	1.199	0.002	0.005
PDZ and LIM domain 3	A0A8C0SAM4	PDLIM3	482907	1589.278	3462.778	1.159	< 0.001	< 0.001
Protein kinase C and casein kinase substrate in neurons 3	A0A8C0PLA9	PACSIN3	475984	549.111	1116.444	1.132	< 0.001	< 0.001
Myosin light chain 3	A0A8I3NHZ9	MYL3	476644	3118.722	6236.000	1.124	0.002	0.005
Acetyl-CoA acetyltransferase 1	A0A8I3MQ37	ACAT1	489421	664.389	1036.167	1.026	0.012	0.016
Albumin	A0A8C0NV01	ALB	403550	1255.556	2016.667	1.018	0.002	0.005
SET and MYND domain containing 1	A0A8C0MJD1	SMYD1	475758	408.000	831.111	0.995	0.006	0.009
Semaphorin 4D	A0A8C0PKB4	SEMA4D	476350	431.944	799.444	0.977	< 0.001	0.002
Myotilin	A0A8C0SBP2	MYOT	100855693	2671.056	4828.889	0.966	< 0.001	< 0.001
Heparan sulfate proteoglycan 2	A0A8C0RAX9	HSPG2	403440	103.389	155.389	0.945	0.038	0.042
Sodium/potassium-transporting ATPase subunit beta	A0A8C0PQV9	ATP1B1	403966	352.944	656.611	0.944	< 0.001	< 0.001
Troponin I1, slow skeletal type	A0A8C0LX10	TNNI1	100685160	5456.722	9319.944	0.940	< 0.001	0.001
Keratin 9	A0A8C0P7U8	KRT9		2024.833	3622.667	0.911	< 0.001	0.003

Myosin light chain 2	A0A8I3Q4A5	MYL2	403614	8492.056	13456.222	0.909	0.001	0.003
Elongation factor Tu	A0A8C0PZN9	TUFM	479796	505.611	903.722	0.879	< 0.001	< 0.001
Calsequestrin	A0A8C0SA33	CASQ2	483134	954.389	1944.667	0.846	0.003	0.005
Potassium voltage-gated channel subfamily H member 3	A0A8C0NZI8	KCNH3	112678293	676.944	1141.389	0.841	0.011	0.014
Medium-chain specific acyl-CoA dehydrogenase, mitochondrial	A0A8I3RRX0	ACADM	490207	254.778	451.222	0.816	0.010	0.014
Thioredoxin domain-containing protein	A0A8C0SJW6			498.389	766.333	0.788	0.008	0.012
Creatine kinase	A0A8I3PNS0	CKMT2	479163	2988.833	4554.389	0.787	0.003	0.005
Caveolin	A0A8C0MMY6	CAV1	403980	903.556	1449.556	0.776	< 0.001	< 0.001
Kelch like family member 41	A0A8C0MH92	KLHL41	478784	5631.611	9276.944	0.773	< 0.001	< 0.001
Calcium-transporting ATPase	A0A8I3QPB8	ATP2C1	477066	450.278	678.278	0.769	< 0.001	0.003
Collagen type IV alpha 1 chain	A0A8I3PA57	COL4A1	403496	140.389	170.111	0.768	0.030	0.034
Carnitine O-acetyltransferase	A0A8I3PPI0	CRAT	491304	610.833	947.722	0.723	0.003	0.006
SH3 and multiple ankyrin repeat domains 1	A0A8I3NPZ0	SHANK1	484359	1340.889	2097.000	0.720	0.006	0.010
Filamin C	A0A8C0SKH6	FLNC	482266	8080.778	12045.500	0.704	0.034	0.038
78 kDa glucose-regulated protein	A0A8C0MIT4	HSPA5	480726	1164.056	1803.222	0.700	0.004	0.007
Alpha-1,4 glucan phosphorylase	A0A8C0RCX9	PYGM	611078	18560.722	25991.167	0.649	0.001	0.003
Histone H2B	A0A8C0TVM6	H2BC1	488252	3901.167	5491.167	0.626	0.003	0.005
GLOBIN domain-containing protein	A0A8C0MKR1			903.778	1218.167	0.619	0.024	0.029
Citrate synthase	A0A8I3N6V8	CS	474403	2660.111	3735.333	0.614	0.003	0.006
Keratin, type II cytoskeletal 1	A0A8C0TQZ9	KRT1	444857	1257.500	1917.111	0.604	0.034	0.038
Family with sequence similarity 53 member B	A0A8C0TAC6	FAM53B	611311	325.000	528.000	0.589	0.030	0.034
Sarcoglycan alpha	A0A8I3NCD7	SGCA	609265	178.056	249.778	0.567	0.008	0.012
PDZ and LIM domain 5	A0A8I3PE25	PDLIM5	478482	3615.778	4623.722	0.519	0.026	0.031

Four and a half LIM domains 3	A0A8I3QAY6	FHL3	608080	639.389	852.778	0.502	0.047	0.049
NADH-cytochrome b5 reductase	A0A8C0M4J8	CYB5R1	606823	150.722	209.389	0.460	0.011	0.014
**Downregulated in XLMTM (WT)**								
Atypical kinase COQ8A, mitochondrial	A0A8C0S931	COQ8A	480108	1411.611	563.667	-1.303	< 0.001	< 0.001
RNA pseudouridine synthase D4	A0A8C0LUJ0	RPUSD4	489295	283.278	119.722	-1.109	0.003	0.006
Acetyltransferase component of pyruvate dehydrogenase complex	A0A8C0NAN7	DLAT	489406	347.167	177.444	-1.075	0.027	0.032
Pyruvate dehydrogenase E1 component subunit beta	A0A8C0RNN4	PDHB	476574	2308.722	1074.500	-1.015	< 0.001	< 0.001
Pyruvate dehydrogenase E1 component subunit alpha	A0A8C0T0M1	PDHA1	119868382	3438.667	1623.000	-1.011	< 0.001	< 0.001
Uncharacterized protein	A0A8C0TKG5			9911.722	4977.556	-0.987	< 0.001	< 0.001
Creatine kinase	A0A8I3MET3			962.833	428.500	-0.968	0.005	0.008
Myosin-8	A0A8C0LVV3	MYH8	489503	697.167	337.056	-0.959	0.018	0.023
NADH dehydrogenase [ubiquinone] flavoprotein 1, mitochondrial	A0A8I3N636	NDUFV1	476004	1197.111	571.722	-0.933	0.010	0.013
Tropomyosin 1	A0A8C0MHC6	TPM1	478332	6269.167	3331.333	-0.915	< 0.001	< 0.001
NADH dehydrogenase [ubiquinone] iron-sulfur protein 7, mitochondrial	A0A8C0QGW9	NDUFS7	476754	573.889	282.889	-0.901	< 0.001	< 0.001
NADH dehydrogenase [ubiquinone] iron-sulfur protein 3, mitochondrial	A0A8C0SSI5	NDUFS3	475978	1832.222	890.944	-0.900	< 0.001	< 0.001
Myosin binding protein C, fast type	A0A8C0YW53	MYBPC2	476404	2466.944	1199.056	-0.894	0.009	0.013
Proton-translocating NAD(P)(+) transhydrogenase	A0A8C0S4T3	NNT	479342	2617.167	1377.944	-0.862	< 0.001	< 0.001
Ubiquitin like modifier activating enzyme 6	A0A8I3N8D3	UBA6	475160	2379.889	1117.722	-0.859	0.012	0.016
Long-chain-fatty-acid--CoA ligase	A0A8C0YXC2			1252.778	687.278	-0.858	0.013	0.017
Myosin-2	A0A8C0T2B0	MYH2	608242	89223.111	45629.500	-0.803	0.024	0.029

Myosin-8	A0A8I3MIP2	MYH8	403808	4269.167	2333.389	-0.795	0.036	0.039
Cytochrome b-c1 complex subunit Rieske, mitochondrial	A0A8I3MI67	UQCRFS1	476503	2596.278	1357.389	-0.778	0.003	0.006
Solute carrier family 25 member 12	A0A8C0TZ22	SLC25A12	478798	2900.278	1600.389	-0.756	0.005	0.009
Troponin I2, fast skeletal type	A0A8C0TUY8	TNNI2	475995	6126.056	3397.056	-0.744	< 0.001	0.003
Synaptophysin like 2	A0A8C0N379	SYPL2	611827	1054.722	587.278	-0.729	< 0.001	0.001
ATP synthase subunit alpha	A0A8C0QKG4	ATP5F1A	480149	29519.889	16818.556	-0.713	< 0.001	< 0.001
Calcium-transporting ATPase	A0A8I3MN74	ATP2A1	479797	26915.444	14753.667	-0.708	0.001	0.003
Dihydrolipoyllysine-residue succinyltransferase component of 2-oxoglutarate dehydrogenase complex, mitochondrial	A0A8C0NT07			1044.500	643.222	-0.643	0.004	0.006
ATP synthase subunit O, mitochondrial	A0A8I3PXG0	ATP5PO	478410	1382.778	815.944	-0.627	0.010	0.014
Ubiquinol-cytochrome c reductase core protein 2	A0A8C0P0Z5	UQCRC2	479815	2966.833	1797.778	-0.614	0.001	0.003
ATP synthase subunit beta	A0A8I3NCH2	ATP5F1B	403669	15358.444	9273.389	-0.607	0.002	0.005
Helicase with zinc finger	A0A8I3PSR6	HELZ	490907	1557.500	987.556	-0.607	0.007	0.011
Myosin light chain, phosphorylatable, fast skeletal muscle	A0A8C0SLJ7	MYLPF	479772	17330.056	11274.167	-0.607	0.012	0.016
NDUFA4 mitochondrial complex associated	A0A8C0NZR2	NDUFA4	100856334	3360.167	2047.556	-0.583	0.004	0.007
Calsequestrin	A0A8C0NG78			7128.444	4613.278	-0.580	0.005	0.009
Actin alpha 1, skeletal muscle	A0A8C0N2I3	ACTA1	488984	45303.222	29954.722	-0.580	< 0.001	< 0.001
Aspartate aminotransferase	A0A8C0LW07	GOT2	478103	3553.167	2198.278	-0.564	0.018	0.022
Cytochrome c oxidase subunit 2	A0A172R529	COX2	804479	1422.611	854.333	-0.563	0.022	0.027
MICOS complex subunit MIC60	A0A8I3RXK0	IMMT	475764	2943.056	1942.833	-0.517	0.010	0.014
Ubiquitin protein ligase E3 component n-recognin 4	A0A8C0MQV8	UBR4	478211	1728.278	1193.667	-0.504	0.028	0.033
Phosphopyruvate hydratase	A0A8I3PWZ2	ENO1	479597	3933.333	2517.333	-0.496	0.031	0.035
Ubiquinol-cytochrome c reductase core protein 1	A0A8I3QCC4	UQCRC1	608455	5498.556	3598.944	-0.492	0.015	0.019

Acyl-CoA dehydrogenase very long chain	A0A8I3P5V9	ACADVL	489463	1919.278	1272.167	-0.484	0.044	0.047
ATP synthase subunit gamma	A0A8C0LQA1	ATP5F1C	478009	2473.444	1670.500	-0.467	0.025	0.030
Uncharacterized protein	A0A8C0T8P6			192.889	127.722	-0.460	0.016	0.020
Myosin light chain 1	A0A8I3P3S6	MYL1	478896	7622.500	5524.778	-0.443	0.035	0.038
Transmembrane serine protease 13	A0A8I3MN50	TMPRSS13	610827	1087.111	718.444	-0.442	0.038	0.042
Tropomyosin 2	A0A8C0N098	TPM2	481598	28946.667	22250.889	-0.418	0.013	0.017
Sarcalumenin	A0A8C0SNB7	SRL	490030	6991.000	4937.722	-0.396	0.020	0.025
Actinin alpha 2	A0A8C0MXP0	ACTN2	479191	32734.000	24701.889	-0.390	0.006	0.009
**No significant differences between WT and XLMTM**								
Malate dehydrogenase	A0A8C0Q1T7	MDH1		248.222	486.778	1.190	0.077	0.077
Xin actin binding repeat containing 1	A0A8I3QSZ5	XIRP1		186.611	315.000	0.811	0.066	0.067
Nephrocystin 3	A0A8C0Z478	NPHP3		181.556	356.000	0.799	0.077	0.077
Desmoplakin	A0A8I3P212	DSP		210.056	313.000	0.660	0.084	0.081
L-lactate dehydrogenase	A0A8C0PCN9	LDHB		2285.500	3134.944	0.660	0.065	0.067
Elongation factor 1-gamma	A0A8I3NCW4	TUT1		989.444	1280.167	0.650	0.077	0.076
Sodium/potassium-transporting ATPase subunit alpha	A0A8C0NKC6	ATP1A4		202.000	312.500	0.641	0.110	0.108
Junction plakoglobin	A0A8I3NL10	JUP		198.444	291.111	0.606	0.055	0.056
Keratin 18	A0A8I3PJ84	KRT18		996.889	1546.889	0.589	0.156	0.152
Triosephosphate isomerase	A0A8I3PRM7	TPI1		1775.556	2215.278	0.576	0.141	0.138
Myosin-4	A0A8C0M1G3	MYH4		2000.389	2261.056	0.543	0.272	0.262
Tropomyosin 4 delta	A0A0N9JE84	Tpm4		1147.222	1414.222	0.517	0.179	0.174
PDZ and LIM domain 5	A0A8C0PEX2	PDLIM5		3111.889	4029.667	0.507	0.086	0.083
Golgin A3	A0A8I3S9G9	GOLGA3		977.111	1472.833	0.505	0.076	0.076
EH domain containing 2	A0A8I3MKL5	EHD2		128.167	160.944	0.493	0.223	0.213
Uncharacterized protein	A0A8I3Q055	PRKX		86.056	107.500	0.483	0.078	0.076
Heat shock protein family B (small) member 7	A0A8C0RWS8	HSPB7		121.722	157.333	0.471	0.192	0.186
Synaptopodin 2 OS=Canis lupus familiaris	A0A8I3QWM0	SYNP		395.889	519.389	0.462	0.081	0.080

Stress-70 protein, mitochondrial	A0A8C0SP21	HSPA9	778.389	967.611	0.421	0.066	0.067
60 kDa heat shock protein, mitochondrial	A0A8C0TC09	HSPD1	1455.667	1836.556	0.410	0.157	0.152
Malate dehydrogenase	A0A8C0TCL8		3968.778	4866.111	0.400	0.060	0.062
Histone H4	F2Z4N2	H4C9	2531.778	2998.167	0.370	0.077	0.076
H1.0 linker histone	A0A8I3RUA8	H1-0	502.944	564.222	0.337	0.199	0.192
Histone H3	A0A8C0LQ69	LOC475916	3341.667	4019.944	0.337	0.053	0.055
Tropomyosin 3	A0A8I3S1D8	TPM3	14271.333	16020.278	0.324	0.158	0.152
Chromosome 2 open reading frame 72	A0A8I3PEA8	C2orf72	81.444	92.722	0.302	0.267	0.257
Succinate dehydrogenase [ubiquinone] iron-sulfur subunit, mitochondrial	A0A8C0RFS0	SDHB	1020.000	1157.722	0.287	0.233	0.223
Keratin 15	A0A8I3NXE8	KRT15	1019.222	1017.667	0.287	0.323	0.311
Phosphopyruvate hydratase	A0A8I3N5K2	ENO3	12130.611	12740.111	0.272	0.280	0.270
Actinin alpha 3	A0A8I3NN24	ACTN3	4676.056	4996.389	0.260	0.254	0.246
Uncharacterized protein	A0A8I3PTP5	ECHS1	122.778	141.333	0.259	0.211	0.203
Isocitrate dehydrogenase (NADP(+))	A0A8C0PSM3	IDH1	7104.333	7883.167	0.255	0.185	0.178
Tropomodulin 4	A0A8I3PE86	TMOD4	761.778	844.944	0.226	0.415	0.401
Keratin, type II cytoskeletal 1	A0A8C0P4P0	KRT1	8134.833	9840.111	0.222	0.291	0.277
Myosin-7	A0A8I3MJC8	MYH7	41239.667	40326.667	0.217	0.504	0.483
Dysferlin OS=Canis lupus familiaris	A0A8I3P9A7	LOC491973	171.389	162.778	0.189	0.625	0.596
Reticulon	A0A8I3NRF3	RTN4	626.667	655.167	0.187	0.537	0.512
Glycosylphosphatidylinositol anchor attachment 1	A0A8I3N3K0	GPAA1	1018.222	1018.111	0.181	0.610	0.585
PRA1 family protein	A0A8I3NDE1	ARL6IP5	838.444	843.500	0.173	0.466	0.450
EF-hand domain-containing protein	A0A8C0SD34	SELENON	213.667	215.944	0.157	0.488	0.470
Apoptosis inducing factor mitochondria associated 1	A0A8C0TDQ6	AIFM1	415.944	446.500	0.148	0.482	0.466
LIM domain binding 3	A0A8C0M2K9	LDB3	5315.333	5593.056	0.129	0.501	0.482
Cardiac titin (Fragment)	Q7YRF5	TTN	9726.889	4576.444	0.111	0.830	0.828
Succinate dehydrogenase [ubiquinone] flavoprotein subunit, mitochondrial	A0A8I3P4H0	SDHA	2642.056	2692.889	0.103	0.506	0.481

Nebulin	A0A8I3N6H5	NEB	14190.33<br>3	7548.222	0.048	0.920	0.914
Glycogenin 1	A0A8I3PGI9	GYG1	422.444	415.667	0.015	0.950	0.943
Voltage-dependent anion-selective channel protein 2	A0A8C0P0B2	VDAC2	3820.833	3522.722	0.002	0.993	0.992
Leucine rich repeat and coiled-coil centrosomal protein 1	A0A8C0TD23	LRRCC1	293.500	247.056	-0.025	0.927	0.919
Calreticulin	A0A8C0P5H0	CALR	342.111	317.222	-0.028	0.917	0.915
Keratin, type II cytoskeletal 2 epidermal	A0A8C0RQ12	KRT2	5211.833	5212.556	-0.036	0.917	0.919
Cytochrome c1	A0A8C0RTK9	CYC1	1027.556	897.056	-0.048	0.823	0.823
Epoxide hydrolase 1	A0A8C0SEZ0	EPHX1	277.389	252.722	-0.050	0.842	0.839
Fumarate hydratase, mitochondrial	A0A8C0Q033	FH	495.000	443.944	-0.063	0.915	0.920
Keratin 75	A0A8I3PNP3	KRT6A	458.444	377.722	-0.075	0.863	0.860
Keratin, type I cytoskeletal 10	A0A8I3NL87	KRT10	10378.88<br>9	10249.11<br>1	-0.105	0.683	0.659
Dihydrolipoyl dehydrogenase	A0A8I3PTV9	DLD	2603.778	2188.556	-0.119	0.556	0.528
Nipsnap homolog 2	A0A8I3NCW1	NIPSNAP2	4564.167	3842.500	-0.139	0.456	0.441
Calsarcin 2	Q1AG03	MYOZ1	3080.000	2660.444	-0.151	0.528	0.503
Calcium-transporting ATPase	B6CAN1	ATP2A2	8057.333	6425.667	-0.159	0.480	0.465
IF rod domain-containing protein	A0A8C0SYM7	KRT18	2373.444	2276.278	-0.162	0.611	0.584
Oxoglutarate dehydrogenase (succinyl-transferring)	A0A8I3NKB5	OGDH	1042.056	875.333	-0.171	0.363	0.351
Superoxide dismutase [Mn], mitochondrial	A0A8C0Z4V9	SOD2	731.222	622.833	-0.172	0.408	0.395
Capping actin protein of muscle Z-line subunit alpha 2	A0A8C0SW56	CAPZA2	227.222	198.722	-0.172	0.156	0.152
Voltage-dependent anion-selective channel protein 3	A0A8C0Q760	VDAC3	2569.167	2146.167	-0.188	0.373	0.358
Amine oxidase	A0A8I3PYC1	AOC1	83.111	64.833	-0.192	0.341	0.328
Myosin-1	A0A8I3MN85	MYH1	4790.667	3603.389	-0.231	0.541	0.514
Solute carrier family 25 member 11	A0A8C0P6K8	SLC25A11	853.722	683.500	-0.238	0.335	0.323
Aconitate hydratase, mitochondrial	A0A8I3NYC8	ACO2	1747.778	1398.444	-0.241	0.453	0.440
ADP/ATP translocase	A0A8C0NE13	SLC25A4	8731.556	7040.556	-0.263	0.129	0.126
Uncharacterized protein	A0A8C0PAT5		735.278	273.167	-0.290	0.654	0.628
Cytochrome b-c1 complex subunit 8	A0A8I3NJC6	UQCRQ	873.167	637.056	-0.294	0.281	0.269
Uncharacterized protein	A0A8I3Q2S8	HADHB	2596.556	1972.833	-0.302	0.151	0.147
Phosphate carrier protein, mitochondrial	A0A8I3Q073	SLC25A3	2940.278	2219.167	-0.321	0.143	0.139

Uncharacterized protein	A0A8C0T6T0		406.667	145.556	-0.337	0.594	0.569
Myosin binding protein C, slow type	A0A8C0SR77	MYBPC1	27574.500	21386.611	-0.345	0.167	0.161
Voltage-dependent anion-selective channel protein 1	A0A8C0QBR5	VDAC1	11948.444	8343.667	-0.355	0.118	0.115
Drebrin 1	A0A8C0M2Y5		104.889	79.667	-0.377	0.283	0.271
Cytochrome c oxidase subunit 5A, mitochondrial	A0A8C0MTN1	COX5A	263.833	177.056	-0.379	0.423	0.407
Enoyl-CoA hydratase	A0A8C0QB72	ECHS1	3368.222	2574.667	-0.392	0.049	0.052
Ubiquinone biosynthesis monooxygenase COQ6, mitochondrial	A0A8I3NFA6	COQ6	445.167	317.056	-0.409	0.123	0.121
Cytochrome c oxidase subunit 6C	F1PKU9		1470.056	957.167	-0.419	0.106	0.104
Troponin C2, fast skeletal type	A0A8I3NNU2	TNNC2	1170.500	818.111	-0.425	0.422	0.408
Troponin T, fast skeletal muscle	A0A8C0Z6J3	TNNT3	11662.833	7576.222	-0.430	0.061	0.063
REC8 meiotic recombination protein	A0A8C0NDC6	REC8	1314.722	915.556	-0.430	0.107	0.104
Glycogen [starch] synthase	A0A8I3MKP1	GYS1	336.778	243.500	-0.456	0.049	0.052
Uncharacterized protein	A0A8C0P4P8		1329.611	493.556	-0.467	0.505	0.482
Titin	A0A8I3S7V6	TTN	52294.111	18427.778	-0.518	0.370	0.357
ATP synthase subunit b	A0A8I3MFN6	LOC119872230	1135.889	700.444	-0.523	0.260	0.252
Alpha-cardiac myosin heavy chain (Fragment)	Q076A1	MYH1	562.167	347.444	-0.560	0.140	0.138
Prohibitin	A0A8I3PW80	PHB2	664.889	463.500	-0.569	0.134	0.131
Ryanodine receptor 1	A0A8C0SJC2	RYR1	344.278	200.944	-0.658	0.216	0.208
Protein disulfide-isomerase	A0A8C0RUF5		426.500	259.944	-0.696	0.219	0.210
Myosin phosphatase Rho interacting protein	A0A8C0M1X4	MPRIP	152.500	62.278	-0.795	0.062	0.063

Myomesin 1	A0A8C0Q7V0	MYOM1	986.500	409.833	-0.845	0.205	0.198

Proteins excluded from downstream analysis following filtering for >1 unique peptide

GLOBIN domain-containing protein			269.333	6382.333	4.564		
Glyceraldehyde-3-phosphate dehydrogenase			123.944	1839.111	3.878		
Decorin		DCN	707.611	6179.944	3.038		
UTP--glucose-1-phosphate uridylyltransferase			308.056	2310.000	2.900		
ATP-dependent 6-phosphofructokinase			479.167	3140.167	2.840		
Annexin			205.222	1144.833	2.543		
Tubulin beta chain			90.778	438.389	2.391		
IRF tryptophan pentad repeat domain-containing protein			409.611	817.778	1.288		
Muscle-restricted coiled-coil protein			135.444	287.167	1.184		
Glutathione peroxidase			205.278	388.222	1.135		
Tyrosine 3-monooxygenase/tryptophan 5-monooxygenase activation protein epsilon		YWHAE	184.778	381.889	1.065		
Protein phosphatase 6 regulatory subunit 1		PPP6R1	6612.333	9648.944	0.792		
Histone H2A			1102.611	1653.778	0.695		
Keratin, type II cytoskeletal 1			4811.333	7691.278	0.594		
Ubiquitin B			2014.222	2974.722	0.568		
Keratin 15			3555.722	4110.778	0.377		
Malate dehydrogenase, mitochondrial			1058.833	1272.222	0.354		
Ubiquinone biosynthesis protein			280.056	324.000	0.097		
Desmin		DES	7888.944	7705.167	0.075		
Galectin 4		LGALS4	981.722	895.722	-0.024		
ATP synthase subunit d, mitochondrial			411.056	279.833	-0.408		
Poly [ADP-ribose] polymerase			801.667	586.778	-0.437		

Prohibitin		798.000	561.278	-0.438
Myosin light chain 4	MYL4	248.889	160.667	-0.537
Myomesin 2	MYOM2	2035.056	1304.722	-0.582
Cyclic AMP-responsive element-binding protein 5	CREB5	337.444	204.000	-0.725
SAMM50 sorting and assembly machinery component	SAMM50	168.667	91.667	-0.863
Bacteriophage Mu transposase		642.889	244.778	-0.983
NADH-ubiquinone oxidoreductase 75 kDa subunit, mitochondrial		1444.667	639.667	-1.059
Aspartate ammonia-lyase		619.000	127.611	-1.396
Transmembrane anterior posterior transformation 1	TAPT1	879.389	350.000	-1.481
SRC kinase signaling inhibitor 1	SRCIN1	315.944	105.444	-1.586

Supplemental Table 3: Mouse global untargeted proteomics analysis where WT are compared to Mtm1$^{y/-}$ tissue.

protein_identifier	log2FC	padj	direction
Slco5a1	-4.4971556	0.00980885	Mtm1y/-_DOWN
Myom1	-3.4220411	5.35E-05	Mtm1y/-_DOWN
Nt5c1a	-3.3825882	0.01266985	Mtm1y/-_DOWN
Bdh1	-2.8579899	0.00098519	Mtm1y/-_DOWN
Cdk5rap1	-2.8137621	0.03747051	Mtm1y/-_DOWN
Myl2	-2.7990391	0.0002145	Mtm1y/-_DOWN
Zfp174	-2.7457755	0.00076649	Mtm1y/-_DOWN
Myl2	-2.6580965	0.00083054	Mtm1y/-_DOWN
Neb	-2.6343166	0.00631841	Mtm1y/-_DOWN
Acot11	-2.3699961	0.01070932	Mtm1y/-_DOWN
Syp	-2.1520762	0.00438691	Mtm1y/-_DOWN
Gm5965	-2.0891687	0.03161721	Mtm1y/-_DOWN
Myh7	-2.0158427	0.00261359	Mtm1y/-_DOWN
Smc1a	-2.0121742	0.00124746	Mtm1y/-_DOWN
Csnk1g3	-1.9415694	0.00648976	Mtm1y/-_DOWN
Tnnt1	-1.9281731	0.01347128	Mtm1y/-_DOWN
Tnni1	-1.8926769	0.01502101	Mtm1y/-_DOWN
Tnnc1	-1.8511421	0.03517873	Mtm1y/-_DOWN
Lsmem1	-1.8392715	0.0012277	Mtm1y/-_DOWN
Myh6	-1.8005634	0.00258289	Mtm1y/-_DOWN
Plin3	-1.7550238	0.02677832	Mtm1y/-_DOWN
Mtm1	-1.7476727	0.0190986	Mtm1y/-_DOWN
Atp2a2	-1.6974457	0.00754365	Mtm1y/-_DOWN
Grb14	-1.6254353	0.00188855	Mtm1y/-_DOWN
Myl3	-1.6048301	0.00060035	Mtm1y/-_DOWN
Ldb3	-1.6016961	0.00936449	Mtm1y/-_DOWN
Plin5	-1.5310877	0.03275795	Mtm1y/-_DOWN
Tpm3	-1.51635	0.00563316	Mtm1y/-_DOWN
Myl3	-1.4532971	0.00941481	Mtm1y/-_DOWN

Cct6b	-1.4438694	0.01585332	Mtm1y/-_DOWN
Slc7a8	-1.42483	0.04878888	Mtm1y/-_DOWN
Aqp7	-1.3639355	0.03642137	Mtm1y/-_DOWN
Dhrs7c	-1.3466438	0.00746357	Mtm1y/-_DOWN
Myoz2	-1.3281864	0.00703313	Mtm1y/-_DOWN
Klhl34	-1.3189392	0.00179896	Mtm1y/-_DOWN
Phkg1	-1.2977696	0.00266057	Mtm1y/-_DOWN
Lpl	-1.288851	0.00307235	Mtm1y/-_DOWN
Rilpl1	-1.2683185	0.04789589	Mtm1y/-_DOWN
Lgals8	-1.2620488	0.03017859	Mtm1y/-_DOWN
Ank1	-1.2438215	0.00983719	Mtm1y/-_DOWN
Map2k6	-1.2381652	0.00031804	Mtm1y/-_DOWN
Slc25a31	-1.2275851	0.02644254	Mtm1y/-_DOWN
Mtfp1	-1.2193863	0.03109448	Mtm1y/-_DOWN
Xdh	-1.210088	0.02436459	Mtm1y/-_DOWN
Prxl2a	-1.2079959	0.00150238	Mtm1y/-_DOWN
Mgst1	-1.1987734	0.01282	Mtm1y/-_DOWN
Stac3	-1.1846555	0.00188855	Mtm1y/-_DOWN
Scn4b	-1.1819339	0.02759232	Mtm1y/-_DOWN
Slc16a1	-1.1774143	0.00398026	Mtm1y/-_DOWN
Atp2a2	-1.1336931	0.00824063	Mtm1y/-_DOWN
Phkb	-1.1332015	0.00667589	Mtm1y/-_DOWN
Myh15	-1.1122945	0.00474145	Mtm1y/-_DOWN
Tcap	-1.1013206	0.02519805	Mtm1y/-_DOWN
Kcnma1	-1.0988591	0.02801474	Mtm1y/-_DOWN
Col6a6	-1.0983418	0.02552206	Mtm1y/-_DOWN
Lsmem2	-1.0894271	0.0075836	Mtm1y/-_DOWN
Prodh	-1.0769179	0.00188855	Mtm1y/-_DOWN
Actn2	-1.0637551	0.01107467	Mtm1y/-_DOWN
Lrrc38	-1.0594082	0.04881921	Mtm1y/-_DOWN
Pdzd7	-1.058359	0.01241385	Mtm1y/-_DOWN
Ca4	-1.0508103	0.04285161	Mtm1y/-_DOWN

Oplah	-1.0467629	0.0050427	Mtm1y/-_DOWN
Wdr37	-1.0144477	0.00987415	Mtm1y/-_DOWN
Maob	-1.0096578	0.01952776	Mtm1y/-_DOWN
Mavs	-0.9963139	0.00565348	Mtm1y/-_DOWN
Smim14	-0.9905263	0.00140564	Mtm1y/-_DOWN
Bsg	-0.9790118	0.00624149	Mtm1y/-_DOWN
Ampd1	-0.9544666	0.01652729	Mtm1y/-_DOWN
Alpl	-0.9524957	0.01761818	Mtm1y/-_DOWN
Fkbp11	-0.9440608	0.00085218	Mtm1y/-_DOWN
Actg2	-0.9288642	0.00444848	Mtm1y/-_DOWN
Slc41a3	-0.9259535	0.01628312	Mtm1y/-_DOWN
Kcna7	-0.9241852	0.02325362	Mtm1y/-_DOWN
Phka1	-0.9147551	0.00424525	Mtm1y/-_DOWN
Fn3k	-0.9059406	0.01516973	Mtm1y/-_DOWN
Cpt1b	-0.890843	0.03161721	Mtm1y/-_DOWN
Myl6b	-0.869989	0.03453911	Mtm1y/-_DOWN
Dglucy	-0.8475622	0.02519805	Mtm1y/-_DOWN
Tinagl1	-0.8439794	0.0434862	Mtm1y/-_DOWN
Mief2	-0.8410208	0.00245829	Mtm1y/-_DOWN
Nnt	-0.8326608	0.01512894	Mtm1y/-_DOWN
Cd300lg	-0.8312631	0.02065652	Mtm1y/-_DOWN
Rap1gds1	-0.829861	0.00664888	Mtm1y/-_DOWN
Capza2	-0.8275198	0.00337862	Mtm1y/-_DOWN
Ldhb	-0.8271974	0.00230344	Mtm1y/-_DOWN
Slc9a2	-0.8216618	0.0479298	Mtm1y/-_DOWN
Ugp2	-0.8198993	0.02193987	Mtm1y/-_DOWN
Cacna1s	-0.8191976	0.01107467	Mtm1y/-_DOWN
Slc27a1	-0.8069292	0.00802581	Mtm1y/-_DOWN
Palm	-0.8054752	0.02233243	Mtm1y/-_DOWN
Capzb	-0.8014722	0.00444537	Mtm1y/-_DOWN
Acsl1	-0.799577	0.00342091	Mtm1y/-_DOWN
Smpdl3b	-0.7961056	0.0208926	Mtm1y/-_DOWN

Gtdc1	-0.7950002	0.01321529	Mtm1y/-_DOWN
Fkbp4	-0.7871133	0.00596027	Mtm1y/-_DOWN
Gapvd1	-0.7664231	0.0127899	Mtm1y/-_DOWN
Uqcrb	-0.7662508	0.02175898	Mtm1y/-_DOWN
Usp15	-0.7634363	0.01072829	Mtm1y/-_DOWN
Elp2	-0.7621125	0.04680339	Mtm1y/-_DOWN
Cacna2d1	-0.7620838	0.00824063	Mtm1y/-_DOWN
Vwa1	-0.7568835	0.04496746	Mtm1y/-_DOWN
Dnm1l	-0.7486175	0.02807068	Mtm1y/-_DOWN
Pank4	-0.7484132	0.00378676	Mtm1y/-_DOWN
Gbe1	-0.7467047	0.00205117	Mtm1y/-_DOWN
Etfdh	-0.7417084	0.03902682	Mtm1y/-_DOWN
Ephx2	-0.7357365	0.02965228	Mtm1y/-_DOWN
Lrrc30	-0.7314226	0.00795739	Mtm1y/-_DOWN
Hspa12b	-0.7260181	0.03249497	Mtm1y/-_DOWN
Atp1b1	-0.7232071	0.00593809	Mtm1y/-_DOWN
Myorg	-0.7228583	0.02519805	Mtm1y/-_DOWN
Acad11	-0.7156507	0.02325362	Mtm1y/-_DOWN
Pdlim5	-0.7117954	0.03098856	Mtm1y/-_DOWN
Cyp2u1	-0.6990236	0.03588869	Mtm1y/-_DOWN
Ndufb7	-0.6758244	0.02807068	Mtm1y/-_DOWN
Mpc2	-0.669505	0.01435037	Mtm1y/-_DOWN
Ndufb1	-0.6564545	0.03423471	Mtm1y/-_DOWN
Cox5a	-0.6374559	0.02984739	Mtm1y/-_DOWN
Nceh1	-0.6371438	0.03590477	Mtm1y/-_DOWN
Actbl2	-0.6350979	0.03406678	Mtm1y/-_DOWN
Gsk3a	-0.6320213	0.02902819	Mtm1y/-_DOWN
2310061I04Rik	-0.6298578	0.03327076	Mtm1y/-_DOWN
Mdh1	-0.6206448	0.02393804	Mtm1y/-_DOWN
Cops2	-0.6204817	0.01154283	Mtm1y/-_DOWN
Ndufb9	-0.6140771	0.03042937	Mtm1y/-_DOWN
Pdlim5	-0.6137377	0.02319024	Mtm1y/-_DOWN

Slc25a4	-0.6056508	0.00898342	Mtm1y/-_DOWN
Ndufs7	-0.5890884	0.00575434	Mtm1y/-_DOWN
Ndufb6	-0.589003	0.02407467	Mtm1y/-_DOWN
Stat5b	-0.5861982	0.00872229	Mtm1y/-_DOWN
Cul5	-0.5858432	0.04089487	Mtm1y/-_DOWN
Atp5mk	-0.5779841	0.03988155	Mtm1y/-_DOWN
Capn3	-0.5757643	0.00691789	Mtm1y/-_DOWN
Meioc	-0.573237	0.03249497	Mtm1y/-_DOWN
Hint3	-0.5538198	0.0212068	Mtm1y/-_DOWN
Map2k3	-0.5519407	0.02242091	Mtm1y/-_DOWN
Alad	-0.5498577	0.04870637	Mtm1y/-_DOWN
Rnf123	-0.5485533	0.00188855	Mtm1y/-_DOWN
Capza1	-0.5412544	0.03429237	Mtm1y/-_DOWN
Apoc1	-0.5337019	0.0310512	Mtm1y/-_DOWN
Atp5pd	-0.5251265	0.03653964	Mtm1y/-_DOWN
Ehd1	-0.5241869	0.02519805	Mtm1y/-_DOWN
Mtx2	-0.5157799	0.02644254	Mtm1y/-_DOWN
Acta1	-0.5114012	0.02902819	Mtm1y/-_DOWN
Rps6ka5	-0.5081962	0.03251192	Mtm1y/-_DOWN
Ndufs8	-0.5046032	0.01155792	Mtm1y/-_DOWN
Napepld	-0.4933085	0.03653964	Mtm1y/-_DOWN
Farsb	-0.4904556	0.022668	Mtm1y/-_DOWN
Trabd	-0.488119	0.01792204	Mtm1y/-_DOWN
Ndufa5	-0.4862214	0.01241385	Mtm1y/-_DOWN
Ndufs3	-0.4754747	0.01233932	Mtm1y/-_DOWN
Tmem38a	-0.4682636	0.01072829	Mtm1y/-_DOWN
Cnksr1	-0.4657445	0.02065466	Mtm1y/-_DOWN
Blvra	-0.4612491	0.01187909	Mtm1y/-_DOWN
Ppm1b	-0.4557202	0.03687779	Mtm1y/-_DOWN
Ndufv2	-0.4552573	0.01952776	Mtm1y/-_DOWN
Mgst3	-0.4515841	0.00595728	Mtm1y/-_DOWN
Mpc1	-0.4414786	0.0468836	Mtm1y/-_DOWN

Mcf2l	-0.4391877	0.03041892	Mtm1y/-_DOWN
Cad	-0.4262587	0.02580807	Mtm1y/-_DOWN
Abcc9	-0.4171627	0.00677077	Mtm1y/-_DOWN
Ndufb8	-0.4022156	0.02738138	Mtm1y/-_DOWN
Ppp2r1a	-0.4000918	0.03098856	Mtm1y/-_DOWN
Ppp2r5d	-0.396389	0.02325362	Mtm1y/-_DOWN
Abcc1	-0.3948523	0.0364574	Mtm1y/-_DOWN
Adss1	-0.3819175	0.0440039	Mtm1y/-_DOWN
Prkg1	-0.3762541	0.02701048	Mtm1y/-_DOWN
Mylk2	-0.3624977	0.03098856	Mtm1y/-_DOWN
Chp1	-0.3617066	0.02519805	Mtm1y/-_DOWN
Ppp2r5a	-0.3017107	0.0440039	Mtm1y/-_DOWN
Mtarc2	-0.2930185	0.01301243	Mtm1y/-_DOWN
Pigs	0.29492002	0.03139357	Mtm1y/-_UP
Cdipt	0.29611137	0.02228774	Mtm1y/-_UP
Psap	0.29734089	0.02030661	Mtm1y/-_UP
Rhoa	0.29946299	0.01654574	Mtm1y/-_UP
Dhrs1	0.30871657	0.04201775	Mtm1y/-_UP
Hnrnpk	0.32148665	0.01525645	Mtm1y/-_UP
Lmf2	0.32244534	0.03307256	Mtm1y/-_UP
Ddost	0.32618764	0.04807866	Mtm1y/-_UP
Atp6v0a2	0.32648598	0.02012876	Mtm1y/-_UP
Foxred1	0.33667435	0.03271331	Mtm1y/-_UP
Gdi2	0.34294463	0.02984739	Mtm1y/-_UP
Alg14	0.34468796	0.02984739	Mtm1y/-_UP
Cutc	0.345878	0.02677832	Mtm1y/-_UP
Ganab	0.34619102	0.03421316	Mtm1y/-_UP
Ppp3r1	0.34679555	0.03990608	Mtm1y/-_UP
Lnpep	0.34754598	0.01969491	Mtm1y/-_UP
Mybbp1a	0.36162803	0.01792204	Mtm1y/-_UP
Hspa5	0.36494613	0.0471571	Mtm1y/-_UP
Dysf	0.37169503	0.04435749	Mtm1y/-_UP

Mtpap	0.3803257	0.03139357	Mtm1y/-_UP
Gpaa1	0.38362953	0.00294434	Mtm1y/-_UP
Pigk	0.38427461	0.02495705	Mtm1y/-_UP
Rpn2	0.38541327	0.04540771	Mtm1y/-_UP
Hnrnpl	0.3930309	0.04143961	Mtm1y/-_UP
Prkcsh	0.39351721	0.03057597	Mtm1y/-_UP
Ppa1	0.39684297	0.03944954	Mtm1y/-_UP
Cox15	0.40092403	0.02325362	Mtm1y/-_UP
Eif3b	0.41775781	0.01241385	Mtm1y/-_UP
Memo1	0.42245052	0.017533	Mtm1y/-_UP
Fn3krp	0.42309067	0.01233932	Mtm1y/-_UP
Prep	0.42466769	0.01585332	Mtm1y/-_UP
Snrpd3	0.43357677	0.01665419	Mtm1y/-_UP
Clcn3	0.43523132	0.03650526	Mtm1y/-_UP
Hnrnpu	0.43775181	0.04750106	Mtm1y/-_UP
Kng1	0.44023853	0.01932822	Mtm1y/-_UP
Aldh3a2	0.44113032	0.02519805	Mtm1y/-_UP
Rab2a	0.4415347	0.02228774	Mtm1y/-_UP
Eef2	0.44590195	0.01641653	Mtm1y/-_UP
Smurf1	0.45313662	0.04580151	Mtm1y/-_UP
Uggt1	0.45806582	0.01051887	Mtm1y/-_UP
Cyp20a1	0.45931111	0.03406678	Mtm1y/-_UP
Ube2n	0.46171696	0.04822213	Mtm1y/-_UP
Rab10	0.46227484	0.01321549	Mtm1y/-_UP
Tppp3	0.46451458	0.01975291	Mtm1y/-_UP
Gm20431	0.46512869	0.01385077	Mtm1y/-_UP
Apex1	0.46533707	0.02475125	Mtm1y/-_UP
Hnrnpul2	0.46790952	0.02152821	Mtm1y/-_UP
Lamp2	0.47336251	0.02325362	Mtm1y/-_UP
Afg3l1	0.47370509	0.00732615	Mtm1y/-_UP
Ncln	0.47421653	0.00517678	Mtm1y/-_UP
Ipo4	0.47452044	0.01753494	Mtm1y/-_UP

Lamp1	0.47480407	0.03307256	Mtm1y/-_UP
Tsn	0.47709071	0.02243489	Mtm1y/-_UP
Ldb3	0.4812227	0.03037666	Mtm1y/-_UP
Msra	0.48567136	0.03800795	Mtm1y/-_UP
Tmx1	0.48780331	0.04416551	Mtm1y/-_UP
Colgalt1	0.48980096	0.01031267	Mtm1y/-_UP
Tmed2	0.49066749	0.03012449	Mtm1y/-_UP
Ero1a	0.49095495	0.01540352	Mtm1y/-_UP
Esd	0.49503612	0.02910157	Mtm1y/-_UP
Gna13	0.4952995	0.04158156	Mtm1y/-_UP
Ociad1	0.49951225	0.01261577	Mtm1y/-_UP
Acsl3	0.50066346	0.0422763	Mtm1y/-_UP
Exoc6	0.50555961	0.04031388	Mtm1y/-_UP
Extl1	0.50849285	0.02950399	Mtm1y/-_UP
Slc35b1	0.50913953	0.02807068	Mtm1y/-_UP
Tmed9	0.51111563	0.00529698	Mtm1y/-_UP
Ap2a2	0.51551667	0.0360459	Mtm1y/-_UP
Slc12a4	0.51553672	0.04626722	Mtm1y/-_UP
Tmed4	0.51601589	0.00802581	Mtm1y/-_UP
Ube2l3	0.51677599	0.03653964	Mtm1y/-_UP
Ykt6	0.51972121	0.045226	Mtm1y/-_UP
Rab11b	0.52049743	0.01643177	Mtm1y/-_UP
Sec61b	0.52171563	0.0253275	Mtm1y/-_UP
Nudt21	0.52831697	0.01107467	Mtm1y/-_UP
Nomo1	0.52851182	0.00614265	Mtm1y/-_UP
Pcyox1	0.52992181	0.03042937	Mtm1y/-_UP
Ssr4	0.53127805	0.01765676	Mtm1y/-_UP
F2	0.53135788	0.01540352	Mtm1y/-_UP
Rcn1	0.53386427	0.03098856	Mtm1y/-_UP
Prkaa2	0.53572985	0.04158156	Mtm1y/-_UP
Yif1a	0.53599343	0.04323688	Mtm1y/-_UP
Ogfod3	0.53628772	0.00754365	Mtm1y/-_UP

Capn1	0.53662036	0.03406678	Mtm1y/-_UP
Ncstn	0.53844582	0.00810111	Mtm1y/-_UP
Micu2	0.53930601	0.01665419	Mtm1y/-_UP
Ran	0.54434901	0.0440039	Mtm1y/-_UP
Tmed5	0.54669876	0.02829425	Mtm1y/-_UP
Glg1	0.54742336	0.00872229	Mtm1y/-_UP
Fhl1	0.54751	0.00806212	Mtm1y/-_UP
Hint1	0.54925909	0.00119245	Mtm1y/-_UP
Anxa11	0.54990484	0.02759232	Mtm1y/-_UP
Lman2l	0.55176533	0.01736447	Mtm1y/-_UP
Glrx	0.55177229	0.03142177	Mtm1y/-_UP
Atp6v1b2	0.55346465	0.01664447	Mtm1y/-_UP
Camk2a	0.55613276	0.03109448	Mtm1y/-_UP
Mrpl1	0.55718865	0.03327076	Mtm1y/-_UP
Vim	0.55813047	0.03042937	Mtm1y/-_UP
Pomgnt1	0.55920679	0.01327865	Mtm1y/-_UP
Lypla2	0.56293836	0.0440039	Mtm1y/-_UP
Ccpg1	0.56329747	0.02475125	Mtm1y/-_UP
Hyou1	0.56697875	0.00246199	Mtm1y/-_UP
Eif3d	0.56749012	0.03271331	Mtm1y/-_UP
Ptpn3	0.56807034	0.02117357	Mtm1y/-_UP
Eef1a1	0.56906882	0.0028082	Mtm1y/-_UP
Erap1	0.57113517	0.03301036	Mtm1y/-_UP
Atp6v1a	0.5711654	0.00094348	Mtm1y/-_UP
Esyt2	0.57396831	0.00972013	Mtm1y/-_UP
Macf1	0.57446538	0.0253275	Mtm1y/-_UP
Sgpl1	0.57506235	0.04789589	Mtm1y/-_UP
B3galnt2	0.5763221	0.02989426	Mtm1y/-_UP
Ptprm	0.57827061	0.03179855	Mtm1y/-_UP
Copb1	0.57828047	0.00845322	Mtm1y/-_UP
Rasa4	0.57852043	0.03042937	Mtm1y/-_UP
Gnai3	0.57983741	0.04993527	Mtm1y/-_UP

Eif1	0.58240698	0.02463229	Mtm1y/-_UP
Tuba1c	0.58406691	0.02580807	Mtm1y/-_UP
Atp6v1e1	0.58536912	0.01960636	Mtm1y/-_UP
Stt3a	0.58564255	0.00781883	Mtm1y/-_UP
Map4	0.58586711	0.04911358	Mtm1y/-_UP
Hsd17b12	0.58736554	0.00823556	Mtm1y/-_UP
Cyb5d2	0.58762079	0.01792204	Mtm1y/-_UP
Atp6v1g1	0.58944381	0.03161721	Mtm1y/-_UP
Psma5	0.58966533	0.03098856	Mtm1y/-_UP
Fkbp3	0.59020852	0.04756837	Mtm1y/-_UP
Use1	0.59243325	0.03537759	Mtm1y/-_UP
Glod4	0.59302915	0.01959089	Mtm1y/-_UP
Pon3	0.59313676	0.01940062	Mtm1y/-_UP
Selenof	0.59435647	0.02276583	Mtm1y/-_UP
Mindy1	0.59670329	0.04372355	Mtm1y/-_UP
Micu1	0.59729094	0.00266057	Mtm1y/-_UP
Parva	0.59751744	0.01609468	Mtm1y/-_UP
Dnajc10	0.59819345	0.00581109	Mtm1y/-_UP
Rragc	0.59961041	0.02374279	Mtm1y/-_UP
Sf3b1	0.60082109	0.01469282	Mtm1y/-_UP
Hnrnph2	0.60305064	0.03352086	Mtm1y/-_UP
Hnrnph1	0.60314057	0.03902682	Mtm1y/-_UP
Atp6v0a1	0.60318059	0.00668997	Mtm1y/-_UP
Tor1b	0.60401279	0.00523129	Mtm1y/-_UP
Styxl2	0.60476085	0.017533	Mtm1y/-_UP
Cmpk1	0.60920689	0.02910157	Mtm1y/-_UP
Nop58	0.61030786	0.04993527	Mtm1y/-_UP
Sdf2	0.61298405	0.00428611	Mtm1y/-_UP
Nsdhl	0.61505913	0.02738138	Mtm1y/-_UP
Fhod1	0.61568289	0.00980885	Mtm1y/-_UP
Pusl1	0.61666353	0.02644254	Mtm1y/-_UP
Utrn	0.62207327	0.0364311	Mtm1y/-_UP

Bin1	0.62518047	0.01960636	Mtm1y/-_UP
Cant1	0.62573169	0.04174763	Mtm1y/-_UP
Naa50	0.62669306	0.01086509	Mtm1y/-_UP
Tmed10	0.62759067	0.00987415	Mtm1y/-_UP
Vars2	0.62905494	0.02065466	Mtm1y/-_UP
Prxl2b	0.63008181	0.01665329	Mtm1y/-_UP
U2af1	0.63278477	0.04031388	Mtm1y/-_UP
Erp44	0.63553626	0.0145517	Mtm1y/-_UP
Rab7a	0.63563353	0.00094348	Mtm1y/-_UP
Nucb1	0.63915514	0.00227109	Mtm1y/-_UP
Tmed1	0.63943914	0.00444848	Mtm1y/-_UP
Ano6	0.63958046	0.03098856	Mtm1y/-_UP
Vapa	0.64105722	0.00258289	Mtm1y/-_UP
Tmx3	0.64293462	0.00188855	Mtm1y/-_UP
Tnpo3	0.64393303	0.0440039	Mtm1y/-_UP
Atp6v0d1	0.64618124	0.0008734	Mtm1y/-_UP
Sf3b3	0.64630484	0.03161721	Mtm1y/-_UP
Gtpbp3	0.64637809	0.04756837	Mtm1y/-_UP
Selenoi	0.64673025	0.02733489	Mtm1y/-_UP
Tmx4	0.64693174	0.03800795	Mtm1y/-_UP
Psmb1	0.64696313	0.04798047	Mtm1y/-_UP
B3galt6	0.65108902	0.01792204	Mtm1y/-_UP
Bpnt2	0.65244734	0.01031547	Mtm1y/-_UP
Cpd	0.65364799	0.00302254	Mtm1y/-_UP
Lpgat1	0.65477993	0.03406678	Mtm1y/-_UP
Asph	0.65875699	0.02488242	Mtm1y/-_UP
Mrps24	0.65966344	0.02801474	Mtm1y/-_UP
Esyt1	0.66012034	0.00267847	Mtm1y/-_UP
Mrpl22	0.66058263	0.01521589	Mtm1y/-_UP
Camk2b	0.66160708	0.02382735	Mtm1y/-_UP
Mrpl52	0.66277362	0.0440039	Mtm1y/-_UP
Pdia4	0.66356015	0.0090194	Mtm1y/-_UP

Rnh1	0.66593885	0.00342091	Mtm1y/-_UP
Pet117	0.66690209	0.04384376	Mtm1y/-_UP
Sod1	0.66787064	0.03999167	Mtm1y/-_UP
Tom1	0.66855046	0.02100579	Mtm1y/-_UP
Rusf1	0.66869169	0.00251422	Mtm1y/-_UP
Myl1	0.67049982	0.04201775	Mtm1y/-_UP
Klhl31	0.67176521	0.04142805	Mtm1y/-_UP
Mcu	0.67191005	0.01609468	Mtm1y/-_UP
Dnajb11	0.67490938	0.00103778	Mtm1y/-_UP
Fgfrl1	0.67588664	0.0212068	Mtm1y/-_UP
Prpf6	0.67605676	0.01376163	Mtm1y/-_UP
Atp6v1h	0.67671299	0.01086509	Mtm1y/-_UP
Eif5a	0.67736739	0.00120056	Mtm1y/-_UP
Mrps33	0.67856055	0.03653964	Mtm1y/-_UP
Erlec1	0.68107305	0.00342353	Mtm1y/-_UP
Vapb	0.68227439	0.00447382	Mtm1y/-_UP
Hnrnpm	0.68273139	0.00267847	Mtm1y/-_UP
P4hb	0.68342139	0.02580807	Mtm1y/-_UP
Hnrnpa2b1	0.68475448	0.00729495	Mtm1y/-_UP
Hsp90b1	0.68576026	0.00235121	Mtm1y/-_UP
Txndc5	0.69050066	0.01301628	Mtm1y/-_UP
Atp1b3	0.69196513	0.02835636	Mtm1y/-_UP
Mogs	0.69481449	0.00239301	Mtm1y/-_UP
Ergic2	0.69497722	0.00659322	Mtm1y/-_UP
Eef1g	0.69517096	0.00810111	Mtm1y/-_UP
Tnxb	0.69624206	0.03394029	Mtm1y/-_UP
Cdh13	0.69742328	0.04055681	Mtm1y/-_UP
Stx12	0.69872584	0.04530995	Mtm1y/-_UP
Clptm1l	0.69943116	0.02228774	Mtm1y/-_UP
Mff	0.703399	0.03161721	Mtm1y/-_UP
Atp6v1c1	0.70395465	0.02435883	Mtm1y/-_UP
Ergic3	0.70513401	0.00538639	Mtm1y/-_UP

Elac2	0.70632685	0.03355193	Mtm1y/-_UP
Nt5dc3	0.70941407	0.00850627	Mtm1y/-_UP
Rcn2	0.71076213	0.02243489	Mtm1y/-_UP
Spcs1	0.7127419	0.01107467	Mtm1y/-_UP
Sdf2l1	0.71910326	0.01143754	Mtm1y/-_UP
Hdgf	0.72019706	0.03252722	Mtm1y/-_UP
Alg2	0.72030418	0.01348706	Mtm1y/-_UP
Dnase1l1	0.7211727	0.04145448	Mtm1y/-_UP
Kars1	0.72248454	0.00208361	Mtm1y/-_UP
Pgd	0.72281056	0.00385513	Mtm1y/-_UP
Enpp4	0.72777902	0.00734774	Mtm1y/-_UP
Sec11a	0.73142611	0.01313359	Mtm1y/-_UP
Vti1b	0.73288431	0.02030661	Mtm1y/-_UP
Ahsa1	0.73343174	0.04724515	Mtm1y/-_UP
Apoa4	0.73432259	0.01051887	Mtm1y/-_UP
Tor3a	0.73494685	0.00602002	Mtm1y/-_UP
Tubb3	0.73511891	0.03609991	Mtm1y/-_UP
Hnrnph3	0.7351734	0.0441642	Mtm1y/-_UP
Extl3	0.73520171	0.01537563	Mtm1y/-_UP
Scarb2	0.73547879	0.00258289	Mtm1y/-_UP
Mtrf1	0.73599474	0.02774077	Mtm1y/-_UP
Ssr3	0.73600331	0.04031388	Mtm1y/-_UP
Bola2	0.73856532	0.03394408	Mtm1y/-_UP
Psma2	0.74015938	0.04228496	Mtm1y/-_UP
Asph	0.74058498	0.01665419	Mtm1y/-_UP
Tmco1	0.74189013	0.01857438	Mtm1y/-_UP
Abhd17b	0.74428556	0.03212957	Mtm1y/-_UP
Trnt1	0.74547858	0.03252722	Mtm1y/-_UP
Plg	0.74777287	0.01241385	Mtm1y/-_UP
Tm9sf3	0.7479986	0.00532202	Mtm1y/-_UP
Srl	0.74802777	0.0050427	Mtm1y/-_UP
Tm9sf4	0.74958098	0.00981371	Mtm1y/-_UP

Cemip2	0.75485832	0.00245829	Mtm1y/-_UP
Faf2	0.75813172	0.03537759	Mtm1y/-_UP
Psma3	0.75987925	0.01435037	Mtm1y/-_UP
Pdia6	0.76024303	0.01249947	Mtm1y/-_UP
Itga5	0.76065789	0.02989426	Mtm1y/-_UP
Tor1aip2	0.76252961	0.00477698	Mtm1y/-_UP
Ptges3	0.76262152	0.00385513	Mtm1y/-_UP
Pisd	0.76590326	0.00885501	Mtm1y/-_UP
Psmb2	0.76620677	0.00973614	Mtm1y/-_UP
Cope	0.76788968	0.02902819	Mtm1y/-_UP
Tm9sf2	0.77144084	0.00173833	Mtm1y/-_UP
Hm13	0.77167855	0.01246934	Mtm1y/-_UP
Serinc1	0.77173454	0.02149831	Mtm1y/-_UP
Pdia3	0.77231971	0.02123233	Mtm1y/-_UP
Syvn1	0.77379363	0.03579174	Mtm1y/-_UP
Card19	0.77617354	0.0023638	Mtm1y/-_UP
Mtpn	0.77647575	0.01585332	Mtm1y/-_UP
Thoc2	0.77732263	0.01893659	Mtm1y/-_UP
Aplp2	0.78010939	0.03269609	Mtm1y/-_UP
Arf6	0.78032765	0.00323623	Mtm1y/-_UP
Rbm24	0.78262987	0.01668091	Mtm1y/-_UP
Yme1l1	0.78463437	0.03139357	Mtm1y/-_UP
Psmb7	0.78620253	0.01212165	Mtm1y/-_UP
Npm1	0.78670326	0.02975669	Mtm1y/-_UP
Shisa4	0.78819359	0.02984739	Mtm1y/-_UP
Ubqln1	0.79172815	0.01301243	Mtm1y/-_UP
Mif	0.79180647	0.02801474	Mtm1y/-_UP
Bzw1	0.79547941	0.02738138	Mtm1y/-_UP
Prkab2	0.79666272	0.01499929	Mtm1y/-_UP
Abcg2	0.79923589	0.04911358	Mtm1y/-_UP
Sec22b	0.79925643	0.00612026	Mtm1y/-_UP
Sppl2b	0.80033419	0.02835636	Mtm1y/-_UP

Eef1d	0.8009785	0.0190986	Mtm1y/-_UP
Hnrnpa3	0.80110221	0.02153545	Mtm1y/-_UP
Ube2i	0.80336795	0.022668	Mtm1y/-_UP
Gpr107	0.80683801	0.00150238	Mtm1y/-_UP
Asah1	0.80806858	0.00659322	Mtm1y/-_UP
Htra2	0.81151343	0.04789589	Mtm1y/-_UP
Gba1	0.81246056	0.00581109	Mtm1y/-_UP
Fkbp9	0.81543449	0.03139357	Mtm1y/-_UP
Igf2r	0.8155554	0.00188855	Mtm1y/-_UP
Smarca4	0.81586886	0.02873787	Mtm1y/-_UP
Gstm2	0.81702897	0.01051887	Mtm1y/-_UP
Tmem41b	0.81820234	0.02807068	Mtm1y/-_UP
Nln	0.820311	0.02975669	Mtm1y/-_UP
Vkorc1	0.82089162	0.04756837	Mtm1y/-_UP
4931406C07Rik	0.82119599	0.00520904	Mtm1y/-_UP
Mob4	0.82196743	0.00581109	Mtm1y/-_UP
Atp6v0c	0.8220592	0.00266057	Mtm1y/-_UP
Slc44a1	0.82244911	0.04655638	Mtm1y/-_UP
Mia3	0.82496545	0.00411909	Mtm1y/-_UP
Epdr1	0.82602255	0.00698448	Mtm1y/-_UP
Dnajc3	0.8289376	0.00703313	Mtm1y/-_UP
Wipi1	0.8296652	0.02989426	Mtm1y/-_UP
Fam136a	0.83320426	0.04176531	Mtm1y/-_UP
Fkbp5	0.83341109	0.02230589	Mtm1y/-_UP
Alg5	0.83470653	0.02873787	Mtm1y/-_UP
Pofut1	0.83526995	0.00342091	Mtm1y/-_UP
Itgb1bp2	0.8360762	0.01857438	Mtm1y/-_UP
Ctsl	0.83750033	0.02435208	Mtm1y/-_UP
Kpna3	0.83869189	0.02153545	Mtm1y/-_UP
Ca3	0.83966688	0.0264425	Mtm1y/-_UP
Shmt2	0.83989817	0.02169053	Mtm1y/-_UP
Sel1l	0.84316669	0.00135676	Mtm1y/-_UP

Ier3ip1	0.84401528	0.01178593	Mtm1y/-_UP
Myl10	0.84486462	0.04789589	Mtm1y/-_UP
Acbd3	0.84541047	0.00833843	Mtm1y/-_UP
Eno1	0.8468152	0.03788541	Mtm1y/-_UP
G6pc3	0.84752977	0.01857438	Mtm1y/-_UP
Ppia	0.84907793	0.00473562	Mtm1y/-_UP
Hnrnpa0	0.85163315	0.02677832	Mtm1y/-_UP
Mapre3	0.85234638	0.01665419	Mtm1y/-_UP
Api5	0.85302623	0.01736447	Mtm1y/-_UP
Dhrsx	0.85339016	0.02242091	Mtm1y/-_UP
Golt1b	0.85509714	0.0462145	Mtm1y/-_UP
Rer1	0.85524277	0.00789712	Mtm1y/-_UP
Sec61g	0.85548876	0.03161721	Mtm1y/-_UP
Ctsd	0.85688259	0.01940062	Mtm1y/-_UP
Tmem167	0.85837244	0.00296904	Mtm1y/-_UP
Carhsp1	0.86219078	0.01905863	Mtm1y/-_UP
Ubxn4	0.86494136	0.0440039	Mtm1y/-_UP
Sacs	0.86600994	0.03179855	Mtm1y/-_UP
Dpm1	0.86846463	0.02198061	Mtm1y/-_UP
Ubxn1	0.87018929	0.03650526	Mtm1y/-_UP
Igf1r	0.87170191	0.04934472	Mtm1y/-_UP
Galnt2	0.87313348	0.00083054	Mtm1y/-_UP
Galnt7	0.87330462	0.02929605	Mtm1y/-_UP
Cst3	0.87460321	0.00385513	Mtm1y/-_UP
Arf5	0.87522462	0.03314839	Mtm1y/-_UP
Neo1	0.87620808	0.02119231	Mtm1y/-_UP
Gsr	0.87625976	0.01146351	Mtm1y/-_UP
Cox11	0.87951508	0.0253275	Mtm1y/-_UP
Ckap5	0.88179923	0.00565348	Mtm1y/-_UP
Anxa4	0.88370959	0.00117581	Mtm1y/-_UP
Psmb4	0.88459897	0.01544726	Mtm1y/-_UP
Gusb	0.88695792	0.01592506	Mtm1y/-_UP

Rnf13	0.89085702	0.01515378	Mtm1y/-_UP
Smdt1	0.89365793	0.01485337	Mtm1y/-_UP
Pgrmc2	0.89762924	0.01094457	Mtm1y/-_UP
Gpr89	0.89856527	0.01693163	Mtm1y/-_UP
Jsrp1	0.89865404	0.00266057	Mtm1y/-_UP
Tpt1	0.8988603	0.00083054	Mtm1y/-_UP
Ampd3	0.89989192	0.00612026	Mtm1y/-_UP
Txn	0.90178891	0.00083054	Mtm1y/-_UP
Pafah1b1	0.90307654	0.01753874	Mtm1y/-_UP
Dynlt1a	0.90441944	0.04870637	Mtm1y/-_UP
Gpx1	0.90520464	0.00239301	Mtm1y/-_UP
Psmb3	0.90701342	0.03305516	Mtm1y/-_UP
Tmem131	0.90715506	0.00456354	Mtm1y/-_UP
Adpgk	0.90789874	0.00140564	Mtm1y/-_UP
Ctsa	0.90854719	0.00083054	Mtm1y/-_UP
Ccn2	0.90900781	0.03989619	Mtm1y/-_UP
Bola1	0.90944173	0.03139357	Mtm1y/-_UP
Rbp1	0.91061619	0.04787641	Mtm1y/-_UP
Dipk1a	0.91394844	0.01693163	Mtm1y/-_UP
Polr2h	0.91451254	0.04158156	Mtm1y/-_UP
Naxe	0.91858431	0.01974151	Mtm1y/-_UP
Rab6a	0.91867963	0.04911358	Mtm1y/-_UP
Snd1	0.92221437	0.00662002	Mtm1y/-_UP
Ncs1	0.92402814	0.01572423	Mtm1y/-_UP
Psmb5	0.92515356	0.01418117	Mtm1y/-_UP
Slc9a1	0.92704879	0.02119231	Mtm1y/-_UP
Plp2	0.93229588	0.01178593	Mtm1y/-_UP
Abat	0.93981805	0.0097603	Mtm1y/-_UP
Purb	0.94009674	0.01148219	Mtm1y/-_UP
Gorasp2	0.94274389	0.04911358	Mtm1y/-_UP
Txndc17	0.94387201	0.00472417	Mtm1y/-_UP
Cspg4	0.94923034	0.00408585	Mtm1y/-_UP

Erp29	0.95835472	0.00235121	Mtm1y/-_UP
Dynll1	0.96065595	0.01665419	Mtm1y/-_UP
Erlin2	0.96071774	0.00140564	Mtm1y/-_UP
Plbd2	0.96111467	0.0212068	Mtm1y/-_UP
Coq8b	0.96265845	0.03400269	Mtm1y/-_UP
Lum	0.96306721	0.00827604	Mtm1y/-_UP
Sh3bgrl	0.96433199	0.00754825	Mtm1y/-_UP
Twsg1	0.96554345	0.02455894	Mtm1y/-_UP
Dusp3	0.97172634	0.00154872	Mtm1y/-_UP
Spcs2	0.97988212	0.01643177	Mtm1y/-_UP
Clic4	0.98282735	0.01187909	Mtm1y/-_UP
Serbp1	0.98370836	0.03419502	Mtm1y/-_UP
Polr2l	0.98605856	0.03183311	Mtm1y/-_UP
Edem3	0.98897113	0.00342091	Mtm1y/-_UP
Diablo	0.99077916	0.00084105	Mtm1y/-_UP
Csgalnact2	0.9910768	0.01139699	Mtm1y/-_UP
Tapbp	0.99151237	0.04356963	Mtm1y/-_UP
Ppic	0.992271	0.02902819	Mtm1y/-_UP
Cap2	0.99739268	0.00703313	Mtm1y/-_UP
Serping1	1.00185095	0.00698448	Mtm1y/-_UP
Lmna	1.00611546	0.00827818	Mtm1y/-_UP
Atox1	1.00825151	0.02519805	Mtm1y/-_UP
Mycbp2	1.0089017	0.00428611	Mtm1y/-_UP
Rras	1.00914594	0.00958586	Mtm1y/-_UP
Tmed7	1.00986303	0.02175898	Mtm1y/-_UP
Acp2	1.01250097	0.01892298	Mtm1y/-_UP
Aspn	1.01287333	0.01014297	Mtm1y/-_UP
Naga	1.01809214	0.00188855	Mtm1y/-_UP
Pon2	1.02132784	0.0028082	Mtm1y/-_UP
Prrc1	1.0243964	0.02975669	Mtm1y/-_UP
Pycr2	1.02651352	0.002692	Mtm1y/-_UP
Hyi	1.0268202	0.03117119	Mtm1y/-_UP

Poglut3	1.02757369	0.00659322	Mtm1y/-_UP
Tmed3	1.03073448	0.00679399	Mtm1y/-_UP
Add3	1.03162413	0.01643177	Mtm1y/-_UP
Lgmn	1.03239706	0.0050427	Mtm1y/-_UP
H13	1.03487176	0.03042937	Mtm1y/-_UP
Lman2	1.03861162	0.00015212	Mtm1y/-_UP
Ergic1	1.0407917	0.00135676	Mtm1y/-_UP
Tgoln1	1.04736292	0.00668997	Mtm1y/-_UP
Hsd11b1	1.0496099	0.03098347	Mtm1y/-_UP
Chid1	1.05130952	0.00810111	Mtm1y/-_UP
Aldh1b1	1.05301327	0.02919739	Mtm1y/-_UP
Galns	1.05868362	0.04789589	Mtm1y/-_UP
Vma21	1.05993286	0.02140964	Mtm1y/-_UP
Dhrs7	1.06143875	0.00530312	Mtm1y/-_UP
Dpp3	1.06213606	0.00778071	Mtm1y/-_UP
Slc38a2	1.0641172	0.0392291	Mtm1y/-_UP
Lmo7	1.07034043	0.00473562	Mtm1y/-_UP
Tmem87a	1.07541861	0.00744271	Mtm1y/-_UP
Cox17	1.07828195	0.04036612	Mtm1y/-_UP
Antkmt	1.07868093	0.00110855	Mtm1y/-_UP
Eef1b	1.08014321	0.02366211	Mtm1y/-_UP
Golim4	1.08252855	0.02065652	Mtm1y/-_UP
Abhd4	1.08324364	0.04626722	Mtm1y/-_UP
Dbi	1.08333845	0.01107467	Mtm1y/-_UP
Anxa8	1.08463121	0.02950399	Mtm1y/-_UP
Lipt2	1.09049519	0.00964696	Mtm1y/-_UP
Adipoq	1.09175072	0.03224034	Mtm1y/-_UP
Wnt9a	1.09235406	0.01261577	Mtm1y/-_UP
Grb10	1.09464194	0.02917897	Mtm1y/-_UP
Mybpc1	1.10194195	0.03098856	Mtm1y/-_UP
Hebp1	1.10314711	0.00754197	Mtm1y/-_UP
Pfn1	1.10367724	0.00405103	Mtm1y/-_UP

B4galt1	1.10480567	0.00322728	Mtm1y/-_UP
Tgfb2	1.10594306	0.01327865	Mtm1y/-_UP
Rdh11	1.10710614	0.00211127	Mtm1y/-_UP
Npc2	1.10801896	0.01824044	Mtm1y/-_UP
Pla2g6	1.1095517	0.03098856	Mtm1y/-_UP
Calu	1.11055233	0.00823556	Mtm1y/-_UP
Blmh	1.11323445	0.04743239	Mtm1y/-_UP
Itm2b	1.12112191	0.01645187	Mtm1y/-_UP
Akt2	1.12122123	0.00048105	Mtm1y/-_UP
Anxa7	1.12567099	0.00205117	Mtm1y/-_UP
Traf2	1.12607758	0.03924923	Mtm1y/-_UP
Txndc12	1.1363623	0.01792204	Mtm1y/-_UP
Slc2a1	1.13729777	0.03534499	Mtm1y/-_UP
Lcp1	1.145672	0.04031388	Mtm1y/-_UP
Sbspon	1.14608593	0.04226615	Mtm1y/-_UP
Cfd	1.14871373	0.01792204	Mtm1y/-_UP
Lyplal1	1.14899872	0.00258289	Mtm1y/-_UP
Cobl	1.14933317	0.0440039	Mtm1y/-_UP
Kiaa2013	1.15085662	0.00698448	Mtm1y/-_UP
Uxs1	1.15109327	0.00122395	Mtm1y/-_UP
Flrt2	1.15112957	0.00581109	Mtm1y/-_UP
Galnt1	1.15137886	0.00258289	Mtm1y/-_UP
Pex5	1.15353467	0.03014907	Mtm1y/-_UP
Slc35a3	1.1536126	0.03269609	Mtm1y/-_UP
Fkbp7	1.1555644	0.00842041	Mtm1y/-_UP
Pcyox1l	1.15700709	0.00398026	Mtm1y/-_UP
Plod2	1.1603678	0.02325362	Mtm1y/-_UP
Ecm1	1.16132314	0.00039887	Mtm1y/-_UP
Anpep	1.16207331	0.03139357	Mtm1y/-_UP
Dysf	1.16432372	0.04384376	Mtm1y/-_UP
Psma4	1.16492037	0.00171977	Mtm1y/-_UP
Crtap	1.16916005	0.03410725	Mtm1y/-_UP

Add1	1.17031292	0.00729495	Mtm1y/-_UP
Ehbp1l1	1.17107844	0.00754365	Mtm1y/-_UP
Zbed5	1.1737201	0.00529698	Mtm1y/-_UP
Syne1	1.17560564	0.04338801	Mtm1y/-_UP
Gaa	1.17711372	0.00076649	Mtm1y/-_UP
Me2	1.17895997	0.00083054	Mtm1y/-_UP
Pin1	1.17947887	0.01995004	Mtm1y/-_UP
Rint1	1.1798656	0.0075836	Mtm1y/-_UP
Fbxo30	1.18414397	0.01451047	Mtm1y/-_UP
Adgre5	1.18521593	0.00188855	Mtm1y/-_UP
Fkbp10	1.18625841	0.02677832	Mtm1y/-_UP
Obsl1	1.18736499	0.01932822	Mtm1y/-_UP
Mlec	1.18778297	0.00165166	Mtm1y/-_UP
Xirp1	1.18877158	0.00760896	Mtm1y/-_UP
Tmem62	1.1904043	0.03426089	Mtm1y/-_UP
Neb	1.19281353	0.04659345	Mtm1y/-_UP
S100a16	1.19302741	0.03629378	Mtm1y/-_UP
Set	1.19604322	0.00267847	Mtm1y/-_UP
Cd99l2	1.19872196	0.00135676	Mtm1y/-_UP
Ablim1	1.20134954	0.00930746	Mtm1y/-_UP
Vps52	1.20553066	0.02921538	Mtm1y/-_UP
Arf4	1.20587713	0.01013171	Mtm1y/-_UP
Yipf6	1.21024784	0.00083054	Mtm1y/-_UP
Timp2	1.21195232	0.0190986	Mtm1y/-_UP
Dipk2a	1.21257129	0.0253275	Mtm1y/-_UP
Aldh18a1	1.21493103	0.00490834	Mtm1y/-_UP
Vwa5a	1.2167186	0.0002311	Mtm1y/-_UP
Cdnf	1.21858054	0.00188855	Mtm1y/-_UP
Krt18	1.2231825	0.03098856	Mtm1y/-_UP
Lrrn1	1.22395163	0.00438691	Mtm1y/-_UP
Cd63	1.22526932	0.01665419	Mtm1y/-_UP
Plxdc2	1.2257676	0.0022845	Mtm1y/-_UP

Hspb8	1.23200056	0.01285797	Mtm1y/-_UP
Prune2	1.2321768	0.00609129	Mtm1y/-_UP
Fut8	1.23523915	0.0440039	Mtm1y/-_UP
Hars1	1.23783922	0.01149198	Mtm1y/-_UP
Mtdh	1.23805162	0.00122395	Mtm1y/-_UP
Galnt17	1.24023873	0.00267847	Mtm1y/-_UP
Hal	1.24823014	0.03640975	Mtm1y/-_UP
Hmgn2	1.25214068	0.03042937	Mtm1y/-_UP
Pgam5	1.2588658	0.00160883	Mtm1y/-_UP
Eif1a	1.25968397	0.00071812	Mtm1y/-_UP
H2-Aa	1.2602833	0.01989094	Mtm1y/-_UP
Cybc1	1.27569765	0.00103778	Mtm1y/-_UP
Poglut1	1.27622078	0.00563316	Mtm1y/-_UP
Col5a2	1.29098877	0.03271331	Mtm1y/-_UP
Sqstm1	1.29412863	0.01952776	Mtm1y/-_UP
Cox19	1.30181482	0.01673349	Mtm1y/-_UP
Ap2s1	1.30626511	0.00648976	Mtm1y/-_UP
Osbpl8	1.30635079	0.00698448	Mtm1y/-_UP
Sptbn2	1.30666866	0.04890803	Mtm1y/-_UP
Efemp1	1.30729232	0.00375243	Mtm1y/-_UP
Vps33b	1.31241391	0.00761253	Mtm1y/-_UP
Tsg101	1.31308164	0.04205619	Mtm1y/-_UP
Dpm3	1.31432447	0.00648976	Mtm1y/-_UP
Cnpy3	1.31614118	0.00566046	Mtm1y/-_UP
Tmem43	1.31654314	0.00120056	Mtm1y/-_UP
Fth1	1.3182892	0.00664888	Mtm1y/-_UP
Ces2c	1.32639087	0.01736447	Mtm1y/-_UP
Ppib	1.32775294	0.00083054	Mtm1y/-_UP
Acot9	1.33049311	0.00105906	Mtm1y/-_UP
Gtpbp6	1.33641095	0.04100265	Mtm1y/-_UP
Jag2	1.33765318	0.03365348	Mtm1y/-_UP
Egf	1.33924588	0.0106449	Mtm1y/-_UP

Pycr1	1.34094715	0.00083054	Mtm1y/-_UP
Eif6	1.34167847	0.00795739	Mtm1y/-_UP
Lgals3	1.35578865	0.00817409	Mtm1y/-_UP
Pvr	1.36136979	0.02230589	Mtm1y/-_UP
Mesd	1.36443552	0.00083054	Mtm1y/-_UP
Nme1	1.3767449	0.00247209	Mtm1y/-_UP
Hrg	1.38198979	0.00239301	Mtm1y/-_UP
Sec11c	1.3827047	0.01572423	Mtm1y/-_UP
Fbln1	1.38270738	0.01178593	Mtm1y/-_UP
Cul7	1.38879285	0.03732606	Mtm1y/-_UP
Xirp2	1.38979421	0.01301628	Mtm1y/-_UP
Cd44	1.39146066	0.01266985	Mtm1y/-_UP
Cyb561d2	1.39855545	0.00245829	Mtm1y/-_UP
Cd55	1.4016787	0.00084136	Mtm1y/-_UP
Stom	1.4082876	0.00691404	Mtm1y/-_UP
Hprt1	1.41003127	0.00110855	Mtm1y/-_UP
Vat1	1.41266918	0.017533	Mtm1y/-_UP
Mtrex	1.42417983	0.02984739	Mtm1y/-_UP
Ptprc	1.42799933	0.04158156	Mtm1y/-_UP
Lrpap1	1.43015321	0.00077147	Mtm1y/-_UP
None	1.43119362	0.03488738	Mtm1y/-_UP
Rpl3	1.44083165	0.02110493	Mtm1y/-_UP
Cpe	1.44209185	0.00532202	Mtm1y/-_UP
Cnpy2	1.44627192	0.00235121	Mtm1y/-_UP
C3	1.45622102	0.0017535	Mtm1y/-_UP
Ddah2	1.45813698	0.01693163	Mtm1y/-_UP
Acvr2a	1.45867481	0.03977552	Mtm1y/-_UP
Serpinb6a	1.46175738	5.76E-05	Mtm1y/-_UP
Pam	1.47163649	0.00105906	Mtm1y/-_UP
Rnaset2b	1.4730938	0.0019794	Mtm1y/-_UP
Tgm1	1.47763116	0.04540771	Mtm1y/-_UP
Mcl1	1.48411005	0.00105906	Mtm1y/-_UP

Snrpc	1.48505706	0.04787641	Mtm1y/-_UP
Krt80	1.48939017	0.03421316	Mtm1y/-_UP
Pyroxd2	1.49161999	9.98E-05	Mtm1y/-_UP
Eda2r	1.49320241	0.02910157	Mtm1y/-_UP
Ltbp3	1.50173104	0.00131135	Mtm1y/-_UP
Cyb5r3	1.50185772	0.00020823	Mtm1y/-_UP
Prkag3	1.50275599	0.00011985	Mtm1y/-_UP
Otulin	1.50286274	0.01785726	Mtm1y/-_UP
Gsn	1.50563058	0.00258289	Mtm1y/-_UP
Fbln5	1.51019821	0.00208361	Mtm1y/-_UP
Fn1	1.51080276	0.00188855	Mtm1y/-_UP
Plxnb2	1.51820468	0.00189316	Mtm1y/-_UP
Rrbp1	1.52824198	0.03653964	Mtm1y/-_UP
Fbln2	1.5400235	0.00523516	Mtm1y/-_UP
Fst	1.55123278	0.01665419	Mtm1y/-_UP
Krt2	1.55625928	0.03098856	Mtm1y/-_UP
Camk2d	1.55653514	0.00015212	Mtm1y/-_UP
Calu	1.55758409	0.00103272	Mtm1y/-_UP
Tmem205	1.56425955	0.00188855	Mtm1y/-_UP
Nipsnap3b	1.56750325	0.00239301	Mtm1y/-_UP
Ggct	1.5695084	0.0479298	Mtm1y/-_UP
Cfh	1.57327302	0.00659322	Mtm1y/-_UP
Itih3	1.5808134	0.00659322	Mtm1y/-_UP
Ckap4	1.58531517	0.00398026	Mtm1y/-_UP
Nudt16	1.58668253	0.00995555	Mtm1y/-_UP
Art5	1.59502677	0.04158341	Mtm1y/-_UP
Atp5if1	1.59653654	0.00488558	Mtm1y/-_UP
Aif1l	1.60889694	0.04724515	Mtm1y/-_UP
Pgam5	1.61006079	0.00267155	Mtm1y/-_UP
Crip1	1.61911645	0.02112543	Mtm1y/-_UP
Tmbim1	1.6233836	0.02533961	Mtm1y/-_UP
Srsf3	1.63759504	0.00122395	Mtm1y/-_UP

Rhoc	1.6464641	0.00322728	Mtm1y/-_UP
Ftl1	1.64770618	0.01241385	Mtm1y/-_UP
Sod3	1.65894213	0.00068774	Mtm1y/-_UP
Dsg1a	1.66209278	0.03352086	Mtm1y/-_UP
Islr	1.67671368	0.02152821	Mtm1y/-_UP
Asl	1.67859886	0.01347994	Mtm1y/-_UP
Gnai1	1.67866344	0.02927877	Mtm1y/-_UP
Thbs4	1.68014688	0.03427583	Mtm1y/-_UP
Gpc1	1.68070709	0.00060035	Mtm1y/-_UP
Trpd52l3	1.68379729	0.00362666	Mtm1y/-_UP
Man2a2	1.68621775	0.00020823	Mtm1y/-_UP
Smoc2	1.68631118	0.00686895	Mtm1y/-_UP
Atp2a1	1.69154866	0.00188855	Mtm1y/-_UP
Tgm3	1.69303538	0.03307256	Mtm1y/-_UP
Cstb	1.69543143	0.00060035	Mtm1y/-_UP
Manf	1.70123533	0.00296904	Mtm1y/-_UP
Ighg3	1.70157576	0.02984739	Mtm1y/-_UP
Nup133	1.70628985	0.00795739	Mtm1y/-_UP
S100a3	1.70744469	0.00083054	Mtm1y/-_UP
Cygb	1.71007457	0.00361646	Mtm1y/-_UP
Krt1	1.72596726	0.03629378	Mtm1y/-_UP
Ctsz	1.72769885	0.02463229	Mtm1y/-_UP
Sort1	1.73571794	0.00798022	Mtm1y/-_UP
Ighg2b	1.74271038	0.03142177	Mtm1y/-_UP
Mlf2	1.74340098	0.03426089	Mtm1y/-_UP
Lgals1	1.75113031	0.00245855	Mtm1y/-_UP
Dclk1	1.75574655	0.02441758	Mtm1y/-_UP
Ifitm3	1.76827359	0.01327865	Mtm1y/-_UP
Ccdc134	1.78351799	0.00083054	Mtm1y/-_UP
Kdelr2	1.7977712	0.03098347	Mtm1y/-_UP
Tgfb3	1.80292554	0.00581109	Mtm1y/-_UP
Uchl1	1.80456313	0.01435037	Mtm1y/-_UP

Htra1	1.81652725	0.01010781	Mtm1y/-_UP
Mvp	1.81833359	0.03653964	Mtm1y/-_UP
Slc25a24	1.82190645	0.0007367	Mtm1y/-_UP
Krt77	1.82277925	0.03098856	Mtm1y/-_UP
Snx4	1.82348448	0.01241385	Mtm1y/-_UP
Efemp2	1.84885042	0.00775603	Mtm1y/-_UP
Cd93	1.8513715	0.00208361	Mtm1y/-_UP
Pbxip1	1.85444188	0.00020823	Mtm1y/-_UP
Htatip2	1.85521223	0.00084105	Mtm1y/-_UP
Homer2	1.86373781	0.017533	Mtm1y/-_UP
Hmcn2	1.86661675	0.00105906	Mtm1y/-_UP
Bves	1.87051941	0.00520904	Mtm1y/-_UP
Myoc	1.87859487	0.01665419	Mtm1y/-_UP
Mgl2	1.88070507	0.0190986	Mtm1y/-_UP
Pvalb	1.88256295	0.01348706	Mtm1y/-_UP
Mmp2	1.88271119	0.00980885	Mtm1y/-_UP
Atp1b4	1.90041517	0.00239301	Mtm1y/-_UP
Gpc4	1.91493906	0.00014747	Mtm1y/-_UP
Cotl1	1.9223175	0.01051887	Mtm1y/-_UP
Mustn1	1.92749951	0.01072829	Mtm1y/-_UP
F13b	1.93232394	0.01156646	Mtm1y/-_UP
Trim35	1.93545386	0.0075836	Mtm1y/-_UP
S100a13	1.9524323	0.01759689	Mtm1y/-_UP
Rnase4	1.96139469	0.03977552	Mtm1y/-_UP
Coa5	1.96584196	0.03410725	Mtm1y/-_UP
Cst6	2.00794858	0.02313052	Mtm1y/-_UP
Cfp	2.01631491	0.00182998	Mtm1y/-_UP
Igkc	2.02404224	0.00523129	Mtm1y/-_UP
Rrad	2.02472882	0.00342091	Mtm1y/-_UP
Pld3	2.02560844	0.00105906	Mtm1y/-_UP
Sppl2a	2.04666515	0.02441758	Mtm1y/-_UP
Tubb6	2.06216701	0.0008734	Mtm1y/-_UP

Ccn1	2.0774102	0.01044491	Mtm1y/-_UP
Ccdc124	2.078657	0.03353802	Mtm1y/-_UP
Emb	2.10832164	0.02198061	Mtm1y/-_UP
Atp1b2	2.13046209	0.00114512	Mtm1y/-_UP
Gpx8	2.1359985	0.00131135	Mtm1y/-_UP
Sirpa	2.14305088	0.00438691	Mtm1y/-_UP
Klk8	2.16472709	0.01934597	Mtm1y/-_UP
Srpx2	2.16536796	0.02328049	Mtm1y/-_UP
Aebp1	2.18860167	0.00011985	Mtm1y/-_UP
Man1b1	2.19125674	0.02528407	Mtm1y/-_UP
Tgfbr1	2.19406654	0.02119231	Mtm1y/-_UP
Tgm5	2.2054016	0.03139357	Mtm1y/-_UP
Sprr1a	2.22096429	0.0471571	Mtm1y/-_UP
Myh3	2.23673321	0.0156417	Mtm1y/-_UP
Cacng1	2.33130294	0.0007367	Mtm1y/-_UP
Maged2	2.34243233	0.00266057	Mtm1y/-_UP
Map2k1	2.34783287	0.0441642	Mtm1y/-_UP
Mfap4	2.35071452	0.02669682	Mtm1y/-_UP
Dapk2	2.35924001	0.00987415	Mtm1y/-_UP
App	2.36650526	0.00083054	Mtm1y/-_UP
Pdlim3	2.38106436	0.0002145	Mtm1y/-_UP
Aldh3b2	2.38923812	0.02387853	Mtm1y/-_UP
Ighg	2.3928067	0.01212165	Mtm1y/-_UP
Capns2	2.39580238	0.01069212	Mtm1y/-_UP
H2-Ab1	2.41238358	0.0014059	Mtm1y/-_UP
Sfrp1	2.43270628	0.02119231	Mtm1y/-_UP
Igkv12-41	2.4352205	0.00781883	Mtm1y/-_UP
Siglec1	2.44156137	0.03352086	Mtm1y/-_UP
Csrp3	2.45688195	2.69E-05	Mtm1y/-_UP
Cdc42	2.49194873	0.00083054	Mtm1y/-_UP
Anxa1	2.5119455	0.00020823	Mtm1y/-_UP
Nagpa	2.52356015	0.03991485	Mtm1y/-_UP

Angptl7	2.58178428	0.00697713	Mtm1y/-_UP
Ncam1	2.58657789	0.00276198	Mtm1y/-_UP
Apcs	2.5996147	0.0074526	Mtm1y/-_UP
Myh4	2.63025019	0.00239301	Mtm1y/-_UP
Postn	2.6574028	0.00497276	Mtm1y/-_UP
Tspo	2.66903537	0.00029737	Mtm1y/-_UP
Mgat2	2.68083723	0.00175179	Mtm1y/-_UP
Gsn	2.68182176	0.00529698	Mtm1y/-_UP
Angptl2	2.71760198	0.00266057	Mtm1y/-_UP
Cfhr2	2.71904913	0.00235121	Mtm1y/-_UP
Myo18a	2.75479451	0.02984739	Mtm1y/-_UP
Glipr2	2.76456158	0.00083054	Mtm1y/-_UP
Cilp	2.79526721	0.00105906	Mtm1y/-_UP
Igsf1	2.79819495	0.02519805	Mtm1y/-_UP
Stim2	2.81454443	0.01261633	Mtm1y/-_UP
Casp3	2.81840127	0.00036027	Mtm1y/-_UP
Rac2	2.85069179	0.0170995	Mtm1y/-_UP
Gabarapl1	2.85195725	0.03423471	Mtm1y/-_UP
Fabp1	2.85535715	0.02325362	Mtm1y/-_UP
Nes	2.87632371	0.02048988	Mtm1y/-_UP
Serpinb1a	2.93502788	2.70E-05	Mtm1y/-_UP
Loxl1	2.97212536	0.00175179	Mtm1y/-_UP
9530068E07Rik	3.06487382	0.00214749	Mtm1y/-_UP
Rab31	3.0719936	0.00900481	Mtm1y/-_UP
Gsn	3.12030416	5.88E-05	Mtm1y/-_UP
Fcgr2b	3.16064412	0.00532202	Mtm1y/-_UP
Cd1d1	3.19133072	0.00862642	Mtm1y/-_UP
Fbxo25	3.23054798	8.24E-06	Mtm1y/-_UP
Gtf2a1	3.2500032	0.01154283	Mtm1y/-_UP
Tnfrsf23	3.4156461	0.00022464	Mtm1y/-_UP
S100a4	3.4365655	0.00021304	Mtm1y/-_UP
Cpne2	3.48704933	2.70E-05	Mtm1y/-_UP

Ugt2b1	3.50953461	0.00266057	Mtm1y/-_UP
Gosr2	3.51755199	0.01418257	Mtm1y/-_UP
Lemd3	3.52561782	0.00020823	Mtm1y/-_UP
Mybph	3.69587495	0.01686942	Mtm1y/-_UP
Cx3cl1	3.73981817	0.00015212	Mtm1y/-_UP
Obsl1	3.96334151	5.35E-05	Mtm1y/-_UP
Mmgt1	3.98574157	0.00267847	Mtm1y/-_UP
Igkv6-13	4.01202402	0.00835232	Mtm1y/-_UP
Basp1	4.10615588	0.03833509	Mtm1y/-_UP
Ankrd1	4.20394444	0.01014297	Mtm1y/-_UP
Sesn1	4.26798052	5.35E-05	Mtm1y/-_UP
Cd99	4.44543674	0.01609468	Mtm1y/-_UP
Hjv	4.63800663	9.44E-08	Mtm1y/-_UP
Tnnt3	4.71607945	0.00139121	Mtm1y/-_UP

Table S4: Mouse global untargeted proteomics analysis where Mtm1$^{y/-}$ with mava treatment are compared to Mtm1$^{y/-}$ with vehicle.

protein_identifier	log2FC	padj	direction
Psmd12	4.58448677	0.00073584	Mtm1y/-_mava_UP
Rbm3	4.58013427	0.00327293	Mtm1y/-_mava_UP
Kcnq5	4.27552284	0.00073584	Mtm1y/-_mava_UP
Ankrd1	3.96600414	0.03287858	Mtm1y/-_mava_UP
Nagpa	3.89117963	0.02945975	Mtm1y/-_mava_UP
Tnnt3	3.88436677	0.00577795	Mtm1y/-_mava_UP
Snx13	3.54019354	0.0072178	Mtm1y/-_mava_UP
Dhx30	3.40008693	0.02379928	Mtm1y/-_mava_UP
H2-Aa	3.22269054	0.03896879	Mtm1y/-_mava_UP
Sesn1	3.11722752	0.04173617	Mtm1y/-_mava_UP
S100a4	3.07965591	0.00833012	Mtm1y/-_mava_UP
Fcgr2b	3.04937262	0.00327293	Mtm1y/-_mava_UP
Cd1d1	2.94933101	0.01201662	Mtm1y/-_mava_UP
Tnfrsf23	2.93770382	0.01101861	Mtm1y/-_mava_UP
Hax1	2.92250071	0.00159695	Mtm1y/-_mava_UP
Atg9b	2.91295329	0.00937694	Mtm1y/-_mava_UP
Anxa1	2.88490514	0.00095756	Mtm1y/-_mava_UP
1110025L11Rik	2.79352361	0.02945975	Mtm1y/-_mava_UP
Sphk1	2.73264967	0.01054988	Mtm1y/-_mava_UP
Cpne2	2.64217021	0.00147399	Mtm1y/-_mava_UP
Cdc42	2.63998109	0.0115834	Mtm1y/-_mava_UP
Apcs	2.60279576	0.03872253	Mtm1y/-_mava_UP
St6galnac4	2.54269035	0.01054988	Mtm1y/-_mava_UP
Nefm	2.53416126	0.00252465	Mtm1y/-_mava_UP
Cfhr2	2.50714253	0.00833012	Mtm1y/-_mava_UP
Bid	2.47599543	0.00183054	Mtm1y/-_mava_UP
Slc2a1	2.47302807	0.0231481	Mtm1y/-_mava_UP
Trio	2.46891125	0.0380044	Mtm1y/-_mava_UP
Abhd14a	2.45886182	0.03110528	Mtm1y/-_mava_UP

Ptpn4	2.41547643	0.0231481	Mtm1y/-_mava_UP
Gsn	2.41212096	0.00252465	Mtm1y/-_mava_UP
Ptprc	2.326849	0.03079169	Mtm1y/-_mava_UP
Mstn	2.32028359	0.03953035	Mtm1y/-_mava_UP
Ugdh	2.29942506	0.00993482	Mtm1y/-_mava_UP
Ighg2b	2.17750602	0.03732973	Mtm1y/-_mava_UP
Pdpn	2.15954519	0.02685	Mtm1y/-_mava_UP
Htra1	2.1058864	0.02928864	Mtm1y/-_mava_UP
Cilp	2.07684505	0.0046157	Mtm1y/-_mava_UP
Angptl2	2.05807492	0.00689157	Mtm1y/-_mava_UP
Icam1	2.03905425	0.00259342	Mtm1y/-_mava_UP
Alox8	2.03222507	0.0380044	Mtm1y/-_mava_UP
Ncam1	2.02393456	0.01413484	Mtm1y/-_mava_UP
Serpinb1a	1.99621246	0.00108197	Mtm1y/-_mava_UP
Dapk2	1.90098824	0.04873443	Mtm1y/-_mava_UP
Fst	1.90010578	0.01395387	Mtm1y/-_mava_UP
Sumo2	1.88288327	0.01250683	Mtm1y/-_mava_UP
Hmcn2	1.84349433	0.00743984	Mtm1y/-_mava_UP
Mfap4	1.83520039	0.02316214	Mtm1y/-_mava_UP
Gsn	1.82751947	0.00351213	Mtm1y/-_mava_UP
App	1.81704652	0.0072178	Mtm1y/-_mava_UP
Postn	1.76720483	0.04173617	Mtm1y/-_mava_UP
Myoc	1.72736653	0.02580589	Mtm1y/-_mava_UP
Gpc4	1.71819276	0.00141591	Mtm1y/-_mava_UP
Mrc2	1.71113083	0.04367501	Mtm1y/-_mava_UP
Fbln5	1.70700682	0.00804264	Mtm1y/-_mava_UP
Gpc1	1.69748726	0.00159695	Mtm1y/-_mava_UP
Clec3b	1.68391319	0.03719104	Mtm1y/-_mava_UP
Fbln2	1.68068227	0.00272289	Mtm1y/-_mava_UP
Pvalb	1.64015482	0.00247626	Mtm1y/-_mava_UP
Sirpa	1.63498854	0.03079169	Mtm1y/-_mava_UP
Tspan13	1.62480473	0.01682057	Mtm1y/-_mava_UP

Tubb4a	1.57309341	0.04467349	Mtm1y/-_mava_UP
Cacng1	1.558586	0.00252465	Mtm1y/-_mava_UP
Igf2	1.5585495	0.03841067	Mtm1y/-_mava_UP
Csrp3	1.55761263	0.00138515	Mtm1y/-_mava_UP
Adgre5	1.55669166	0.01035752	Mtm1y/-_mava_UP
Galnt7	1.5259334	0.00554392	Mtm1y/-_mava_UP
Anpep	1.49629276	0.01152397	Mtm1y/-_mava_UP
Aebp1	1.49329036	0.04784845	Mtm1y/-_mava_UP
Pld3	1.49258983	0.01249066	Mtm1y/-_mava_UP
Idua	1.48387219	0.02685	Mtm1y/-_mava_UP
Tmem43	1.48284585	0.00095756	Mtm1y/-_mava_UP
Ftl1	1.45705157	0.0115834	Mtm1y/-_mava_UP
Mlf2	1.44028048	0.03616541	Mtm1y/-_mava_UP
Sod3	1.43194742	0.00764202	Mtm1y/-_mava_UP
Acbd4	1.4171112	0.01208861	Mtm1y/-_mava_UP
Ighv1-31	1.4060764	0.04199433	Mtm1y/-_mava_UP
Lrrn1	1.35600401	0.02580589	Mtm1y/-_mava_UP
Rrad	1.34151851	0.0466877	Mtm1y/-_mava_UP
Cd55	1.31624255	0.0093814	Mtm1y/-_mava_UP
Sort1	1.28572175	0.02399908	Mtm1y/-_mava_UP
Igf1r	1.27527774	0.04212742	Mtm1y/-_mava_UP
Slc38a2	1.24082147	0.04270581	Mtm1y/-_mava_UP
F13a1	1.23312749	0.01826273	Mtm1y/-_mava_UP
Cspg4	1.22751937	0.03841067	Mtm1y/-_mava_UP
Trrap	1.22084072	0.03423478	Mtm1y/-_mava_UP
Egf	1.20005188	0.03682983	Mtm1y/-_mava_UP
Hrg	1.19381007	0.01208861	Mtm1y/-_mava_UP
S100a13	1.18431547	0.03590132	Mtm1y/-_mava_UP
Fbln1	1.16808302	0.02702681	Mtm1y/-_mava_UP
P2rx4	1.1659419	0.00950415	Mtm1y/-_mava_UP
Efemp1	1.1612794	0.01255472	Mtm1y/-_mava_UP
Cd302	1.16050048	0.01083021	Mtm1y/-_mava_UP

Camk2d	1.1592291	0.00259342	Mtm1y/-_mava_UP
Rer1	1.15699898	0.00592465	Mtm1y/-_mava_UP
Mrc1	1.15478652	0.03110528	Mtm1y/-_mava_UP
Zbed5	1.15290182	0.03241036	Mtm1y/-_mava_UP
Cpd	1.13790571	0.01248175	Mtm1y/-_mava_UP
Pdlim3	1.13350582	0.01575707	Mtm1y/-_mava_UP
Fn1	1.12722652	0.03399526	Mtm1y/-_mava_UP
Man1a	1.11536204	0.03938264	Mtm1y/-_mava_UP
Cyb5r3	1.11327549	0.00247626	Mtm1y/-_mava_UP
Aspn	1.11290219	0.01248175	Mtm1y/-_mava_UP
Atp1b2	1.11118021	0.0306521	Mtm1y/-_mava_UP
Pbxip1	1.10025322	0.01208861	Mtm1y/-_mava_UP
Galnt1	1.09215759	0.04400264	Mtm1y/-_mava_UP
Chrnb1	1.09048347	0.00263722	Mtm1y/-_mava_UP
Zfpl1	1.08342855	0.04200771	Mtm1y/-_mava_UP
Cd82	1.07550427	0.01248175	Mtm1y/-_mava_UP
Cfh	1.06472847	0.03938264	Mtm1y/-_mava_UP
Smoc2	1.06305616	0.03399526	Mtm1y/-_mava_UP
Ckap4	1.05681542	0.03399526	Mtm1y/-_mava_UP
Pycr1	1.05493077	0.0180655	Mtm1y/-_mava_UP
Plxnb2	1.03365601	0.01094496	Mtm1y/-_mava_UP
Gpx3	1.03313982	0.02922444	Mtm1y/-_mava_UP
Api5	1.03309839	0.00950415	Mtm1y/-_mava_UP
Anxa7	1.0156852	0.0128913	Mtm1y/-_mava_UP
Cybc1	1.00117213	0.02722259	Mtm1y/-_mava_UP
Ints3	0.99745888	0.04849993	Mtm1y/-_mava_UP
Cfd	0.99121474	0.0380044	Mtm1y/-_mava_UP
Mtdh	0.98756483	0.04985887	Mtm1y/-_mava_UP
Cstb	0.98182859	0.0380044	Mtm1y/-_mava_UP
Shisa4	0.97514332	0.02580589	Mtm1y/-_mava_UP
Csgalnact2	0.9712431	0.01248175	Mtm1y/-_mava_UP
Ahsg	0.96815117	0.00832906	Mtm1y/-_mava_UP

Ehbp1l1	0.96420895	0.02945975	Mtm1y/-_mava_UP
Pon2	0.96046655	0.00247626	Mtm1y/-_mava_UP
Acot9	0.95566699	0.00360984	Mtm1y/-_mava_UP
Ppib	0.9555109	0.0291608	Mtm1y/-_mava_UP
Serpinb6a	0.94939989	0.01115596	Mtm1y/-_mava_UP
Slc25a24	0.94546818	0.01618993	Mtm1y/-_mava_UP
Esyt1	0.94165765	0.00804264	Mtm1y/-_mava_UP
Sigmar1	0.93753165	0.01350456	Mtm1y/-_mava_UP
Mix23	0.92717754	0.04763086	Mtm1y/-_mava_UP
Pgrmc2	0.92077237	0.01582179	Mtm1y/-_mava_UP
Itm2b	0.91731065	0.01248175	Mtm1y/-_mava_UP
Anxa4	0.91686529	0.01198306	Mtm1y/-_mava_UP
Hprt1	0.90362724	0.02922444	Mtm1y/-_mava_UP
Mrpl12	0.90092233	0.02593608	Mtm1y/-_mava_UP
Itga5	0.88737128	0.03075888	Mtm1y/-_mava_UP
Lgals1	0.87468084	0.02838532	Mtm1y/-_mava_UP
Man1a2	0.86484409	0.03953035	Mtm1y/-_mava_UP
Tm9sf3	0.85584704	0.03399526	Mtm1y/-_mava_UP
Cst3	0.85046675	0.01522854	Mtm1y/-_mava_UP
Ergic1	0.84043861	0.0128913	Mtm1y/-_mava_UP
Myl4	0.83876501	0.03286073	Mtm1y/-_mava_UP
Ptgfrn	0.82542179	0.02722259	Mtm1y/-_mava_UP
Esyt2	0.82014423	0.00833012	Mtm1y/-_mava_UP
Dusp29	0.81411698	0.04427036	Mtm1y/-_mava_UP
Txn2	0.80778471	0.04640974	Mtm1y/-_mava_UP
Galnt2	0.8045045	0.01738698	Mtm1y/-_mava_UP
Epb41l2	0.80253248	0.01250683	Mtm1y/-_mava_UP
Camk2a	0.7991676	0.00247626	Mtm1y/-_mava_UP
B3galnt2	0.79326105	0.02580589	Mtm1y/-_mava_UP
Lamtor1	0.79312368	0.01522854	Mtm1y/-_mava_UP
Lman2	0.79217276	0.00950415	Mtm1y/-_mava_UP
Cpt1a	0.78852693	0.03132115	Mtm1y/-_mava_UP

Tmed5	0.78325783	0.00095756	Mtm1y/-_mava_UP
Pdlim7	0.78265034	0.02722259	Mtm1y/-_mava_UP
Rnf121	0.77079129	0.02580589	Mtm1y/-_mava_UP
Ucp3	0.76837802	0.04205609	Mtm1y/-_mava_UP
Nenf	0.74439396	0.03719104	Mtm1y/-_mava_UP
Ncbp1	0.74363654	0.04173617	Mtm1y/-_mava_UP
Wls	0.7325584	0.0380044	Mtm1y/-_mava_UP
Erlin1	0.72566368	0.032724	Mtm1y/-_mava_UP
Cyp20a1	0.72534973	0.04784845	Mtm1y/-_mava_UP
Letmd1	0.71306615	0.02726272	Mtm1y/-_mava_UP
Kyat3	0.71201812	0.0334782	Mtm1y/-_mava_UP
Calu	0.70820257	0.03399526	Mtm1y/-_mava_UP
Sel1l	0.70280043	0.0306521	Mtm1y/-_mava_UP
Igf2r	0.70163905	0.03179303	Mtm1y/-_mava_UP
Rras	0.70014735	0.01878399	Mtm1y/-_mava_UP
Cyb5a	0.69812305	0.0380044	Mtm1y/-_mava_UP
Anxa2	0.69357958	0.03605885	Mtm1y/-_mava_UP
Prnp	0.69324986	0.04771466	Mtm1y/-_mava_UP
Yme1l1	0.6821125	0.032724	Mtm1y/-_mava_UP
Spcs1	0.68091009	0.03286073	Mtm1y/-_mava_UP
Cd59a	0.67777157	0.02351433	Mtm1y/-_mava_UP
Sdf4	0.67748269	0.032724	Mtm1y/-_mava_UP
Arhgap1	0.67556161	0.04917648	Mtm1y/-_mava_UP
Vwa5a	0.67436013	0.04173617	Mtm1y/-_mava_UP
Nucb1	0.67336746	0.00967766	Mtm1y/-_mava_UP
Spcs2	0.67239383	0.03731563	Mtm1y/-_mava_UP
Man2a2	0.67211074	0.03286073	Mtm1y/-_mava_UP
Tnxb	0.66573903	0.03953035	Mtm1y/-_mava_UP
Apoa4	0.66145005	0.03244114	Mtm1y/-_mava_UP
Tm9sf2	0.65992078	0.032724	Mtm1y/-_mava_UP
Itga7	0.65749932	0.03731563	Mtm1y/-_mava_UP
Jsrp1	0.64278382	0.01250683	Mtm1y/-_mava_UP

Atp6v0d1	0.64017815	0.03132115	Mtm1y/-_mava_UP
Stt3a	0.63811936	0.02238831	Mtm1y/-_mava_UP
Cdh13	0.63779769	0.03710251	Mtm1y/-_mava_UP
Aldh4a1	0.63752715	0.03082845	Mtm1y/-_mava_UP
Pgam5	0.6348368	0.04985887	Mtm1y/-_mava_UP
Lamp1	0.63367023	0.03079169	Mtm1y/-_mava_UP
Ergic2	0.63313685	0.02495272	Mtm1y/-_mava_UP
Mrpl48	0.63218696	0.02196788	Mtm1y/-_mava_UP
Anxa11	0.63071791	0.02278029	Mtm1y/-_mava_UP
Ociad2	0.62479693	0.04173617	Mtm1y/-_mava_UP
Pon3	0.62442446	0.01717573	Mtm1y/-_mava_UP
Aldh2	0.62258672	0.01115596	Mtm1y/-_mava_UP
Rapsn	0.61584803	0.03079169	Mtm1y/-_mava_UP
Tpt1	0.61262409	0.02673023	Mtm1y/-_mava_UP
Hsd17b12	0.61036031	0.04047612	Mtm1y/-_mava_UP
Mcat	0.60298622	0.04167129	Mtm1y/-_mava_UP
Slc3a2	0.60091933	0.03079169	Mtm1y/-_mava_UP
Prkca	0.58696062	0.03365849	Mtm1y/-_mava_UP
Atp6v0a1	0.58448245	0.01208193	Mtm1y/-_mava_UP
Cyp27a1	0.58308177	0.00327293	Mtm1y/-_mava_UP
Rcn2	0.57727615	0.04985887	Mtm1y/-_mava_UP
Cemip2	0.5762159	0.01035752	Mtm1y/-_mava_UP
Adpgk	0.57145898	0.02884408	Mtm1y/-_mava_UP
Tmed9	0.56954241	0.04173617	Mtm1y/-_mava_UP
Lman2l	0.5660261	0.02945975	Mtm1y/-_mava_UP
Mcu	0.55650285	0.00327293	Mtm1y/-_mava_UP
Trmu	0.55589469	0.03399526	Mtm1y/-_mava_UP
Bcam	0.55175218	0.0302593	Mtm1y/-_mava_UP
Coq3	0.54947291	0.03655078	Mtm1y/-_mava_UP
Micu1	0.54692648	0.00863765	Mtm1y/-_mava_UP
Fech	0.53921187	0.01826273	Mtm1y/-_mava_UP
Mogs	0.51769402	0.03179303	Mtm1y/-_mava_UP

Ssr4	0.51339691	0.04047612	Mtm1y/-_mava_UP
Alg9	0.51276766	0.02945975	Mtm1y/-_mava_UP
Oxa1l	0.49133946	0.03110528	Mtm1y/-_mava_UP
Mrpl18	0.48163006	0.02928864	Mtm1y/-_mava_UP
Rab7a	0.4798153	0.04889854	Mtm1y/-_mava_UP
Tmx3	0.47602971	0.0380044	Mtm1y/-_mava_UP
Gadd45gip1	0.47110556	0.04400264	Mtm1y/-_mava_UP
Sypl1	0.46697394	0.02838532	Mtm1y/-_mava_UP
Cox7a2l	0.46541296	0.01878399	Mtm1y/-_mava_UP
Creld2	0.46365632	0.03953035	Mtm1y/-_mava_UP
Tmed4	0.46172677	0.04973251	Mtm1y/-_mava_UP
Asph	0.45611582	0.03287858	Mtm1y/-_mava_UP
Insr	0.45228684	0.011056	Mtm1y/-_mava_UP
Bpnt2	0.4512639	0.04934686	Mtm1y/-_mava_UP
Pisd	0.4491727	0.01054988	Mtm1y/-_mava_UP
Vwa8	0.44478596	0.04212742	Mtm1y/-_mava_UP
Hars2	0.44414484	0.03953035	Mtm1y/-_mava_UP
Coq5	0.44396984	0.03841067	Mtm1y/-_mava_UP
Exoc6	0.4389292	0.04089006	Mtm1y/-_mava_UP
Trmt10c	0.42923337	0.04244608	Mtm1y/-_mava_UP
Pmpcb	0.42787765	0.01922885	Mtm1y/-_mava_UP
Serhl	0.42539948	0.04047612	Mtm1y/-_mava_UP
Lap3	0.42414544	0.02981121	Mtm1y/-_mava_UP
Atp6v1a	0.41137067	0.04431824	Mtm1y/-_mava_UP
Tmx1	0.39673093	0.02206077	Mtm1y/-_mava_UP
Glud1	0.38310847	0.03841067	Mtm1y/-_mava_UP
Emc7	0.37868029	0.04771466	Mtm1y/-_mava_UP
Apmap	0.37308919	0.03423478	Mtm1y/-_mava_UP
Surf1	0.37253189	0.04528368	Mtm1y/-_mava_UP
Tor1aip2	0.35371262	0.04173617	Mtm1y/-_mava_UP
Afg3l1	0.34966798	0.04346959	Mtm1y/-_mava_UP
Oma1	0.3406843	0.0442381	Mtm1y/-_mava_UP

Dnaja3	0.31232132	0.03120039	Mtm1y/-_mava_UP
Rhot1	0.26007614	0.03421723	Mtm1y/-_mava_UP
Ndufaf1	0.25135908	0.03938264	Mtm1y/-_mava_UP
Mtch1	0.24940051	0.03682364	Mtm1y/-_mava_UP
Dhrs7c	-0.3348381	0.03286073	Mtm1y/-_mava_DOWN
Npepps	-0.3432593	0.04173617	Mtm1y/-_mava_DOWN
Ak1	-0.3441734	0.02399059	Mtm1y/-_mava_DOWN
Ndufa11	-0.3574915	0.03731563	Mtm1y/-_mava_DOWN
Map2k3	-0.365489	0.03719104	Mtm1y/-_mava_DOWN
Adss1	-0.4184988	0.04491479	Mtm1y/-_mava_DOWN
Nnt	-0.4345337	0.04763086	Mtm1y/-_mava_DOWN
Aifm2	-0.4448066	0.01563767	Mtm1y/-_mava_DOWN
Gars1	-0.4838991	0.04047612	Mtm1y/-_mava_DOWN
Scn4b	-0.5093292	0.01325034	Mtm1y/-_mava_DOWN
Actn4	-0.5106431	0.01870792	Mtm1y/-_mava_DOWN
Mpi	-0.5311658	0.04985887	Mtm1y/-_mava_DOWN
Eef1a2	-0.5323937	0.02495272	Mtm1y/-_mava_DOWN
Wdr46	-0.5337958	0.03079169	Mtm1y/-_mava_DOWN
Fastkd5	-0.535516	0.02847494	Mtm1y/-_mava_DOWN
Cct7	-0.5486206	0.04784845	Mtm1y/-_mava_DOWN
Ppm1b	-0.5614673	0.01826273	Mtm1y/-_mava_DOWN
Hspb6	-0.569939	0.00252465	Mtm1y/-_mava_DOWN
Mief2	-0.573388	0.00554392	Mtm1y/-_mava_DOWN
Uba1	-0.5828556	0.00804264	Mtm1y/-_mava_DOWN
Gstt2	-0.5909551	0.03648723	Mtm1y/-_mava_DOWN
Dnpep	-0.5948549	0.01110058	Mtm1y/-_mava_DOWN
Pfkm	-0.6058917	0.04522551	Mtm1y/-_mava_DOWN
Slc16a1	-0.6100093	0.00073584	Mtm1y/-_mava_DOWN
Capza2	-0.6137933	0.01201662	Mtm1y/-_mava_DOWN
Ephx2	-0.6170597	0.01951477	Mtm1y/-_mava_DOWN
Rap1gds1	-0.6297301	0.01194908	Mtm1y/-_mava_DOWN
Ppp2r1a	-0.6320575	0.01835584	Mtm1y/-_mava_DOWN

Smpdl3b	-0.6393718	0.03319056	Mtm1y/-_mava_DOWN
Ppp2ca	-0.6472522	0.04047612	Mtm1y/-_mava_DOWN
Eloc	-0.6479663	0.02837548	Mtm1y/-_mava_DOWN
Cul5	-0.6565609	0.04047612	Mtm1y/-_mava_DOWN
Ryr1	-0.6586845	0.00833012	Mtm1y/-_mava_DOWN
Pura	-0.6622837	0.00252465	Mtm1y/-_mava_DOWN
Pfkfb1	-0.6630795	0.0180655	Mtm1y/-_mava_DOWN
Psmd2	-0.6655308	0.032724	Mtm1y/-_mava_DOWN
Prdx1	-0.6732722	0.03079169	Mtm1y/-_mava_DOWN
Rnf123	-0.6915451	0.0072178	Mtm1y/-_mava_DOWN
Dhrs11	-0.6957445	0.02928864	Mtm1y/-_mava_DOWN
Prkg1	-0.7092293	0.03287858	Mtm1y/-_mava_DOWN
Asrgl1	-0.7098491	0.01035752	Mtm1y/-_mava_DOWN
Twf2	-0.7131016	0.0442381	Mtm1y/-_mava_DOWN
Mavs	-0.7335987	0.03110528	Mtm1y/-_mava_DOWN
Abca7	-0.738151	0.02945975	Mtm1y/-_mava_DOWN
Cenpv	-0.7387993	0.03086677	Mtm1y/-_mava_DOWN
Fkbp11	-0.7393931	0.01595423	Mtm1y/-_mava_DOWN
Prxl2a	-0.7504252	0.00536538	Mtm1y/-_mava_DOWN
Mylk2	-0.7537556	0.01529903	Mtm1y/-_mava_DOWN
Washc5	-0.7539384	0.00360984	Mtm1y/-_mava_DOWN
Inpp4b	-0.7579023	0.04173617	Mtm1y/-_mava_DOWN
Kcna7	-0.7586826	0.02280079	Mtm1y/-_mava_DOWN
Gbe1	-0.7599453	0.01859812	Mtm1y/-_mava_DOWN
Scrn3	-0.7646996	0.02280079	Mtm1y/-_mava_DOWN
Dnm1l	-0.7807132	0.0072178	Mtm1y/-_mava_DOWN
Txnrd1	-0.7872077	0.02206077	Mtm1y/-_mava_DOWN
Tpp2	-0.7939521	0.04183761	Mtm1y/-_mava_DOWN
Cct2	-0.7965501	0.03655078	Mtm1y/-_mava_DOWN
Pgp	-0.8263726	0.03181426	Mtm1y/-_mava_DOWN
Ppp2r2a	-0.8353573	0.01899912	Mtm1y/-_mava_DOWN
Ampd1	-0.8361529	0.03399526	Mtm1y/-_mava_DOWN

Paics	-0.8417737	0.02945975	Mtm1y/-_mava_DOWN
Phyhd1	-0.8423785	0.03901508	Mtm1y/-_mava_DOWN
Cops6	-0.8500049	0.03088294	Mtm1y/-_mava_DOWN
Kcnma1	-0.8629559	0.00354661	Mtm1y/-_mava_DOWN
Nt5c2	-0.863373	0.02945975	Mtm1y/-_mava_DOWN
Stac3	-0.867111	0.02270684	Mtm1y/-_mava_DOWN
Man2c1	-0.8721401	0.00950415	Mtm1y/-_mava_DOWN
Pycr3	-0.8792562	0.02047625	Mtm1y/-_mava_DOWN
Mdh1	-0.8793871	0.04173617	Mtm1y/-_mava_DOWN
Tmod4	-0.8849583	0.00252465	Mtm1y/-_mava_DOWN
Stradb	-0.8911723	0.03399526	Mtm1y/-_mava_DOWN
Usp15	-0.8968671	0.00776658	Mtm1y/-_mava_DOWN
Slc25a13	-0.9072967	0.00950415	Mtm1y/-_mava_DOWN
Psmb6	-0.9336085	0.00950415	Mtm1y/-_mava_DOWN
Acot11	-0.9457304	0.00259342	Mtm1y/-_mava_DOWN
Lrrc38	-0.9476772	0.03432218	Mtm1y/-_mava_DOWN
Asb2	-0.9500473	0.00833012	Mtm1y/-_mava_DOWN
Phkb	-0.9529516	0.01035752	Mtm1y/-_mava_DOWN
Coro6	-0.9642634	0.010434	Mtm1y/-_mava_DOWN
Sh3bgr	-0.9646561	0.00950415	Mtm1y/-_mava_DOWN
Cops5	-0.9721394	0.01208861	Mtm1y/-_mava_DOWN
Zer1	-0.985772	0.03286073	Mtm1y/-_mava_DOWN
Ubac1	-0.9890611	0.04173617	Mtm1y/-_mava_DOWN
Fn3k	-1.0063893	0.00833012	Mtm1y/-_mava_DOWN
Hspb2	-1.0087517	0.01093519	Mtm1y/-_mava_DOWN
Cops3	-1.0104566	0.01152397	Mtm1y/-_mava_DOWN
Fhl3	-1.0150386	0.00950415	Mtm1y/-_mava_DOWN
Cops4	-1.0169514	0.01248175	Mtm1y/-_mava_DOWN
Pdlim5	-1.0177285	0.01054988	Mtm1y/-_mava_DOWN
Vcp	-1.0232826	0.02838532	Mtm1y/-_mava_DOWN
Cops8	-1.0261863	0.00584585	Mtm1y/-_mava_DOWN
Fabp3	-1.0406891	0.0437162	Mtm1y/-_mava_DOWN

Npepl1	-1.0481743	0.02722259	Mtm1y/-_mava_DOWN
Bag2	-1.0660885	0.02928864	Mtm1y/-_mava_DOWN
Alpk2	-1.0672718	0.01595423	Mtm1y/-_mava_DOWN
Gstm7	-1.0881559	0.02316214	Mtm1y/-_mava_DOWN
Pdlim5	-1.1045952	0.02673023	Mtm1y/-_mava_DOWN
Actn2	-1.1391294	0.01248175	Mtm1y/-_mava_DOWN
Ldhb	-1.1399933	0.0302593	Mtm1y/-_mava_DOWN
Ces1d	-1.1569382	0.03497321	Mtm1y/-_mava_DOWN
Gnpda1	-1.1608801	0.01522854	Mtm1y/-_mava_DOWN
Tmod1	-1.1865785	0.03181426	Mtm1y/-_mava_DOWN
Nat14	-1.2149064	0.03682364	Mtm1y/-_mava_DOWN
Pm20d2	-1.2327675	0.01035752	Mtm1y/-_mava_DOWN
Myoz2	-1.2461814	0.03698361	Mtm1y/-_mava_DOWN
Klhl34	-1.2527393	0.00950415	Mtm1y/-_mava_DOWN
Lrrc30	-1.2604287	0.02141517	Mtm1y/-_mava_DOWN
Vwa1	-1.2624587	0.02155407	Mtm1y/-_mava_DOWN
Gsta4	-1.2735164	0.03399526	Mtm1y/-_mava_DOWN
Syp	-1.2893228	0.02677563	Mtm1y/-_mava_DOWN
Nt5c1a	-1.3048855	0.00147399	Mtm1y/-_mava_DOWN
Myl3	-1.3074849	0.0180655	Mtm1y/-_mava_DOWN
Col6a6	-1.3079085	0.0466877	Mtm1y/-_mava_DOWN
Alad	-1.3125715	0.00906033	Mtm1y/-_mava_DOWN
Grb14	-1.3235143	0.02413123	Mtm1y/-_mava_DOWN
Cnot9	-1.3569296	0.03079169	Mtm1y/-_mava_DOWN
H2az1	-1.3607109	0.03938264	Mtm1y/-_mava_DOWN
Myom3	-1.3627411	0.01563767	Mtm1y/-_mava_DOWN
Oplah	-1.3692418	0.00147399	Mtm1y/-_mava_DOWN
Xdh	-1.3758719	0.04173617	Mtm1y/-_mava_DOWN
Cped1	-1.3863998	0.00095756	Mtm1y/-_mava_DOWN
Mybpc1	-1.3883842	0.00192989	Mtm1y/-_mava_DOWN
Ugp2	-1.3891676	0.00119838	Mtm1y/-_mava_DOWN
Ttn	-1.396438	0.00204997	Mtm1y/-_mava_DOWN

Aqp7	-1.4175692	0.03267914	Mtm1y/-_mava_DOWN
Lsmem1	-1.4467675	0.0072178	Mtm1y/-_mava_DOWN
Tpm3	-1.5133516	0.03953035	Mtm1y/-_mava_DOWN
Nrbp1	-1.5167809	0.03953035	Mtm1y/-_mava_DOWN
Map2k6	-1.5482023	0.00982993	Mtm1y/-_mava_DOWN
Atp2a2	-1.5864356	0.01208193	Mtm1y/-_mava_DOWN
Srrm2	-1.595482	0.03286073	Mtm1y/-_mava_DOWN
Atp5mc2	-1.6322999	0.04047612	Mtm1y/-_mava_DOWN
Atp2a2	-1.6919424	0.03399526	Mtm1y/-_mava_DOWN
Rspo3	-1.7062566	0.04173617	Mtm1y/-_mava_DOWN
Erfe	-1.7344053	0.00095756	Mtm1y/-_mava_DOWN
Myh6	-1.895332	0.01110058	Mtm1y/-_mava_DOWN
Lrrc39	-1.9294294	0.01250683	Mtm1y/-_mava_DOWN
Apobec2	-2.0221794	0.00095756	Mtm1y/-_mava_DOWN
Myl10	-2.0405972	0.03731563	Mtm1y/-_mava_DOWN
Zfp174	-2.0526846	0.02046927	Mtm1y/-_mava_DOWN
Mgst1	-2.068683	0.00095756	Mtm1y/-_mava_DOWN
Ppat	-2.0808673	0.00252465	Mtm1y/-_mava_DOWN
Bdh1	-2.109684	0.00568557	Mtm1y/-_mava_DOWN
Myh7	-2.5644221	0.00536538	Mtm1y/-_mava_DOWN
Myl2	-2.632382	0.00950415	Mtm1y/-_mava_DOWN
Map2k1	-2.6664633	0.04173617	Mtm1y/-_mava_DOWN
Tnnt1	-2.6797269	0.00250245	Mtm1y/-_mava_DOWN
Tnni1	-2.7446772	0.00833456	Mtm1y/-_mava_DOWN
Fxyd6	-2.7938585	0.04491479	Mtm1y/-_mava_DOWN
Myl2	-2.800589	0.00192983	Mtm1y/-_mava_DOWN
Selenop	-2.8875676	0.02945975	Mtm1y/-_mava_DOWN
Mtcl2	-3.2950653	0.03938264	Mtm1y/-_mava_DOWN
Myom1	-4.4294227	0.00095756	Mtm1y/-_mava_DOWN

Table S5: Mouse global untargeted proteomics analysis (Enrichment GO analysis with mava treatment)

Enrichment FDR	nGenes	Pathway Genes	Fold Enrichment	Pathway	direction
4.20E-10	20	423	7.41374394	GO:0003012 muscle system proc.	Maintained_MAVA
4.20E-10	18	312	8.63787375	GO:0006936 muscle contraction	Maintained_MAVA
1.77E-06	12	172	9.17107584	GO:0006941 striated muscle contraction	Maintained_MAVA
1.99E-05	23	3211	3.39002268	GO:0003008 system proc.	Maintained_MAVA
2.85E-05	11	249	7.82703886	GO:0003015 heart proc.	Maintained_MAVA
8.96E-05	9	131	9.28571429	GO:0060048 cardiac muscle contraction	Maintained_MAVA
0.00013369	10	237	7.45840505	GO:0060047 heart contraction	Maintained_MAVA
0.00015742	6	50	16.8831169	GO:0014888 striated muscle adaptation	Maintained_MAVA
0.00015742	6	69	16.8831169	GO:0055008 cardiac muscle tissue morphogenesis	Maintained_MAVA
0.00016799	13	518	5.02976191	GO:0008015 blood circulation	Maintained_MAVA
0.00026748	13	547	4.76190476	GO:0003013 circulatory system proc.	Maintained_MAVA
0.00026748	4	8	35.3741497	GO:0014883 transition between fast and slow fiber	Maintained_MAVA
0.00026748	6	80	14.8571429	GO:0060415 muscle tissue morphogenesis	Maintained_MAVA
0.0002771	5	29	20.6349206	GO:0002026 reg. of the force of heart contraction	Maintained_MAVA
0.0002771	6	88	14.2857143	GO:0048644 muscle organ morphogenesis	Maintained_MAVA

0.00034954	5	25	19.3452381	GO:0043501 skeletal muscle adaptation	Maintained_MAVA
0.00034954	5	53	19.3452381	GO:0055010 ventricular cardiac muscle tissue morphogenesis	Maintained_MAVA
0.00036845	6	45	13.2653061	GO:0003009 skeletal muscle contraction	Maintained_MAVA
0.00059836	5	63	17.1957672	GO:0003229 ventricular cardiac muscle tissue development	Maintained_MAVA
0.00061084	4	12	27.5132275	GO:0014733 reg. of skeletal muscle adaptation	Maintained_MAVA
0.00072562	5	77	16.2907268	GO:0003208 cardiac ventricle morphogenesis	Maintained_MAVA
0.00079593	6	59	11.2554113	GO:0050879 multicellular organismal movement	Maintained_MAVA
0.00079593	6	59	11.2554113	GO:0050881 musculoskeletal movement	Maintained_MAVA
0.00147204	8	247	6.6031746	GO:0048738 cardiac muscle tissue development	Maintained_MAVA
0.00189061	8	265	6.34920635	GO:0014706 striated muscle tissue development	Maintained_MAVA
0.00230927	7	163	7.34463277	GO:0006937 reg. of muscle contraction	Maintained_MAVA
0.00430925	3	7	30.952381	GO:0003010 voluntary skeletal muscle contraction	Maintained_MAVA
0.00430925	3	7	30.952381	GO:0014721 twitch skeletal muscle contraction	Maintained_MAVA
0.00430925	4	23	16.5079365	GO:0046040 IMP metabolic proc.	Maintained_MAVA
0.00430925	10	445	4.32900433	GO:0060537 muscle tissue development	Maintained_MAVA
0.00443236	5	135	10.6732348	GO:0003206 cardiac chamber morphogenesis	Maintained_MAVA
0.00590389	6	125	7.58017493	GO:0043500 muscle adaptation	Maintained_MAVA
0.00680194	5	136	9.67261905	GO:0003231 cardiac ventricle development	Maintained_MAVA

0.00782199	4	29	13.7566138	GO:0046033 AMP metabolic proc.	Maintained_MAVA
0.00810599	8	203	4.95238095	GO:0055001 muscle cell development	Maintained_MAVA
0.00906878	3	25	23.2142857	GO:0003299 muscle hypertrophy in response to stress	Maintained_MAVA
0.00906878	5	101	8.84353742	GO:0003300 cardiac muscle hypertrophy	Maintained_MAVA
0.00906878	3	26	23.2142857	GO:0014887 cardiac muscle adaptation	Maintained_MAVA
0.00048529	16	224	4.24489796	GO:0009260 ribonucleotide biosynthetic proc.	lost_MAVA
0.00048529	11	70	6.48526077	GO:0015986 proton motive force-driven ATP synthesis	lost_MAVA
0.00053624	16	231	4.12698413	GO:0046390 ribose phosphate biosynthetic proc.	lost_MAVA
0.00065229	7	28	10.8333333	GO:0006120 mitochondrial electron transport NADH to ubiquinone	lost_MAVA
0.00065229	15	230	4.06673618	GO:0006164 purine nucleotide biosynthetic proc.	lost_MAVA
0.00065229	15	210	4.18904404	GO:0009152 purine ribonucleotide biosynthetic proc.	lost_MAVA
0.00065229	16	285	3.83410138	GO:0009165 nucleotide biosynthetic proc.	lost_MAVA
0.00065229	16	288	3.83410138	GO:1901293 nucleoside phosphate biosynthetic proc.	lost_MAVA
0.0008312	12	124	4.89795918	GO:0009201 ribonucleoside triphosphate biosynthetic proc.	lost_MAVA
0.0008312	15	241	3.95136778	GO:0072522 purine-containing compound biosynthetic proc.	lost_MAVA
0.00120648	12	135	4.69172932	GO:0009142 nucleoside triphosphate biosynthetic proc.	lost_MAVA
0.00141987	13	143	4.23558897	GO:0006119 oxidative phosphorylation	lost_MAVA

0.00146961	11	107	4.86394558	GO:0006754 ATP biosynthetic proc.	lost_MAVA
0.00146961	12	144	4.45714286	GO:0009199 ribonucleoside triphosphate metabolic proc.	lost_MAVA
0.00146961	9	59	6.07792208	GO:0010257 NADH dehydrogenase complex assembly	lost_MAVA
0.00146961	11	105	4.86394558	GO:0022904 respiratory electron transport chain	lost_MAVA
0.00146961	9	59	6.07792208	GO:0032981 mitochondrial respiratory chain complex I assembly	lost_MAVA
0.00188272	11	118	4.69622332	GO:0009206 purine ribonucleoside triphosphate biosynthetic proc.	lost_MAVA
0.00197234	11	119	4.64285714	GO:0009145 purine nucleoside triphosphate biosynthetic proc.	lost_MAVA
0.00197234	9	67	5.7635468	GO:0019646 aerobic electron transport chain	lost_MAVA
0.00219738	14	445	3.63636364	GO:0060537 muscle tissue development	lost_MAVA
0.00311275	12	169	4.01544402	GO:0009141 nucleoside triphosphate metabolic proc.	lost_MAVA
0.00311275	17	340	3.00680272	GO:0015980 energy derivation by oxidation of organic compounds	lost_MAVA
0.00360125	11	136	4.25595238	GO:0009205 purine ribonucleoside triphosphate metabolic proc.	lost_MAVA
0.00360125	9	78	5.22321429	GO:0042775 mitochondrial ATP synthesis coupled electron transport	lost_MAVA
0.0037575	11	131	4.21207658	GO:0022900 electron transport chain	lost_MAVA
0.00393424	8	197	5.82633053	GO:0098739 import across plasma membrane	lost_MAVA
0.00450081	11	144	4.08571429	GO:0009144 purine nucleoside triphosphate metabolic proc.	lost_MAVA

0.00591935	13	197	3.42451874	GO:0009060 aerobic respiration	lost_MAVA
0.00640615	18	560	2.67428571	GO:0090407 organophosphate biosynthetic proc.	lost_MAVA
0.00819867	20	1131	2.45167374	GO:0034220 ion transmembrane transport	lost_MAVA
0.00922772	4	24	13.5064935	GO:0010884 positive reg. of lipid storage	lost_MAVA

<u>Supplemental Figure 1:</u> Myosin biochemical states in relation to fibre types in human XLMTM.

(A) represents the proportion of myosin molecules in the DRX state (P1) whilst (B) is the number of myosin heads in the SRX state (P2) for all patients. Note that data are separated according to fibre types (slow vs fast twitch fibres). Circles are individual muscle fibres from three controls and three patients.

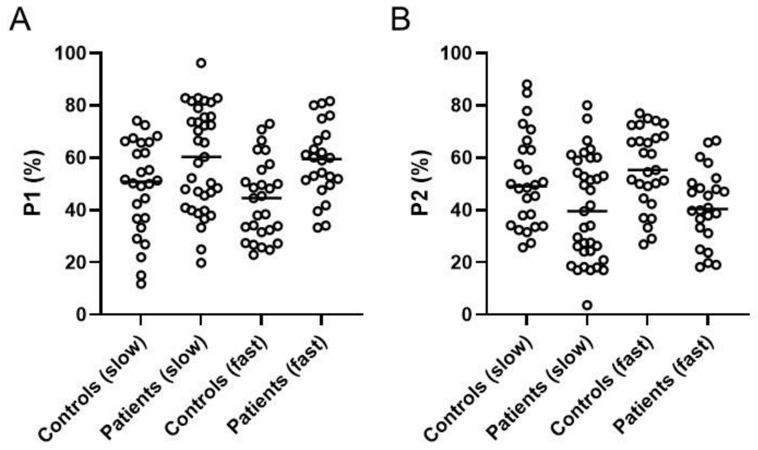

Supplement: Supplemental data [file jciinsight-10-194868-s073.pdf]
